# Identification of Regulatory Genes Implicated in Continuous Flowering of Longan (*Dimocarpus longan* L.)

**DOI:** 10.1371/journal.pone.0114568

**Published:** 2014-12-05

**Authors:** Tianqi Jia, Danfeng Wei, Shan Meng, Andrew C. Allan, Lihui Zeng

**Affiliations:** 1 College of Horticulture, Fujian Agriculture and Forestry University, Fuzhou, Fujian, P. R. China; 2 The New Zealand Institute for Plant & Food Research Limited (Plant & Food Research), Mt Albert, Auckland, New Zealand; National Institute of Plant Genome Research, India

## Abstract

Longan (*Dimocarpus longan* L.) is a tropical/subtropical fruit tree of significant economic importance in Southeast Asia. However, a lack of transcriptomic and genomic information hinders research on longan traits, such as the control of flowering. In this study, high-throughput RNA sequencing (RNA-Seq) was used to investigate differentially expressed genes between a unique longan cultivar ‘Sijimi’(S) which flowers throughout the year and a more typical cultivar ‘Lidongben’(L) which flowers only once in the season, with the aim of identifying candidate genes associated with continuous flowering. 36,527 and 40,982 unigenes were obtained by *de novo* assembly of the clean reads from cDNA libraries of L and S cultivars. Additionally 40,513 unigenes were assembled from combined reads of these libraries. A total of 32,475 unigenes were annotated by BLAST search to NCBI non-redundant protein (NR), Swiss-Prot, Clusters of Orthologous Groups (COGs) and Kyoto Encyclopedia of Genes and Genomes (KEGG) databases. Of these, almost fifteen thousand unigenes were identified as significantly differentially expressed genes (DEGs) by using Reads Per kb per Million reads (RPKM) method. A total of 6,415 DEGs were mapped to 128 KEGG pathways, and 8,743 DEGs were assigned to 54 Gene Ontology categories. After blasting the DEGs to public sequence databases, 539 potential flowering-related DEGs were identified. In addition, 107 flowering-time genes were identified in longan, their expression levels between two longan samples were compared by RPKM method, of which the expression levels of 15 were confirmed by real-time quantitative PCR. Our results suggest longan homologues of *SHORT VEGETATIVE PHASE* (*SVP*), *GIGANTEA* (*GI*), *F-BOX 1* (*FKF1*) and *EARLY FLOWERING 4* (*ELF4*) may be involved this flowering trait and *ELF4* may be a key gene. The identification of candidate genes related to continuous flowering will provide new insight into the molecular process of regulating flowering time in woody plants.

## Introduction

Longan, *Dimocarpus longan*, is a member of the family Sapindaceae. Longan trees are grown in many subtropical and tropical countries with majority of the production in Southeast Asia and Australia [1]. Planting area and yield in China have become the largest and highest in the world [2].

**Figure 1:**
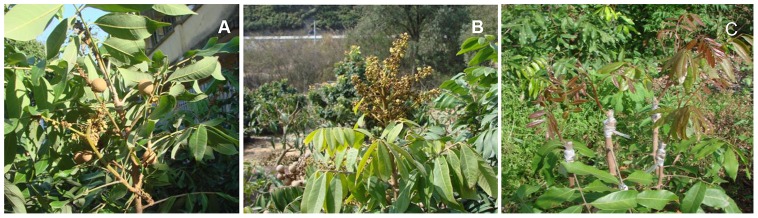
Sepecific flowering traits in ‘Sijimi’ longan. **A**. ‘Sijimi’ longan blossoming continuously, both terminal and axillary shoots can differentiate into inflorescences, flowers and fruits can be observed on one tree at the same time. B. When the growth is inhibited, apical buds develop into pure inflorescences. **C**. Scions of ‘Sijimi’ and ‘Lidongben’ were grafted on different branches of one rootstock plant.

Flowering is a key event in plant life, especially in fruit trees. Obtaining plants that flower over the year is the goal of many gardeners, so as to be able to achieve year-round fruit production. Usually, longan trees have a single spring flowering period, floral bud induction requires a period of low temperature and only the terminal meristem differentiates into an inflorescence. Off-season flowering in longan is achieved by chemical treatment with potassium chlorate (KClO_3_) application [3], [4]. However, the induction effect varies in different regions. One cultivar of longan, ‘Sijimi’, originating from the China (Guangxi Province) and Vietnam border region [5], has a continuous blossoming trait due to a spontaneous mutation. ‘Sijimi’ was found to have a closer genetic relationship with longan cultivars of Guangxi Province by use of molecular marker analysis and is clustered with Chinese cultivar groups [6], [7]. This cultivar blossoms and bears fruits throughout the year, under both tropical and subtropical conditions, with no requirement of environmental control. Both terminal and axillary buds of ‘Sijimi’ can differentiate into inflorescences. Flowers and fruits can be observed on one tree at the same time (**Figure 1A, B**). Therefore, ‘Sijimi’ has been successfully used to produce off-season fruits without KClO_3_ application. Furthermore, ‘Sijimi’ has a shorter grafting juvenile phase compared with normal longan cultivars. When ‘Sijimi’ scions are grafted on mature rootstocks in spring, sprouting shoots become mature in summer and bloom. In typical longan cultivars, flowering will occur at least two years after grafting on mature rootstocks. Based on the observation of specific flowering traits in ‘Sijimi’, we speculate that the mutation in ‘Sijimi’ gives mature shoots in mature trees or after grafting the capacity of continuous flowering.

Flowering is one of the most important developmental stages in plants, controlled by interactions between regulatory networks that control shoot development and those that mediate response to the environment. Photoperiod and temperature are two important environmental factors that affect plant flowering [8]. In *Arabidopsis*, the circadian clock and light signaling tightly control CONSTANS (CO) protein activity, and then accelerate flowering through the function of integrator genes like *FLOWERING LOCUS T* (*FT*), *SUPPRESSOR OF OVEREXPRESSION OF CONSTANS 1* (*SOC1*) and *LEAFY (LFY)* [9], [10]. The key regulator of the vernalization response in *Arabidopsis* is *FLOWERING LOCUS C (FLC)* [11]. *FLC* and *SHORT VEGETATIVE PHASE* (*SVP*), another transcriptional repressor, form a heterodimer under a wide range of cold conditions [12]. GA affects diverse biological processes, including flowering time. However, contrary to that seen in herbaceous species, GA may repress flowering in woody plants [13], [4].

Other information that plants use to provide input on initiation of flowering are endogenous changes, involving an autonomous pathway and age-related pathway which affect the juvenile to adult transition [14], [15]. Recently, mechanisms of age-dependent response to winter temperature in herbaceous perennial plants were reported [16], [17]. Two age-regulated microRNAs, miR156 and miR172, and their targets SQUAMOSA PROMOTER BINDING LIKE (SPL) and APETALA 2 (AP2) regulate the timing of sensitivity in response to vernalization [16], [17].

Perennial plants can flower both repeatedly and with seasonality. However, the regulatory pathway of seasonal flowering in perennial plants remains unclear. In rose and woodland strawberry, some cultivars also have the ability to flower continuously during a favorable season [18]. *KSN*, the *TERMINAL FLOWER 1* (*TFL1*) homologue in these two plants, is the regulator of continuous flowering, suggesting a new role of *TFL1* in perennial plants in the maintenance of vegetative growth and flowering seasonality [18]. In trifoliate orange a spontaneous mutant was found to have a short juvenile phase [19]. Deep sequencing and comparative gene expression analysis between wild type and precocious trifoliate orange during flower buds formation was used, but the gene associated with mutation remains unknown [19].

The longan cultivar ‘Lidongben’ originates from Fujian Province, Southern China. Although ‘Sijimi’ and ‘Lidongben’ originate from different regions of China, they have close genetic relationship [6], [7]. In the current study RNA sequencing was used to examine differentially expressed genes between ‘Sijimi’ longan and typical longan cultivar ‘Lidongben’,with the aim of identifying genes and regulatory pathways associated with the mutation in ‘Sijimi’. As it is difficult to obtain shoots with the same developing phase from mature trees of ‘Sijimi’ and normal longan cultivars, grafting newly-sprouting buds before maturity were used as material to eliminate the influence of inflorescence differentiation. Our results suggest longan homologues of *SVP*, *GI*, *FKF1* and *ELF4* may be involved in this flowering trait and *ELF4* may be a key gene. No differences were seen when comparing the *TFL1* homologue sequences between ‘Sijimi’ and ‘Lidongben’, indicating the mechanism in ‘Sijimi’ which is mutated is different from previously studied other perennial woody plants. The results will contribute to more understanding of seasonal flowering regulation in longan and other woody plants.

## Materials and Methods

### Plant materials

Longan samples of ‘Sijimi’ (S) and ‘Lidongben’ (L) were collected from the experimental fields of Fujian Academy of Agricultural Science in Fuzhou. Scions collected from mature trees of these two cultivars were grafted on different branches of mature rootstock plants (the cultivar ‘Fuyan’ of longan was used as rootstock) (**Figure 1C**). The terminal tips of newly-sprouting shoots were collected before maturity. Materials of the same cultivar from more than 20 rootstock plants were mixed. Using the same method, terminal buds from the next year grafting were used for real-time quantitative PCR. All materials were frozen in liquid nitrogen immediately and stored at −80°C for later use.

Total RNA was isolated using Universal Plant Maxi RNA Extraction Kit (BioTeke, China) according to the manufacturer’s instruction and treated with RNase-free DNase I (Takara Biotechnology, China). All RNA samples were quantified and examined for protein contamination (A260/280) and reagent contamination (A260/230) by a Nanodrop ND 1000 spectrophotometer. Thirty micrograms of total RNA were sent to Beijing Genome Institute (BGI) (Shenzhen, China) where the libraries were constructed and sequenced using Illumina HiSeq 2000. RNA-seq library was constructed as described by Liu et al. [20].

### Assembly and functional annotation

The cDNA fragments were approximately 200 bp in length and sequencing of cDNA libraries was performed by paired-end Illumina sequencing. *De novo* assembly of the short reads was carried out using SOAP*denovo* [21] as described. The obtained raw reads were pre-processed by removing adaptor sequences, low-quality reads and reads of larger than 5% unknown sequence. High-quality clean reads were assembled using Trinity [22]. The longest assembled sequences were termed contigs. Paired-end reads were then mapped back to contigs to detect contigs from the same transcript as well as the distances between these contigs. Sequences without Ns which could not be extended on either end were selected and defined as unigenes. Using the same strategy, unigenes of longan were obtained from both L and S libraries. The datasets are deposited in the NCBI’s SRA database with the accession numbers SRX479329 for the S library and SRX479332 for the L library, respectively.

The assembled unigenes were annotated by BLASTx alignment (E-value<0.00001) to protein databases including NCBI NR (http://www.ncbi.nlm.nih.gov), Swiss-Prot (http://www.expasy.ch/sprot), KEGG (http://www.genome.jp/kegg) and clusters of orthologous groups (COG database, http://www.ncbi.nlm.nih.gov/COG). Protein function was predicted based on features of the best BLASTX hits from the alignments. Sequence orientations were determined according to the best hit in the database. If results from different databases conflicted with each other, a priority order of NR, Swiss-Prot, KEGG and COG was followed. Orientation and CDS (coding region sequences) of sequences with no hit in blast were predicted using ESTScan [23] (http://www.ch.embnet.org/software/ESTScan.html). Original transcript sequences (5'-3') were provided if their orientations can be determined. Other sequences are provided as the assembler outputs.

For NR annotation, the Blast2GO program [24] was used to get GO annotation of unigenes. After obtaining a GO annotation for every unigene and differentially expressed genes, WEGO software [25] was used to classify GO function and to understand the distribution of gene functions of *Dimocarpus longan* from the macro level.

### Differential expression of unigenes

The reads for a specific transcript were counted by mapping reads to assembled unigene sequences using SOAP [26]. Unigene expression levels were calculated using the RPKM method (Reads Per kb per Million reads) [27]. The calculated gene expression can be used for comparing the difference of gene expression among samples.

The transcript fold change was calculated by the formula of log_2_(S_RPKM/L_RPKM). If the value of either L_RPKM or S_RPKM was zero, we used 0.01 instead of 0 to calculate the fold change. A modified Audic’s method [28] was used to analyze differential expression. The formula to calculate the probability of a specific gene being expressed equally between the two samples was defined as:
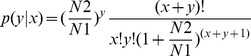



Where N1 and N2 indicate the total number of clean reads in L and S, respectively, and x and y indicate the number of mapped clean reads in each sample. FDR (False Discovery Rate) method was used to determine the threshold of the p-value in multiple tests [29]. In this study, we used FDR≤0.001 and the absolute value of log_2_Ratio≥1 as the threshold to judge the significance of differentially expressed genes.

### Gene Ontology analysis for significantly differentially expressed genes (DEGs)

All DEGs were mapped to GO terms in the database by calculating gene numbers for every term, followed by a test to find significantly enriched GO terms, using a formula for calculation as described by Liu et al. [20]. GO terms fulfilling this condition were defined as significantly enriched. This analysis is able to recognize the main biological function that each DEGs is assigned to. Pathway enrichment analysis was used to identify significantly enriched pathways which were differentially expressed. The calculating formula is the same as that in GO analysis. Pathway with FDR≤0.05 is called enriched significantly in differentially expressed genes.

### Identification of flowering-related genes and flowering-time genes

Published sequences of flowering-related genes or gene families were downloaded from NCBI as reference database. All DEGs were blasted to this reference database with expectation value le-5 to screen potential flowering-related genes and followed by GO enrichment analysis.

Furthermore, unigenes were identifed in the transcriptome database which were homologous to longan flowering-time genes, and confirmed by blasting to NCBI Nt and Nr database. The differential expression of identified flowering-time genes between two longan samples were determined by the value of log_2_(S_RPKM/L_RPKM).

### Real-time quantitative PCR verification

Fifteen flowering-time unigenes were selected to confirm their expression in ‘Sijimi’ and ‘Lidongben’ using real-time quantative PCR (RT qPCR). Longan *Actin* gene was used as the reference gene [30]. Specific primers for these genes were designed using DNAMAN 6.0 and primer sequences were listed in **Table S1**. All the primers were synthesized by Biosune in Shanghai, China.

The primary cDNA was synthesized from equal amounts of purified total RNA (1 µg) using the Prime Script RT reagent Kit (TaKaRa, China) follow by PCR analysis. Each PCR reaction mixture contained 2 µL of diluted cDNA (40 ng), 12.5 uL of SYBR Premix EX Taq (TaKaRa, China), 0.5 µL of each primer and 8.15 µL RNA-free H_2_O to a final volume of 25 µL. PCR was performed with following conditions: 95°C for 30 s, followed by 40 cycles of 95°C for 15 s, annealing temperature for 30 s and 72°C for 30 s in a CFX96 TouchReal-Time PCR Detection System (Bio-Rad, Hercules, CA, USA). Real-time qPCR was performed in triplicate for each sample, data were indicated as means ± SD (n = 3). Duncan’s multiple range test was used to analyze statistical significance of gene expression difference between two sample based on average expression level in each sample (P≤0.01).

## Results

### Illumina sequencing and reads assembly

**Figure 2:**
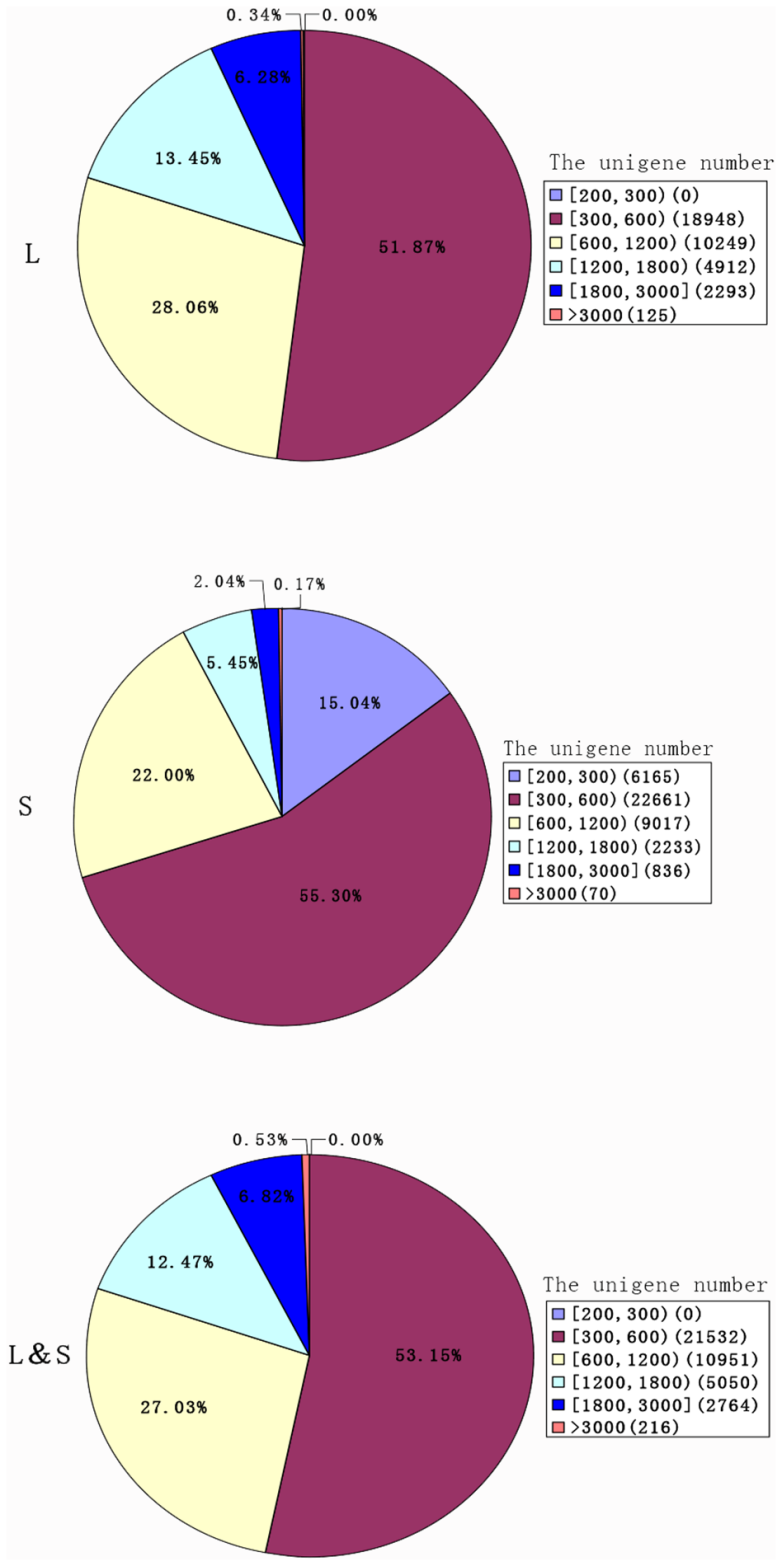
Length distribution of unigenes after *de novo* assembly using the reads from S, L and S&L libraries. Numbers in square brackets (e.g., [200, 300] and [1800, 3000]) indicate the range of unigene length, ‘>3000’indicates unigenes longer than 3,000 bp. Numbers in parentheses (e.g., (6, 165) and (22, 661)) indicate the total number of unigenes falling in this length range.

In order to determine the differential gene expression between the ‘Lidongben’(L) and ‘Sijimi’ (S) longan cultivars, cDNA samples from each were sequenced using the Illumina sequencing platform. The quality of the reads was evaluated using the base-calling quality scores from the Illumina’s base-caller. Over 96% of the clean reads had Phred-like quality scores at the Q20 level (a sequencing error probability of 0.01). 25,603,968 and 27,723,240 clean reads of 90 bp were obtained from L and S and were used to assemble 74,185 contigs in L and 75,548 contigs in S, respectively. With the approach of paired-end read joining and gap-filling, the contigs from above were further assembled into 36,527 unigenes with a mean size of 701 bp in L and 40,982 unigenes with a mean size of 481 bp in S. To reduce the errors and biases during RNA sequencing and assembly process, the raw reads from L (26 million) and S (28 million) were combined, and 40,513 unigenes were assembled with a mean size of 703 bp (**Table 1**). The length distributions of unigenes from the three combinations (L, S and L&S) were shown in **Figure 2**.

**Table 1:** Summary of sequence assembling results after Illumina sequencing of cDNA libraries from ‘Sijimi’ and ‘Lidongben’ of longan.

	L	S	L&S
**Total Clean base pairs (bp)**	2,304,357,120	2,495,091,600	
**Total number of clean Reads**	25,603,968	27,723,240	
**GC percentage**	46.75%	49.40%	
**Mean length of contigs (bp)**	371	298	
**The number of contigs**	74,185	75,548	
**Total length of contigs (bp)**	27,546,858	22,544,986	
**Mean length of unigenes (bp)**	701	481	703
**The number of unigenes**	36,527	40,982	40,513
**Total length of unigenes (bp)**	25,596,008	19,703,818	28,468,366

### Functional annotation of unigenes

BLAST search was used against the public protein and nucleotide databases. Of the 36,527 L and 40,982 S unigenes, 29,687 and 33,855 unigenes had at least one hit within these databases, respectively. Of the 40,513 L&S unigenes, 32,475 (80.16%) were annotated with a Blast match. The remaining unigenes (18.73%, 17.39% from L, S and 19.84% from L&S unigenes respectively) with no hit in any database could be longan-specific genes, or genes with homologues in other species whose corresponding biological functions have not been identified (**Table 1**).

Furthermore, after blasting the longan (L&S) unigenes against NR, Swiss-Prot, KEGG, COG protein databases and GO annotation, a total of 32,475 CDS were obtained (**Table 2**). Unigenes with no hits to any of these databases were blasted by ESTScan to predict the nucleotide (5'-3') and amino acid sequences of the coding regions, with 1,452 genes were identified using this method.

**Table 2:** Summary of functional annotation of assembled unigenes.

	40,513 unigenes (L&S)		36,527 unigenes (L)		40,982 unigenes (S)	
Database	Annotated(n)	%	Annotated(n)	%	Annotated(n)	%
**NR**	31,221	77.06	28,665	78.48	32,620	79.60
**SwissProt**	19,028	46.97	17,114	46.85	19,056	46.50
**KEGG**	17,410	42.97	15,244	41.73	17,255	42.10
**COG**	10,646	26.28	10,023	27.44	9,356	22.83
**GO**	24,855	61.35	12,397	33.94	25,212	61.52
**NT**	27,716	68.41	25,737	70.46	28,797	70.27
**Total**	32,475	80.16	29,687	81.27	33,855	82.61

### Differential expression and pathway analysis in L and S

**Figure 3:**
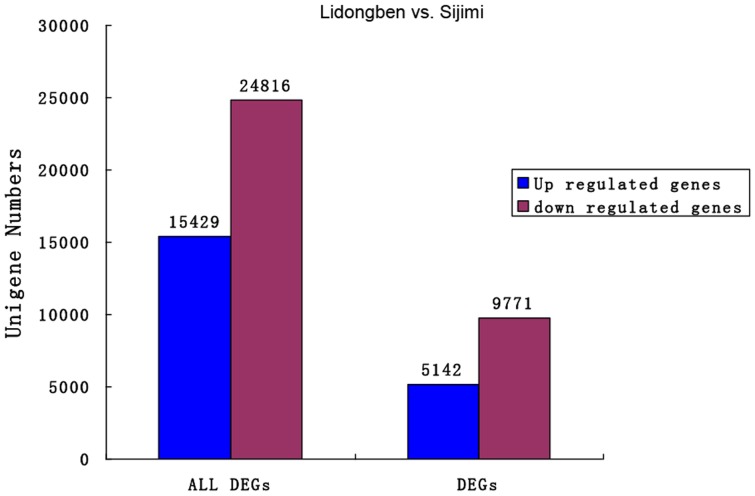
Differentially expressed genes between the ‘Lidongben’ and ‘Sijimi’. ‘ALL DEGs’ indicates those unigenes showing differential expression between the two samples, ‘DEGs’ indicates those unigenes with FDR≤ 0.001 and |log2Ratio| ≥ 1. The numbers of genes up-regulated and down-regulated in the ‘Sijimi’ relative to ‘Lidongben’ are indicated above the blue or red bars, respectively.

We developed an algorithm (see Methods) to identify longan unigenes differentially expressed between L and S samples. Comparing S to L, 15,429 unigenes were up-regulated and 24,816 were down-regulated (**Figure 3**). The significance of gene expression differences was judged by using the threshold of false discovery rate (FDR≤0.001) and the absolute value of log_2_Ratio (≥1). 14,913 significantly differentially expressed genes (DEGs) were obtained between the two samples, including 9,771 down-regulated and 5,142 up-regulated genes (**Figure 3**). The number of down-regulated genes was almost 2-fold that of up-regulated genes.

**Figure 4:**
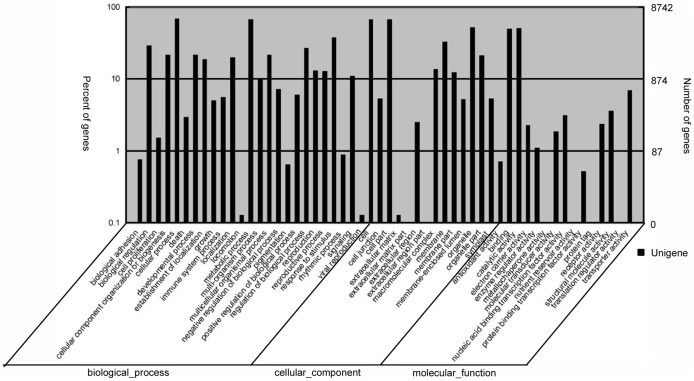
Histogram representation of Gene Ontology classification of DEGs. GO categories, shown in the x-axis, are grouped into three main ontologies: biological process, cellular component and molecular function. The right y-axis indicates the number of genes in each category, while the left y-axis indicates the percentage of total genes in that category.

To evaluate the potential functions of genes with significant transcriptional changes between the L and S, 8,743 of 14,913 DEGs were categorized into 54 GO terms consisting of three domains: biological process, cellular component and molecular function. It was clear that the dominant distributions were from ‘Cellular process’, ‘Metabolic process’, ‘Cell’ and ‘Cell part’ terms. We also observed a high percentage of genes assigned to ‘Organelle’, ‘Binding’, ‘Catalytic activity’, ‘Response to stimulus’, ‘Membrane’, ‘Biological regulation’ and ‘Regulation of biological process’. A few genes were assigned to ‘Locomotion’, ‘Viral reproduction’, ‘Extracellular matrix’, ‘Extracellular region part’, ‘Extracellular matrix part’, ‘Metallochaperone activity’, ‘Nutrient reservoir activity’, ‘Protein tag’ and ‘Translation regulator activity’ (**Figure 4**).

**Figure 5:**
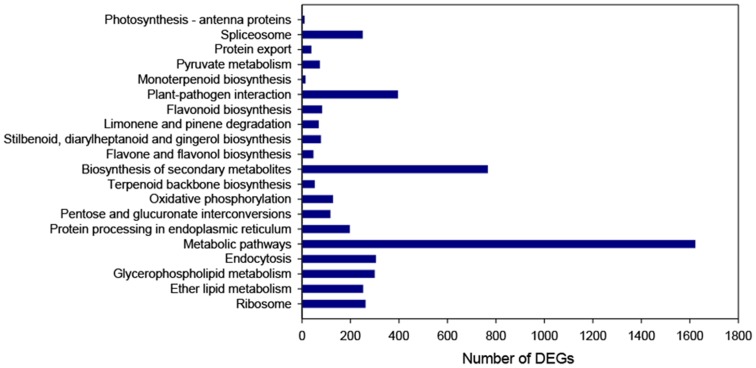
The top 20 pathway assignments of DEGs based on KEGG. The x-axis indicates the number of DEGs in a category. The y-axis from bottom to top shows the top 20 pathways.

Pathway-based analysis helps to further understand the product of a genes biological function. 14,913 of DEGs were mapped to the KEGG database, a total of 128 different pathways were found in this study related to 6,415 unigenes (**Table S2**). The maps with highest unigene representation were metabolic pathways (1,625 unigenes), followed by the biosynthesis of secondary metabolites (769 unigenes). Lipid metabolism, glycerophospholipid metabolism, pentose and glucuronate interconversions, oxidative phosphorylation and the spliceosome were included in the top 20 pathways (**Figure 5**). Specific pathways were found that are implicated in flowering, such as plant hormone signal transduction, the spliceosome, circadian rhythm, and starch and sucrose metabolism.

### Identification of flowering-related and flowering-time genes

After blast-based searching of the 14,913 DEGs to reference database, 539 potential flowering-related genes were found. GO Enrichment analysis indicated that these unigenes significantly enrich in terms of ‘nucleus’, ‘sequence-specific DNA binding transcription factor activity’, ‘DNA binding’, ‘protein dimerization activity’, ‘membrane’ and ‘regulation of transcription, DNA dependent’ (**Figure S1**).

Flowering time of fruit treeshas been reported to be affected by environmental and biological factors such as photoperiod, temperature, plant age and gibberellic acid (GA) [31], [32]. In order to identify DEGs related to flowering signal transduction and integration, 107 putative flowering-time homologues including differentially transcribed genes were identified from longan unigenes in this study. These were further catalogued into circadian clock & photoperiod pathway, vernalization pathway, autonomous pathway, GA pathway, age-related pathway and floral pathway integrator genes (**Table 3** **and** **Table S3**).

**Table 3:** Longan unigenes that share homology to flowering-time genes of other plants.

GeneID	Gene Length	Reads(L)	Reads(S)	log_2_(S_RPKM/L_RPKM)	GenBank Accession number	Identity (%)	E-value	Protein function
**Circadian clock & Photoperiod pathway**								
CL703.Contig1	2649	811	728	−0.3214	XM_002285756.2	1960/2433 (81%)	0	*CRY1(Vitis vinifera)*
CL903.Contig2	1333	115	74	−0.8016	XM_002285133.2	914/1161 (79%)	0	*CRY2(Vitis vinifera)*
Unigene1512	320	47	41	−0.3626	XM_002285133.2	270/320 (84%)	7E-94	*CRY2 (Vitis vinifera)*
Unigene6715	361	20	20	−0.1656	XM_002280735.1	245/351 (70%)	8E-37	*CRY3(Vitis vinifera)*
Unigene7391	1094	254	110	−1.3729	XM_002280735.1	702/856 (82%)	0	*CRY3(Vitis vinifera)*
Unigene8667	2200	212	204	−0.2211	XM_002278574.2	1637/2007 (82%)	0	*PHYA(Vitis vinifera)*
Unigene1734	2472	335	498	0.4064	XM_002519184.1	1562/1873 (83%)	0	*PHYB(Ricinus communis)*
Unigene14185	1234	224	151	−0.7346	NM_122975.2	523/796 (66%)	1E-41	*PHYC(Arabidopsis thaliana)*
Unigene8582	639	28	15	−1.0661	NM_122975.2	452/643 (70%)	8E-72	*PHYC(Arabidopsis thaliana)*
Unigene12755	310	22	6	−2.0401	XM_002271635.2	243/293 (83%)	3E-79	*PHYE(Vitis vinifera)*
Unigene27248	250	10	3	−1.9025	XM_002271635.2	195/235 (83%)	2E-61	*PHYE(Vitis vinifera)*
Unigene2802	797	136	45	−1.7612	XM_002271635.2	369/502 (74%)	4E-77	*PHYE(Vitis vinifera)*
Unigene11739	594	72	109	0.4326	NM_001161311.1	295/363 (81%)	2E-92	*ZTL()Arabidopsis thaliana*
Unigene9169	1986	461	192	−1.4293	JQ424912.1	1056/1275 (83%)	0	*ZTL(Nicotiana attenuata)*
CL1764.Contig1	3005	520	1241	1.0893	AY611029.1	1299/1722 (75%)	0	*LHY(Castanea sativa)*
CL1764.Contig2	2789	67	99	0.3977	AY611029.1	925/1252 (74%)	0	*LHY(Castanea sativa)*
Unigene12806	1266	175	222	0.1776	AY611028.1	599/758 (79%)	5E-179	*TOC1(Castanea sativa)*
Unigene7343	682	35	92	1.2287	AY611028.1	534/685(78%)	1E-152	*TOC1(Castanea sativa)*
Unigene836	829	16	127	2.823	HQ833381.1	712/828 (86%)	0	*FKF1(Populus tremula)*
Unigene12571	1550	78	308	1.8158	XM_002264719.1	969/1120(87%)	0	*GI(Vitis vinifera)*
Unigene3692	2414	528	1495	1.3359	XM_002264719.1	1691/2111 (80%)	0	*GI(Vitis vinifera)*
Unigene5963	295	29	74	1.1859	XM_002270697.1	157/233(67%)	1E-08	*ELF4(Vitis vinifera)*
Unigene4309	688	18	1306	6.0154	XM_003552327.1	118/150(79%)	1E-25	*ELF4(Vitis vinifera)*
Unigene5237	2243	312	259	−0.4342	XM_002278541.1	1385/1913 (72%)	0	*ELF3(Vitis vinifera)*
CL3477.Contig1	2611	665	293	−1.3481	XM_003545549.1	1572/1914(82%)	0	*COP1(Glycine max*)
Unigene13951	1520	48	149	1.4686	XM_002267627.1	745/1061(70%)	6E-123	*COP1(Vitis vinifera*)
Unigene14978	522	44	54	0.1298	XM_002268454.1	159/199 (80%)	2E-41	*CO(Vitis vinifera)*
Unigene15508	1505	172	234	0.2785	XM_002264470.2	547/674 (81%)	5E-180	*CO(Vitis vinifera)*
Unigene15973	993	126	80	−0.821	NM_102570.3	192/247 (78%)	1E-46	*CO(Arabidopsis thaliana)*
Unigene16245	557	37	8	−2.375	XM_002263577.1	129/151 (85%)	7E-40	*CO(Vitis vinifera)*
Unigene3300	1546	4254	5317	0.1562	EU939303.1	773/1019 (76%)	0	*CO(Prunus persica)*
Unigene5163	433	20	80	1.8344	XM_002268454.1	134/177 (76%)	2E-25	*CO(Vitis vinifera)*
Unigene7324	1122	198	91	−1.2872	AY515150.1	763/1086 (70%)	7E-145	*CO(Populus deltoides)*
Unigene7468	1044	107	306	1.3503	XM_002263577.1	446/581 (77%)	7E-119	*CO(Vitis vinifera)*
**Vernalization pathway**								
Unigene3148	758	196	141	−0.6408	XM_002282436.2	581/683 (85%)	0	*FIE(Vitis vinifera)*
Unigene8962	744	167	133	−0.494	EU439048.2	261/329 (79%)	2E-68	*FIE(Hieracium pilosella)*
Unigene11356	450	15	42	1.3198	XM_002283740.1	246/305 (81%)	2E-71	*VIN3(Vitis vinifera)*
Unigene13871	1792	223	125	−1.0007	XM_002281310.2	1168/1574 (74%)	0	*VIN3(Vitis vinifera)*
Unigene15146	957	139	81	−0.9447	XM_002283740.1	513/732 (70%)	2E-88	*VIN3(Vitis vinifera)*
Unigene262	630	40	31	−0.5333	XM_002281310.2	378/465 (81%)	4E-119	*VIN3(Vitis vinifera)*
Unigene26983	293	10	6	−0.9026	XM_002270299.2	172/225 (76%)	8E-36	*VIN3(Vitis vinifera)*
Unigene5939	286	24	68	1.3369	XM_002283740.1	207/289 (72%)	9E-35	*VIN3(Vitis vinifera)*
Unigene6082	272	8	23	1.358	XM_002270299.2	194/246 (79%)	5E-50	*VIN3(Vitis vinifera)*
Unigene9234	1292	244	317	0.212	XM_002270299.2	840/1169 (72%)	1E-174	*VIN3(Vitis vinifera)*
CL3036.Contig2	1224	167	185	−0.0179	AF289052.1	295/385 (77%)	1E-73	*VRN1(Arabidopsis thaliana)*
Unigene1789	1824	248	246	−0.1773	XM_002532306.1	461/553 (83%)	2E-147	*EMF2(Ricinus communis)*
Unigene6914	2362	527	194	−1.6073	XM_002281643.2	1512/1992(76%)	0	*EMF2 (Vitis vinifera)*
Unigene11831	1322	507	664	0.2236	XM_002270351.2	724/894 (81%)	0	*SE(Vitis vinifera)*
Unigene7433	1491	327	420	0.1955	XM_002270351.2	770/931 (83%)	0	*SE(Vitis vinifera)*
Unigene13940	1135	102	109	−0.0698	GQ177180.1	83/109 (76%)	1E-09	*FES1(Arabidopsis thaliana)*
Unigene5288	866	219	192	−0.3554	GQ177180.1	55/73 (75%)	0.009	*FES1(Arabidopsis thaliana)*
CL67.Contig1	2080	510	442	−0.3721	XM_003634798.1	1413/1799 (79%)	0	*FRI(Vitis vinifera)*
CL67.Contig2	2142	152	1077	2.6593	XM_003634798.1	1395/1773 (79%)	0	*FRI(Vitis vinifera)*
**Autonomous pathway**								
Unigene3715	867	48	64	0.2494	XM_002279479.2	349/450 (78%)	9E-98	*FCA(Vitis vinifera)*
Unigene12093	1981	332	555	0.5757	XM_002279479.2	1178/1656 (71%)	0	*FCA(Vitis vinifera)*
Unigene8904	828	60	69	0.036	XM_002269409.2	421/617 (68%)	8E-48	*LD(Vitis vinifera)*
Unigene10275	587	25	173	2.6252	XM_002267668.2	292/351 (83%)	3E-96	*FY(Vitis vinifera)*
Unigene13338	749	100	243	1.1154	XM_002267668.2	617/773 (80%)	0	*FY(Vitis vinifera)*
Unigene26240	461	27	11	−1.461	XM_002267668.2	409/461 (89%)	2E-167	*FY(Vitis vinifera)*
Unigene6611	362	52	119	1.0288	XM_002267668.2	82/99 (83%)	2E-18	*FY(Vitis vinifera)*
CL1325.Contig2	853	309	184	−0.9135	FJ862913.1	852/853 (99%)	0	*FVE(Dimocarpus longan)*
Unigene3138	672	442	436	−0.1853	FJ862913.1	591/594 (99%)	0	*FVE(Dimocarpus longan)*
Unigene18631	515	7	32	2.027	NM_111874.4	343/516 (66%)	2E-33	*FLD(Arabidopsis thaliana)*
Unigene15818	1379	86	32	−1.5919	EF643229.1	249/313(80%)	2E-70	*FLD(Phaseolus vulgaris)*
Unigene18312	384	12	21	0.6418	XM_003537882.1	302/383(79%)	5E-85	*FPA(Glycine max)*
Unigene2059	451	18	45	1.1563	XM_003601264.1	290/367(79%)	3E-82	*FPA(Medicago truncatula)*
Unigene5074	377	25	63	1.1678	XM_003537882.1	182/241(76%)	2E-38	*FPA(Glycine max)*
Unigene7507	1276	150	250	0.5714	NM_129902.2	106/132(80%)	4E-23	*FPA(Arabidopsis thaliana)*
Unigene9309	435	28	53	0.7549	XM_004507289.1	274/338(81%)	2E-84	*FPA(Cicer arietinum)*
**GA pathway**								
Unigene12858	440	13	32	1.134	XM_002303240.1	179/242 (74%)	2E-15	*GA3ox(Populus trichocarpa)*
Unigene21359	238	5	11	0.9719	XM_002299583.1	189/237 (80%)	8E-42	*GA3ox(Populus trichocarpa)*
Unigene25332	300	17	7	−1.4457	XM_002303240.1	244/300 (81%)	3E-64	*GA3ox(Populus trichocarpa)*
Unigene25388	546	25	8	−1.8094	XM_002301494.1	230/285 (81%)	5E-57	*GA2ox(Populus trichocarpa)*
Unigene27444	232	10	31	1.4667	XM_002305668.1	193/225 (86%)	1E-62	*GA2ox(Populus trichocarpa)*
Unigene22418	254	5	6	0.0975	XM_002310487.1	201/254 (79%)	3E-53	*GA20ox(Populus trichocarpa)*
Unigene4959	305	17	83	2.122	AJ250187.1	186/245 (76%)	4E-39	*GA20ox(Citrus sinensis x Poncirus trifoliata)*
Unigene213	1107	141	67	−1.2391	AK221314.1	265/350 (76%)	8E-62	*SPY(Arabidopsis thaliana)*
Unigene5529	559	27	16	−0.9205	AK221314.1	466/557 (84%)	2E-166	*SPY(Arabidopsis thaliana)*
Unigene15359	508	34	102	1.4194	EU878416.1	426/507 (84%)	8E-153	*SPY(Sinningia speciosa)*
Unigene15577	1333	231	115	−1.1719	XM_002309855.1	842/1264 (67%)	3E-87	*PHOR1(Populus trichocarpa)*
CL141.Contig1	1254	152	18	−3.2436	XM_002266231.2	756/980 (77%)	8E-157	*GAI (Vitis vinifera)*
Unigene15619	1601	469	239	−1.1382	XM_002271664.2	924/1165 (79%)	0	*SLY1(Vitis vinifera)*
Unigene1628	937	800	420	−1.0952	XM_003632462.1	195/245 (80%)	1E-52	*SLY2 (Vitis vinifera)*
**Age-related pathway**								
Unigene12569	1076	958	514	−1.0639	NM_128940.2	267/383 (70%)	2E-36	*SPL3(Arabidopsis thaliana)*
Unigene4477	1690	3031	2844	−0.2575	FJ502237.1	927/1225 (76%)	0	*SPL9(Poncirus trifoliata)*
CL1113.Contig1	903	3	3	−0.1657	XM_003520593.1	307/368(83%)	4E-104	*AP2(Glycine max)*
CL1113.Contig2	823	3	2	−0.751	XM_003520593.1	307/368(83%)	3E-104	*AP2(Glycine max)*
CL1113.Contig3	814	2	1	−1.1657	XM_003554309.1	306/368(83%)	4E-103	*AP2(Glycine max)*
CL1113.Contig4	734	2	1	−1.1656	XM_003520593.1	307/368(83%)	3E-104	*AP2(Glycine max)*
CL1113.Contig5	723	2	1	−1.166	XM_003520593.1	375/455(82%)	5E-126	*AP2(Glycine max)*
CL1113.Contig6	643	2	1	−1.1655	XM_003520593.1	410/506(81%)	3E-129	*AP2(Glycine max)*
CL1113.Contig7	732	3	2	−0.7507	XM_003520593.1	410/506(81%)	3E-129	*AP2(Glycine max)*
CL1113.Contig8	812	3	2	−0.7505	XM_003520593.1	375/455(82%)	6E-126	*AP2(Glycine max)*
**Floral pathway integrator genes**								
Unigene8992	970	288	143	−1.1757	EU497678.1	396/517 (77%)	2E-76	*FLC(Poncirus trifoliata)*
Unigene10736	1206	218	128	−0.9338	EU032532.1	500/629 (79%)	2E-153	*SOC1(Citrus sinensis)*
Unigene13279	1054	621	283	−1.2994	EU032531.1	612/794 (77%)	1E-167	*SOC*1(Citrus sinensis)
Unigene15717	1083	329	309	−0.2561	JN214349.1	872/903 (97%)	0	*AP1(Litchi chinensis)*
Unigene14549	330	20	48	1.0974	EF489297.1	302/304 (99%)	2E-150	*LFY(Dimocarpus longan)*
Unigene6027	525	29	37	0.1859	XM_002276784.2	383/494 (78%)	8E-102	*TFL1 (Vitis vinifera)*
Unigene6475	1241	471	296	−0.8357	AY344244.1	471/587 (80%)	8E-145	*TFL1(Citrus sinensis)*
CL2719.Contig1	1271	145	58	−1.4875	XM_002269259.2	526/662 (79%)	2E-131	*SVP(Vitis vinifera)*
CL2719.Contig2	1367	76	14	−2.6062	XM_002269259.2	526/662 (79%)	3E-131	*SVP(Vitis vinifera)*
CL2719.Contig3	1532	56	50	−0.3291	XM_002269259.2	526/662 (79%)	3E-131	*SVP(Vitis vinifera)*
CL645.Contig1	970	205	86	−1.4188	JF838219.1	156/183 (85%)	2E-46	*SVP(Actinidia chinensis)*
CL645.Contig2	1259	303	62	−2.4546	XM_002262853.1	160/190 (84%)	4E-45	*SVP(Vitis vinifera)*
Unigene17296	897	359	104	−1.953	XM_002262853.1	160/190 (84%)	3E-45	*SVP(Vitis vinifera)*
Unigene26608	418	77	30	−1.5255	JF838219.1	156/183 (85%)	9E-47	*SVP(Actinidia chinensis)*

In circadian clock & photoperiod pathway, eight longan unigenes showed significant similarity to Arabidopsis *CO*. Some genes in light signal pathway and in circadian clock loops were also identified. However *CIRCADIAN CLOCK-ASSOCIATED 1* (*CCA1*), *PSEUDO RESPONSE REGULATOR 7/9* (*PRR7/9*) and *CCA1 HIKING EXPEDITION* (*CHE*) were apparently absent. F-BOX 1 (FKF1) and GIGANTEA (GI) proteins, which are downstream of the circadian clock, play major roles in facilitating the *CO* expression [10], [33]. Two putative *GI* homologues and one *FKF1* homologue were identified in longan. According to the calculation of transcript fold change (log_2_(S_RPKM/L_RPKM)), the expression levels of GI and FKF1, were up-regulated in the S sample. Especially, the value of log_2_(S_RPKM/L_RPKM) of one gene (Unigene4309) encoding *EARLY FLOWERING 4* (*ELF4*), which is involved in many of the same physiological processes as *GI* [34], reached 6.0154, markedly large increased in the S sample compared to the L sample. Another longan homolog of *ELF4*, named Unigene5963, also showed an increase in transcription level.

The epigenetic silencing of the *FLOWERING LOCUS C* (*FLC*) [35], [36] is central to the vernalization process [37]. One putative homologue for the *FLC* sequence was found in longan, and showed a decrease in transcript abundance in the S sample. *FLOWERING LOCUS M* (*FLM*/*MAF1*) and *MADS-AFFECTING FLOWERING 2* (*MAF2*), clade members of *FLC*, were not found in our database. FRIGIDA (FRI) is the activator of *FLC* in *Arabidopsis* [38]. However, longan *FRI* (CL67.Contig2) had higher expression levels in the S sample. Genes which negatively regulate *FLC* in vernalization pathway were also present in the two samples, like *VERNALIZATION INSENSITIVE 3* (*VIN3*), these genes did not show significant expression change between two genotypes.

*SVP* is a flowering repressor and central regulator of the flowering regulatory network [39]. Multiple copies of *SVP* were found in longan, and their expression levels were all down-regulated. Both *SVP* and *FLC* are targets of genes in the autonomous pathway [40], [41]. Several unigenes in this pathway, such as *FLOWERING LOCUS Y* (*FY*) (Unigene10275) and *FLOWERING LOCUS D* (*FLD*) (Unigene18631) showed higher transcription levels in S.

GA promotes flowering in *Arabidopsis* under SD [42]. Several putative *GA3ox*, *GA20ox* and *GA2ox* genes were identified in the two samples. Most of their reads were relatively lower. However, the expression of two *GA3ox* (Unigene12858, Unigene21359) and two *GA20ox* (Unigene4959, Unigene22418) unigenes were up-regulated. Candidate unigenes for the GA signal were also examined. *SPINDLY* (*SPY*), *PHOTOPERIOD RESPONSIVE 1* (*PHOR1*), *GIBBERELLIC ACID INSENSITIVE* (*GAI*), *SLEEPY1* (*SLY1*) and *SLEEP2* (*SLY2*) were found. With the exception of *SPY* (Unigene15359), the expression levels of these six genes were all down-regulated in S.

An additional flowering pathway, termed the age-related pathway, represents a developmental process with parts that are independent of environmental variables [14]. Sequences corresponding to *SQUAMOSA PROMOTER BINDING PROTEIN-LIKE 9* (*SPL9*) and *SPL3*, the targets of MicroRNA156 were identified in two samples, both of their expression levels were down-regulated. One cluster of genes (CL1113) was found corresponding to the *APETALA 2* (*AP2*) gene, the target of MiRNA172 and the repressor of *FT*. All contigs in this cluster seemed to be differentially transcribed and their reads were relatively low.

Two putative *TFL1* genes were found in longan, the expression of Unigene6027 was slightly up-regulated in the S sample and the expression of Unigene6475 was down-regulated. Cloning and resequencing of the cDNA and genomic DNA sequence of Longan *TFL1* (Unigene6475) coding region from ‘Lidongben’ and ‘Sijimi’ showed no coding differences (**Figure S2**). Other floral integrators, such as *LFY*, *APETALA1* (*AP1*) and *SOC1* did not show significantly differential expression between the two samples. Expression of *FT* was not observed in these libraries, although three published longan *FTs* (*DlFT1*, *DlFT2* and *DlFT3* [43], [44]) were all expressed in leaf tissues (not shown).

**Figure 6:**
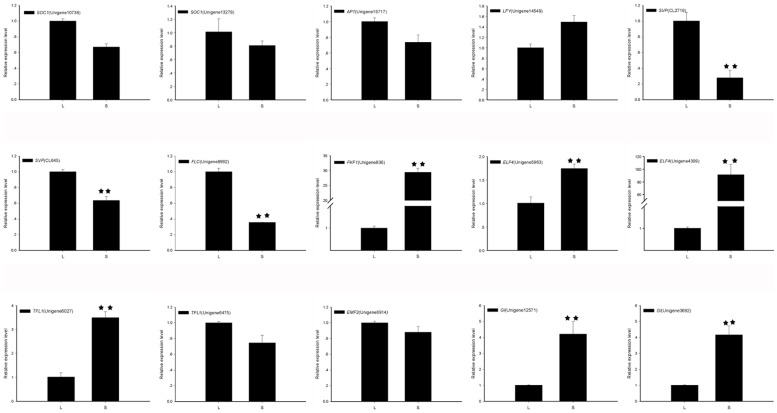
Real-time quantitative RT-PCR confirmation of the differentially expressed genes between ‘Lidongben’ and ‘Sijimi’. Relative transcript levels are calculated by real-time PCR with β-actin as the standard. Data are means ± SD of three separate measurements. Duncan’s multiple range test between two samples, ** P ≤0.01.

Differentially expressed genes revealed by transcriptome analysis were confirmed by real-time quantitative PCR. qPCR was done with sample collections from another year, to futher validate RNA-seq results. Fifteen flowering-time genes were chosen including floral pathway integrator genes and their family members **(** **Figure 6** **)**. The RT-qPCR results showed a similar trend for all tested unigenes in L and S samples. For example, of the two putative *TFL1* genes, the expression of Unigene6475 was down-regulated and the expression of Unigene6027 was up-regulated in S, consistent with the transcriptome data. The expression level of *ELF4* (Unigene4309) was increased nearly 90 times in the S sample compared with the L sample as analyzed by real-time PCR, consistent with transcriptome data which showed 72.5 times higher expression in the S sample compared with the L sample. Genes having statistically significant differences in transcription levels are shown in Figure 6.

## Discussion

Over the past decade, the development of genomic and transcriptomic technologies has contributed to a better understanding of floral biology at the molecular level. However, most of our knowledge about flower induction has arisen from studying flowering regulatory genes in *Arabidopsis thaliana*. Although these genes appear to be conserved in woody species [45], the regulatory pathways of flowering in woody perennials show remarkable differences [19], especially in the regulation of seasonal flowering.

To identify candidate genes associated with continuous flowering and obtain more insight into the molecular regulation of flowering seasonality in longan, genome-wide gene expression profiling was used to compare ‘Sijimi’ longan which has a continuous flowering habit and typical longan cultivar ‘Lidongben’. Using the SOAP*denovo* software, we generated a total of 40,513 unigenes from the two samples. Using this dataset, 14,913 significantly differentially expressed genes were identified, among them 539 flowering-related genes were identified.

Over one hundred putative homologues of flowering-time genes in longan were identified and their transcript abundance was compared, including floral integrator genes such as *LFY*, *AP1* and *SOC1*. However, *FT* was not found in the two libraries. Three longan *FT*-like genes, *DlFT1 DlFT2* and *DlFT3*, with flower promotion and repression functions in *Arabidopsis* have been described [43], [44]. With KClO_3_ treatment, *DlFT* transcripts were detected in mature leaves, and expression in other tissues including various stages of bud development and roots was not detected [44]. In *Arabidopsis*, FT induces flowering when expressed either in leaves or the shoot apical meristem (SAM) [46]. Experiments have demonstrated that FT moves from leaves to the SAM inducing flowering [47], [48]. Also, in grafts of *Cucurbita moschata*, movement of an FT ortholog across a graft junction in the phloem system correlated with flowering [49]. Our results suggest that in the terminal tips of newly-sprouting longan shoots, FT has either not been expressed or imported from surrounding leaves. The expression pattern of *DlFTs* during flowering of longan needs further investigation.

*SOC1* integrates multiple flowering signals and activates *LFY*, a floral meristem identity gene [15]. The expression levels of *SOC1*, *LFY* and another floral meristem identity gene *AP1* were not altered significantly between the two samples, consistent with the developmental stage of materials used in this study. These results also suggest that these integrator genes are not associated with the continuous flowering trait of ‘Sijimi’.

In *Arabidopsis*, the GA pathway actively promotes flowering under SD conditions, by up-regulating one or both of the genes *LFY* [50] and *SOC1* [51]. The literature on the role of GAs in the floral initiation of woody perennials is large but inconsistent [52]. GAs inhibit floral initiation in mango [53] and citrus [13]. In longan, high GA_1_, GA_3_, GA_19_ and GA_20_ levels in the shoot tips contribute to floral bud induction [54]. However, it has also been demonstrated that GA inhibits flowering in longan as has been found for other fruit trees [4], [32]. In our results, GA biosynthetic genes, *GA20ox* and *GA3ox* were up-regulated in the S cultivar. However, the reads of all these genes were very low in the two samples, suggesting the GA pathway may not be dominant for flowering in longan[55]. The GA signal promotes growth by initiating the degradation of DELLA proteins [56]. In poplar, the higher expression of *GA2ox* and *GA INSENSITIVE* (*GAI*) as a transgene accelerates flowering in the field, indicating GA and DELLA proteins may affect the juvenility of woody plants [57]. The repressors of DELLA proteins, PHOR1, GID1 and GID2, and DELLA domain protein GAI showed decreased expression levels in this study (Table 3). However the GA regulatory pathway in woody plants remains unclear; whether the down-regulation of these genes is associated with the shorter grafting juvenility of ‘Sijimi’ needs further study.

The repressor *TFL1* is thought an important gene regulating juvenility and flowering seasonality in woody plants [18], [58], and is involved in the thermosensory pathway of *Arabidopsis* [59]. One of *TFL1* homologs in longan, unigene 6475, was slightly down-regulated in S according to the transcriptome data, its genomic DNA and cDNA sequences between S and L were compared and no differences were found. If longan *TFL1* (Unigene6475) is involved in the regulation pathway of seasonal flowering then more experimental evidence is required. Another *TFL1* gene in longan, Unigene6027, showed higher expression level in S, suggesting it may have different function in longan.

As plant maturity proceeds, there is a decline in miR156 levels, and an increase in levels of certain SPLs, which leads to the activation of *FT* in leaves. This increase in SPLs in the meristem leads to the activation of *FUL*, *SOC1*, *LFY* and *AP1*, promoting the floral transition [60], [61]. However, the expression levels of SPL3 and SPL9 were observed to decrease in S, so these gene homologues may not be involved in the regulation of seasonal flowering. Alternatively there may be other SPL genes acting in this process.

Combining the results of gene expression levels from RNA sequencing and q-PCR, *SVP*, the integrator and repressor of flowering regulation, was significantly down-regulated in the S sample. *SVP* mediates ambient temperature signaling within the thermosensory pathway, and endogenous signals from autonomous and GA pathways [14]. Several genes in automous pathway were up-regulated in the S sample, therefore *SVP* may be affected by these genes. Recently, *CCA1* and *LHY* were reported to decrease SVP protein stability, possibly through protein–protein interaction via ELF3 [62], [63]. This suggests *SVP* is regulated by circadian clock related genes and may participate in the regulation of seasonal flowering in longan. Furthermore, multiple copies of *SVP* were found in longan. Further research is needed to ascertain which *SVP* gene(s) participate in regulating seasonal flowering.

Plants possess a circadian clock that enables them to coordinate internal biological events with external rhythm changes [10]. In woody plants, the circadian clock may sense seasonal cues. The circadian clock in *Arabidopsis* is composed of at least three interlocking loops to measure day length changes and regulate FKF1, GI and CYCLING DOF FACTOR (CDF) [8], [10]. FKF1 and GI form a complex which degrades CDF proteins under LD conditions, facilitating the expression of CO [33], [64]. Two *GI* and one *FKF1* genes of longan were identified in our sequences, and their expression was significantly up-regulated, especially *FKF1*. However, the expression of the eight *CO-like* genes did not vary significantly between the two cultivars. GIGANTEA (GI)-regulated miR172 defines a unique genetic pathway that regulates photoperiodic flowering by inducing *FT* independently of *CO* in *Arabidopsis* [65] and *GI* also directly activates *FT* [66]. So, in more mature longan tissue, *FKF1*and *GI* may directly work on miR172, *FT* or other floral integrator genes, affecting the flowering seasonality. GI interacts with SVP *in vivo* and controls expression at the *FT* promoter in *Arabidopsis* [66], whether *GI* pathway interacts with the repressor *SVP* in longan needs to be further investigated.

One gene of great interest from this current study is *ELF4* (U4309), which was up-regulated nearly 90 times in ‘Sijimi’ compared with ‘Lidongben’. The *ELF4* gene controls circadian rhythms and flowering time in *Arabidopsis thaliana* [67]. *ELF4*-deficient mutants show an early flowering phenotype with increased CO expression, while over-expression of *ELF4* delays flowering [67], [68]. In ‘Sijimi’, *ELF4* was significantly up-regulated, suggesting it may have important roles in regulating flowering time in longan. *ELF4* and *GI* have a synergistic or additive effect on endogenous clock regulation [34] and temperature signals feed into the clock transcriptional circuitry through the complex repressor including ELF4 [69]. We hypothesized *ELF4* may be the key gene associated with ‘Sijimi’ mutation, interacting with *FKF1* and *GI* to influence flowering time (Figure S3).

In conclusion, this is the first report of gene expression profiling in longan shoots, allowing the study of 539 potential flowering-related unigenes differentially expressed between ‘Sijimi’ and ‘Lidongben’ cultivars. More than 100 putative flowering-time genes were identified and their expression levels between two samples were compared by RPKM method. The analysis reveals the candidate genes related to continuous flowering in longan, which may provide insight into flowering regulation in woody plants.

## Supporting Information

Figure S1 **Flowering-related genes in DGEs GO Functional Enrichment Analysis.** A. GO Molecular; B. Go Cellular; C. GO Biological Process.
(TIF)

Figure S2 **Comparison of Longan** ***TFL1*** **(Unigene6475) genomic DNA sequences of coding region between ‘Sijimi’ and ‘Lidongben’.**
(TIF)

Figure S3 **A hypothesized model for** ***ELF4*** **,** ***GI*** **,** ***FKF1*** **and** ***SVP*** **in the regulation of continuous flowering in ‘Sijimi’ longan.**
(TIF)

Table S1 **Primers for qPCR.**
(DOC)

Table S2 **Pathway enrichment analysis of significantly differentially expressed genes (DEGs) based on the Kyoto Encyclopedia of Genes and Genomes (KEGG).**
(XLS)

Table S3 **Sequences of 107 flowering-time unigenes in longan.**
(DOC)
