# Supplementary figures and images for: Identification of Regulatory Genes Implicated in Continuous Flowering of Longan (Dimocarpus longan L.)
Source: PLoS One. 2014 Dec 5;9(12):e114568. doi: 10.1371/journal.pone.0114568 (PMC4257721; doi:10.1371/journal.pone.0114568)

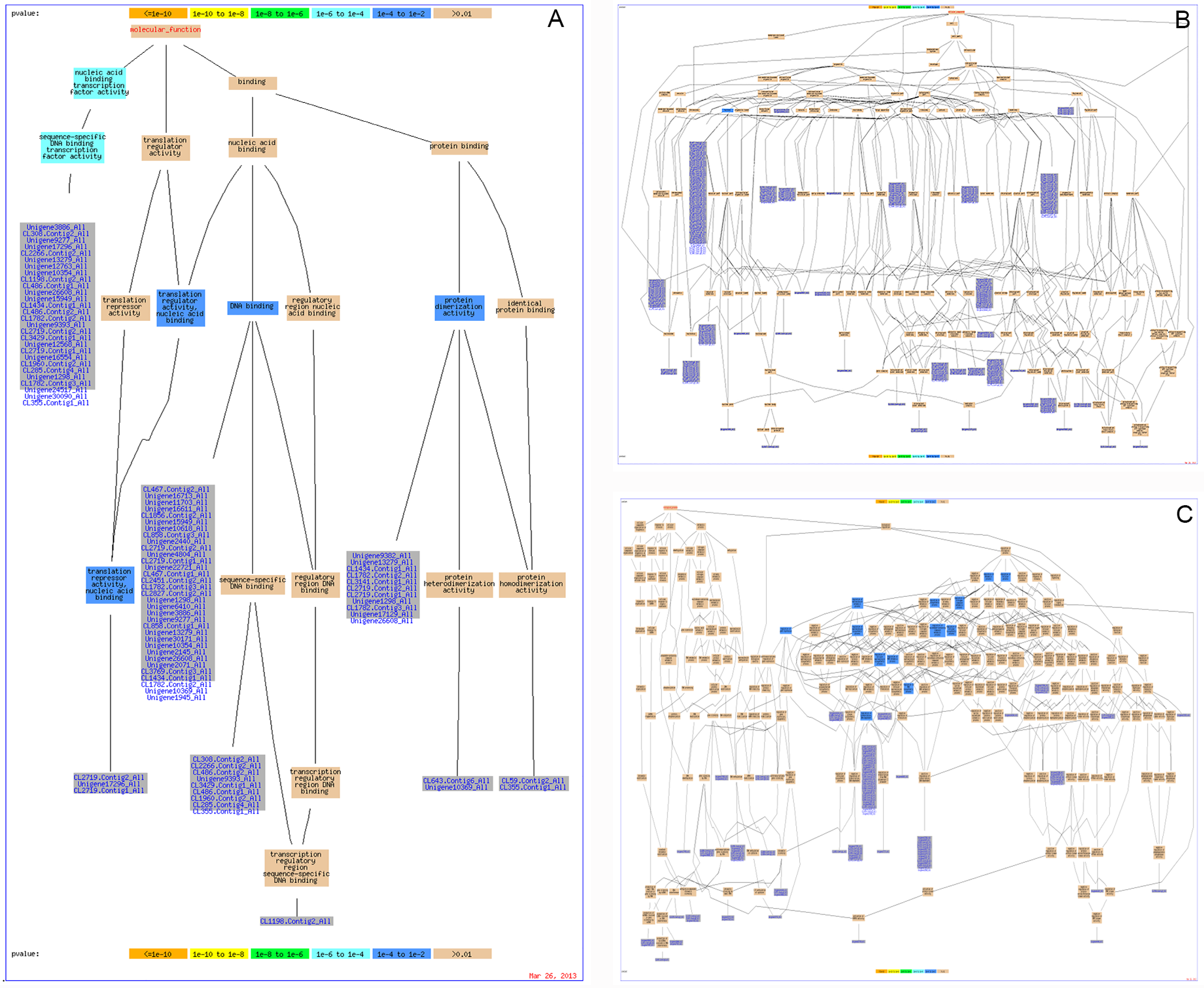

Supplement: Figure S1 — Flowering-related genes in DGEs GO Functional Enrichment Analysis. A. GO Molecular; B. Go Cellular; C. GO Biological Process. (TIF) [file pone.0114568.s001.tif]

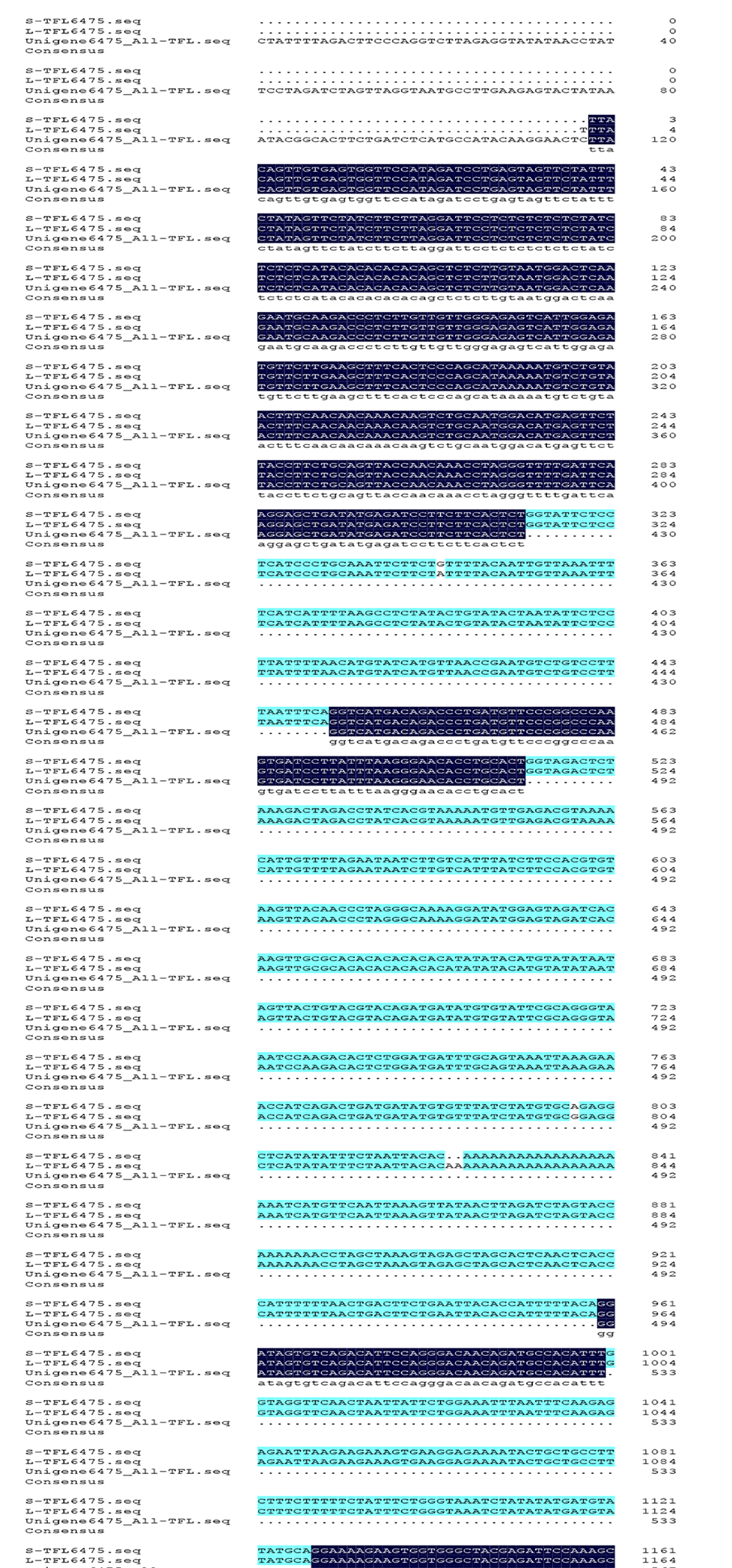

Supplement: Figure S2 — Comparison of Longan TFL1 (Unigene6475) genomic DNA sequences of coding region between ‘Sijimi’ and ‘Lidongben’. (TIF) [file pone.0114568.s002.tif]

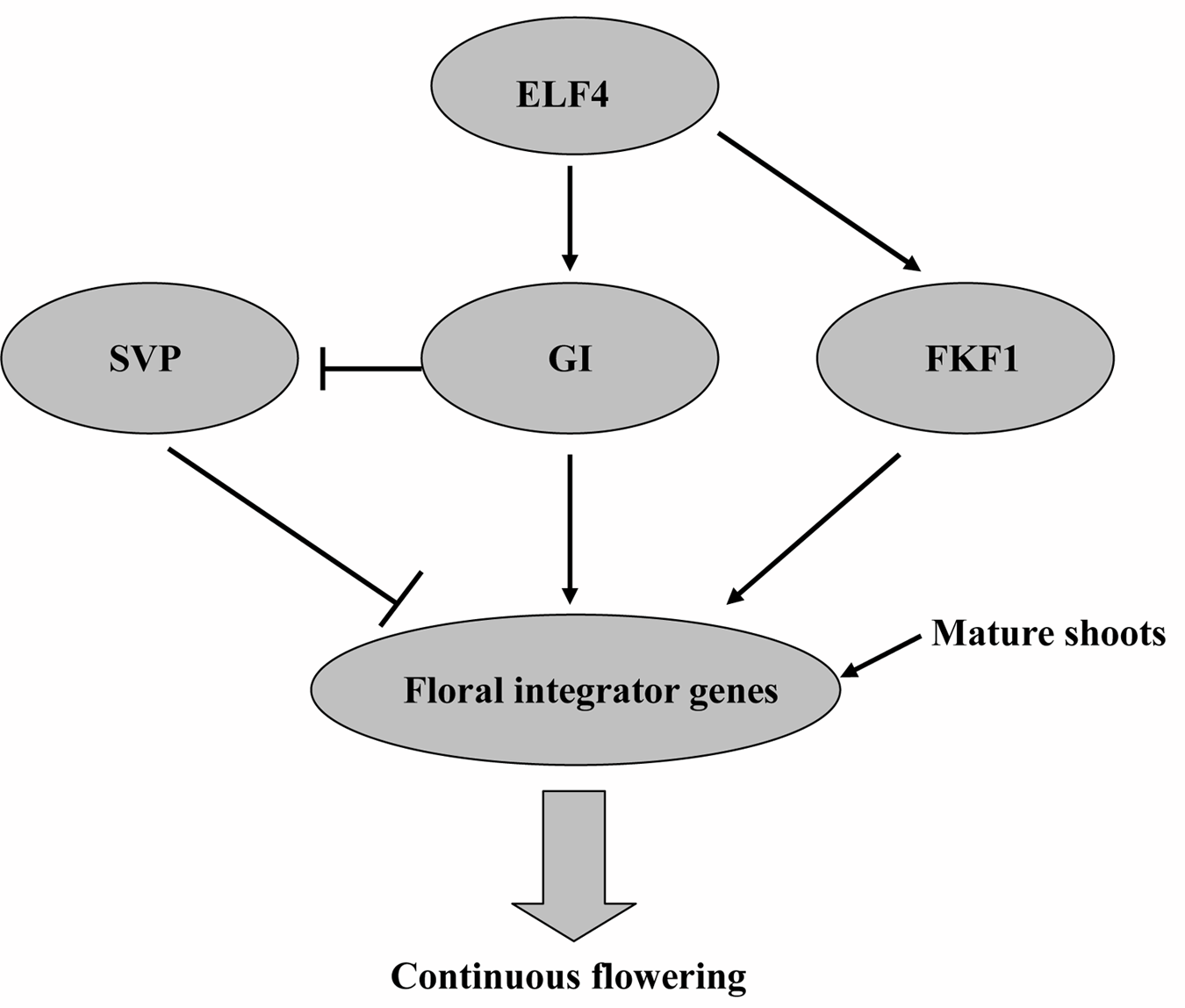

Supplement: Figure S3 — A hypothesized model for ELF4 , GI , FKF1 and SVP in the regulation of continuous flowering in ‘Sijimi’ longan. (TIF) [file pone.0114568.s003.tif]
