# Supplementary material for: Identification of Regulatory Genes Implicated in Continuous Flowering of Longan (Dimocarpus longan L.)
Source: PLoS One. 2014 Dec 5;9(12):e114568. doi: 10.1371/journal.pone.0114568 (PMC4257721; doi:10.1371/journal.pone.0114568)
Supplement: Table S1 — Primers for qPCR. (DOC) [file pone.0114568.s004.doc]

| **Table S1. Primers for qPCR.** | | | | |
| --- | --- | --- | --- | --- |
|  | | | | |
| **Genes** | **Primer** | **Sequence (5' to 3')** | **Product length/bp** | **Annealing tem.(℃)** |
| *ACTIN* | Forward | TTCCGCTGCCCAGAAGTCCTCTT | 155 | 57 |
|  | Reverse | CATTGAACATAGTTGAACCACCACTGAG |  |  |
| *SOC1* (Unigene10736) | Forward | TGACATGCAGAAGACATTGGAACGAT | 111 | 53 |
|  | Reverse | TTCTATATCTTGCTTCACTTGCTCCACG |  |  |
| *SOC1* (Unigene13279) | Forward | GTGTACAACCATGGCGGCTAGGATC | 83 | 58 |
|  | Reverse | GTCTTCATTGCTGGGTGTTTCTGGCT |  |  |
| *AP1* (Unigene15717) | Forward | GAACTCGTCCACTATTCTTTTAAGGCAGC | 246 | 55 |
|  | Reverse | GGGCAAGATTGTATTAGCTCGATTTTG |  |  |
| *LFY* (Unigene14549) | Forward | TGCCGAGATTTCTTGATTCAGGTC | 122 | 55 |
|  | Reverse | GCTTGTTAATGTAGCTCGCTCCTG |  |  |
| *SVP* (CL2719) | Forward | TCAATTGATGGAAGATAATGAACGTT | 126 | 53 |
|  | Reverse | AATCACCAACATGTCTCCGGC |  |  |
| *SVP* (CL645) | Forward | ACCCGTGAGCTAAGGCAGATGAGG | 365 | 56 |
|  | Reverse | ATCTCCTAAGCTGCTGATATTGGTGAC |  |  |
| *FLC* (Unigene8992) | Forward | CCAACTGGAGAAACAACTTGATGCTG | 120 | 56 |
|  | Reverse | TCAATGCTGCAATCTCGCTTTCTAGAC |  |  |
| *FKF1* (Unigene836) | Forward | CCCACCTCGACTTGATCATGTTGCAGT | 169 | 58 |
|  | Reverse | CTTAGGGGGTTGACCAGGAACGTTCAGAAT |  |  |
| *ELF4* (Unigene5963) | Forward | GTGGAGACGTGGAGGTTTGG | 159 | 58 |
|  | Reverse | TGATCTGGCGGATGAGAGAG |  |  |
| *ELF4* (Unigene4309) | Forward | GTCACTCAACTGCGGCTTCAAGG | 230 | 56 |
|  | Reverse | TTGATGGAAAGCAGAGGAGAAGTCAG |  |  |
| *TFL1* (Unigene6027) | Forward | ACTCTCTCTTTCTGATCTCTCTCACATGG | 198 | 55 |
|  | Reverse | GGAAAAAGCTCATGGCCATTATATACTTGC |  |  |
| *TFL1* (Unigene6475) | Forward | CTCATACACACACACAGCTCTCTTGTAAT | 225 | 54 |
|  | Reverse | CATTGCAGACTTGTTTGTTGTTGAAAG |  |  |
| *EMF2* (Unigene6914) | Forward | CGTTGGAATCTCTCTGTGTGATGTGG | 80 | 58 |
|  | Reverse | CGGGACAAGTCACCTCAACTCTCTGT |  |  |
| *GI* (Unigene12571) | Forward | GCAGTGGTGTCATGGGTAAATATGCTG | 230 | 56 |
|  | Reverse | GCCGTTGTTGCAGGAGGAAGTAG |  |  |
| *GI* (Unigene3692) | Forward | GGAGAGGGAATTGCAGCCTTGGAT | 160 | 56 |
|  | Reverse | TGTGGCACGTAAAAGTAGATCTGATGC |  |  |
