# Supplementary material for: Identification of Regulatory Genes Implicated in Continuous Flowering of Longan (Dimocarpus longan L.)
Source: PLoS One. 2014 Dec 5;9(12):e114568. doi: 10.1371/journal.pone.0114568 (PMC4257721; doi:10.1371/journal.pone.0114568)
Supplement: Table S3 — Sequences of 107 flowering-time unigenes in longan. (DOC) [file pone.0114568.s006.doc]

| Table S3.Sequences of 107 flowering-time unigenes in longan. | | | | |
| --- | --- | --- | --- | --- |
|  | | | | |
| GeneID | Size | Gap | % | Sequence |
| CL703.Contig1 | 2649 | 0 | 0% | CACATAAATGAATAGATAAATGATTAGGAGATAAAAGTGTAAAGCCAACA  ACACTTTTTTCATGGCTATGCTCGAGTTTCCTTCCCATAATATTGAAAAG  ACAAGAACCCAACAATCGGAAAGCCAAAACTTATAAGATTAACTACAAAA  CTCACAGGTTTTGCTTCAACAGAAAAAGAAAGAGAGAGTCATGGAAAATA  AGTGAGAGACATAAACTAGTGGGTGTGTGTGTTTTGGATATTTGGTTGGT  AGAGTTTTTTGTTTTTTTGGGAGATGTCAGGTGGTGGTTGTAGCATAGTT  TGGTTCAGGAGAGATCTAAGAGTGGAAGATAATCCAGCACTGGCGGCTGG  TGTGAGAGCTGGTGCTGTGGTTGCCGTCTTCATCTGGGCACCTGAAGAAG  AGGGTCTTTTCTATCCAGGGAGGGTGTCTAGGTGGTGGCTCAAGCACAGC  TTGGCTCATCTTGACTCTTCTTTGAGAAGTCTTGGTACTTGTCTCATCAC  CAAGAGATCCACTGACAGTCTCTCCTCTCTTCTTCAAGTTGTTAAAGCTA  CTGGTGCTACGCAGCTCTTCTTCAACCACTTATATGACCCCTTGTCACTG  GTTAGAGATCACCGAGCAAAGGAGGTTTTAACTGCTCAAGGTGTAGCAGT  GCATTCGTTTAATGCAGATTTGCTTTATGAACCATGGGATGTAAATGACG  CGCAAGGCCGTCCTTTCACAACCTTTGCTGCTTTCTGGGAGAGATGCCTT  AGCATGTCTTATGATCCTGATGCTCCACTTCTTCCACCTAAGAGGATAAT  TTCAGGTGATGTATCTAGGTGCCCTTCAGATACTTTAGTGTTTGAAGATG  AATCAGAGAAGGGAAGCAATGCACTTCTTGCTAGAGCATGGTCACCTGGA  TGGAGCAGTGCTGATAAAACTCTGACCACATTCATTAACGGGCCTTTAAT  CGACTACTCTAAAAATCGTAGAAAGGCTGATAGTGCTACAACCTCTTTTC  TGTCTCCACACTTGCATTTTGGGGAGGTGAGCGTGAGAAAAGTCTTCCAT  CTTGCCCGCATTAAGCAGGTTTCATGGGCCAATGAAGGGAACAAGGCTGG  TGACGAAAGTGTGAACTTGTTTCTTAAGTCAATTGGTCTTCGGGAATATT  CAAGATACTTGAGCTTTAACCATCCATACAGTCATGAAAGGCCTCTGCTT  GGGCACCTGAAATTCTTTCCTTTGGGTTGTGGTTGAGGGCTATTTTAAGG  CTTGGAGACAAGGTAGAACTGGCTATCCATTGGTGGATGCTGGCATGAGA  GAGTTGTGGGCCACTGGTTGGCTTCATGATCGGATACGAGTAGTAGTTTC  TAGTTTTTTCGTAAAGGTTCTACAGCTTCCGTGGAGATGGGGAATGAAGT  ATTTCTGGGATACCCTCTTGGATGCAGATTTAGAGAGTGATGCTCTTGGT  TGGCAGTACATTTCTGGTACTCTTCCTGACGGCCGTGAGTTTGACCGGAT  AGACAATCCACAGTTTGAGGGTTACAAATTTGACACCAATGGTGAATATG  TACGACGCTGGCTTCCTGAACTTGCCAGACTACCAACTGAATGGATACAC  CATCCATGGAATGCACCAGAGTCTGTACTTCAAGCTGCTGGCATTGAGCT  TGGGTCTAATTACCCTCTGCCCATTGTAGGGATAGATGCAGCAAAAGTCA  GGTTGCAAGAAGCACTTTCAGAAATGTGGCAGCAAGAGGCAGCTTCAAGA  GCTGCAATTGAGAATGGAACTGAGGAAGGTCTTGGAGATTCCTTTGAGTT  AGCCCCAATTGCCTTTCCTGAAGACATACAAATGGAGGAAAACCACGAAC  CTGCTAGGAATATCCCCCCTACCACAACTCGGTGTTATGAGGATCAGATG  GTTCCAAGCATGACTTCTTCTCTCATGAGAGTTGAACAGGAAGAGTCTTC  CCTGGATCTTCGAAATCCAACAGAAGATAGCAGAGCAGAAGTACCAAGAA  ATGTTAATGTAAATGTAAATCAAGAACCTAGAACGGACACATTAAACCAA  GGGGTTTTGCAAACAGTTCATAACAACACTTTGCCACAAATTAATGTCAT  AATAGGGCTGGCAAATGATGAAGACTCCACGGCAGAATCTTCAAGCACCA  GTAGAAGAGAGAGAGATGGAGGAGTGGTTCCAGTTTGGTCTCCACCAACT  TCCAGTTACTCTGAGCAGTTAGTGGGTGACGAAAATGGCATTGGAACAAG  TTCATCATACTTGCAAAGGCATCCACAGTCTCACCAAATAATAAATTGGA  GGCGACTTTCTCAGACTGGGTAAAACATATGTACATGACACAGACCAGAA  GCTCAGAAGCTGGGGGATGCGATGATGCAGAGGGATCGTAAATTAGTGTC  ACACATTTATCTTTATTGCTAGTCTAAAGTCTAAACTGATAAGCAGGCCC  CCTTCTATGTGCGTGAACTTTCAAATTAGTCCAGGCAGCCATCGGCATCC  GATTTCCTTTTTTCTTGTACCATATACACAGCTCTACTGTATAGTATTTG  TTGTTTTCATAAGCGAGAGAAGAGTCTTGATAAATGGTAGGTATGTGTTG  TCCTCTAATAAACCCAAGTTTGTGCTGCTTCAGTTAATTGATTAGGCAG |
| CL903.Contig2 | 1333 | 0 | 0% | GTCTGCATCTAAAAGTGTGTCCCAGAAATACTTCATCCCCCATCTCCATG  GAAGAAGCAGGAACTTAACAGCAAAACTTGAAACAATAACTCTTATTCTG  TTGTGTATCCAACCTGTAGCCCAAAGCTCTCTCATTCCAGCATCTACCAA  AGGATACCCAGTCCGACCCTGTCTCCAAACCTTAAACTGGTTCAAGTCAG  AATGCCATGGAAAATATTTTAAGTTGCTCAGCAATGACCTCTCATGAGTG  AATGGAAAGTTGAAACAAAGATAACGGGAGTATTCTCTAAGCCCAATGGC  TCTAAGAAAAACAGTCACACTATCTTTCCCTTCAACGTTTTCTTCTTTTG  CCCATAATATTTGCTTCATGCACACACATTGGAAAACTTTCCTCACGCTT  AGCTCTCCAAAATGAAGATAAGGAGACAAAAGTGAAGTTGAGTTCTCACC  AAGCTTCTGTCTATTTTTTGAATAATGAAGGAGGTGGTGTTCAACAAATT  CTGTCAGAACCTTATCAGCATTGTTCCAACCTGGGGACCATGCTCTTCCT  AATAAGGCATTACTAGATTTTTCGGATTCATCTTCAAGACCTAGTTCCTC  GATAGAGCATTGCTCAACTTTGCCTTCAGCAGGCACCAAACGCCACGGAG  GAAGAAGCGAAACAGGTTCCATTGGCATTTCCAAACAATTGTTCCAGTAT  GCATCAAACATGGTAAAAGCATATCCACCTTCATCGTATATTTCCCATGG  CTCATACAATAAATCTCCATTATAGCTTTGTACTGAAAAGCCAAGCTCCA  CAAGTTTTTCTTTGATGTTGTGATCACGAACAAGTGAGACAGGATCATAA  AGATGGTTGAACACTACTTTTGTCGCTTTAACAGCAATGATGCACTGCAA  AAGAGCATCAAGAGTACTCTGAGTTTTGATCAACACAAGGTCAGCTCCCA  GAGACTTTAGGGATTGTCTCAACTGAGAAAGCGACTGCTTTAGCCACCAC  CTCGACACTCGACCCGGGTAAAACTGTCCTTCTTCTTTAGGGCACCAAAT  ATAGACAGGAAAGACACAGCCATCCTTTGCAGCAGCAGCTAATGCAGGAT  TGTCCTCAATCCTAAGGTCCCTTCTGAACCATACTATGGTCCTATTGCTG  CTACCCATACTGTTTAACCTAGATCTTTCAAAGACAAAACAAAACAATAA  GACTCTGAATAATTTCACTTTGTGTTTTTTCAATCCAGAACTAAGAACCT  TTCTTGGTTCAAGATCTATCAGACAACGTCTAGAGTTCACTTCCAGATCA  ACCCAAAGTTGAACTATATGACCACAATAATTC |
| Unigene1512 | 320 | 0 | 0% | CATCTTGAATATAGCTCCCGTCAAACGTTCTCGGGCCAAGTCAATATCAA  TAATAGGTTTTGGATAGTTTAATCCCAACTCCACCCCAGCAGCTTTAAGA  ACGGTAAGAGGTGCATCCCAAGGATGATGAATCCACTCAGTTGGCATTCT  TGCTAGCTCAGGAAGCCACTGTCTCACATACTCGCCCTCCGGGTCAAATT  TGGACCCTTGAATCTCAGGGCTGTCCATTCTTGCAAGTTCATGGCCATCT  GGTAAGCTTCCAGAAATATACTGCCAACCAAGGATATCGCTTTCCAAGTC  TGCATCTAAAAGTGTGTCCC |
| Unigene6715 | 361 | 0 | 0% | AGAGAAGCTGGAATTTTGATGCAGCCGCGTACTTTGGACTTAGCTTCAAC  AGACTTGCGAAATTGAGTGTACACATCTGGTATAGAGCTGGAAGTGAACG  GAATGTCATCTAAATGGTACATGGTTGTGCCCCAAATCAGTTGTAGCTTA  GGACTTTTCGAAGAATTCTTGTTCTCGAATTCATCTTTAGATAAGGATGA  TGATGACCATGATGACAACACCAGTTTCCCCAATCTTTTCCTAACCAAAT  TTTCCACGTTAACCTCCTCACTACAGGTTTCTTTTTGGGCATACACTGAA  TGTGCACCAAAAGATTTTGCAAGAGAAGGGATAATTTCCTCAGGTTTCCC  GTGCTGAATCA |
| Unigene7391 | 1094 | 0 | 0% | TTCTCCAATTTATTTTCAACGCAAAATGTGGGACTCTAGGCATTTGAATT  GTCAAATGAATAGAATCATGAACCGTCTCAAATTTTAATAAGCATGTATT  ATACATTGTTAAGTATAATCTCTCAGCCATTAGTTACTTTTTAGGTCATC  ATGGTCTCTGTTGTGTAGCTTGTTTAGCTCTGAAATTCCTCCGTCTCCCA  GTCGAGGCCTTGTCCTGATTTCTGTTTCGATTCACGTTTCCAAATTTGAG  AGGTACAACTTGCCCGATGTATGATTTTCCAGGAAAGTTTCTCTTGTCTT  TAGCAAGTGCTTGTAGCTGTGGCAACCAGTATGCCACATACTCTCCTTCA  GGATCATATGTTTGTGCTTGCTTGGGGATGCTGAAATAACGATCTTCTCT  TGGATCATTACCAACGCCTGCTCCATAATTCCAGTTTCCATAATTGGAAC  ACGGATCATAATCTAACAGGCATGTTTCGAACCATTCGGCGCCCATACGC  CAATCAATGCCCATATCTCGGACAAGAAACGAACAAACAATCTGCCGTCC  TCTGTTTGACATGAACCCAGTTGTCGCAAGTTCCTTCATGTTGGCATCTA  TGAGAGGGTACCCCGTACAGCCATCTCTCCAGGATTCAAACAATTTCTGG  TCTTGACTCCATTTATACTCTACTTTTCTGGGGCCACCTAAATGAAAAAG  GGCATTTCCATGTTTGAGTGAGATAAATCTGAAGTAGTCCCTCCATATCA  GTTCAAACAAAACCCAATATGTTGAATCATTTGCTTGCCTTTCACTTTCA  TATCTCTTTACCTCTTCGTGTATGTAACGTGGAGAGAGGCTTCCTGAGGC  AAGCCACGGAGAGAATTTTGTTGAATAATCAGGCCCCAACATCCCATTCC  TTGTCTCTTTATACACCCTTAGAAGATCCTTCTTCCAAAAGTACTCATAT  GCTCTGCCAAGAGCCGCACTTTCACCTCCCACAAACTTCATTCCTTTACT  AACCTTTTGTGATTGAAGTCCAAGTTGATTGATGTAAGGAACACTTCCCC  AGTCATCAACACTAGGTGCTGGTCCAAGAGAAGCTGGAATTTTG |
| Unigene8667 | 2200 | 0 | 0% | GTGGAAAGGATGCAATTAGGAAAAGATGACAAAAAGCTAGCACGATTGAG  CAAGCGTAAAGCTGTTCGTAAAGACTAATGATACAACATTATCTACATTT  TGGGACAACAGAAATAGGCCAAAAGGAAACCACACGGTTGGCAAAATATG  TCATTGAACTTCAAATCCTCTATTGGTAGGAAACACGTTACTGATTAGAA  CTTACTTCCTCAAAAATTAATTAGTCCTGTGACTTATGGGCTGCAGCAAG  CTCAACTGTTACGATGAAAGTTGACTTACCTGCTTCCCTCAGATACTGTA  CATCTCCGTTCATTAGCTTTACCAGTTTTCTGCTGATTAGCAGGCTAATG  CCCTCCTCAGTAGCATCTCCATCAGCTCCAAACATTTGGTCCAGCAATGC  TTCAGGTATCCCACCACCAGCATGTGTTATCCTGAGCTCCAGACGAGCAA  GATGAACAGATTGGCCCAACTGATCTTTGGTCAAACTAGTTGAAACAATA  AGCTGGCCTCCATTTGGGGTAAAACTAACTGATACTGACAAGAAATCAGC  TAAGACCTGTTGAAGCCTAATGCTATCTCCATACAATGTTTCATTCATGA  TCTCTTCTGCTGTTTCATTGACCACACGGATGCCCTTTCCATTGCTCTTC  ATCATGACTTGACTGATAGAGGCAACCAACACGTCATGCAGAGTAAACTC  GACCATTTCGAGATCCAAGTAGCCATCAATGATGCTATCAAGGTCCGAGT  CATCAAGAATCTTATTAAGCTGGCGCTGGCATTGTGCACTGGTATGCAAA  AGCTGTTTCTGTTCTACTCCTAGCTCAGTGCCCTCCAACATTTTCCGAGA  AAATATAATACCAGAGAGAGGATTCCTTATCTGCCGTTTGATATAAGCCA  GTGCTTTCAATCTCTTGACGGCAGTTTGCTCGGACAATCGCTGGATATGA  AGAGCCTGTTGCAATTCATGGCTGGCAAGCTGCAAGAAGCAGAAGACACC  AGTGATTGCTCCCTCTCCGTCCAATTTCTTACTCAAGCACAACAAGCAGT  CCACGTACTTTCCATTCCGGGCAAAGAAACCAAAGGGAACCTTTTCAGGA  TCCTGACCAGACATGGCATTATTTAGTACAATGCCAAGATTTACAAAACA  CTCTTGATTCTTGAGCCGACAGCATGCCATATTGGTACCAAAAACCTCTG  CTAAAAGCAATTTGTCCACTACTTCTTCTCGTTTCCACCCAGTTAATTTT  GCCATTGCCGGATTCCACTCTGAGCACCATCCAAACTCATCCACACCAAA  TATGGGGGGTATCAATGGGTTGGGATTTTGCACAATTGCTTTGTAATCAC  CTTCAATCCGGGTGAATTTGTCCATGACTGTCTTCTGACCAGTGATATCT  TGTGCCACAAAACAAACTCCAACAACATTTTCATGAAGGTCTCTGCTTGC  ACAGGCATTCACTATTAAACTGATGGGGCCAGCATCACTCCTACACCCAT  GCGTTTTGATCTCAAATTCGACGTCCTTCTCCTCATGGCCCTGCAATGCC  AAGTGTAACATCTTTCTGACTATGTCAACTGAAGAATCTTCCACAAGTGA  GAGGAAATGCTTTCCTATTGCTTTATCAACAGGAAGACCAGTTAATTCAG  CTATTTTTGTATTCCATCCATTAACCAGCCCATCGACATCGACTGCCAGA  ATTGGTACTGTAGCTGTTTCAATAAGGCGGACCATCTCACTTGTCACTGC  TTCAAGCTCTTTCATCCCTTCTAACTTGAGGTCATTGAGCTTCGAATGGA  TTGATTTGTTGTTTACATCCTCAGTTTCCACATCCTTGAATGCATTTCTC  AATATAAGCTGCAAAGAATGGATTGCATCCATTTCATAGTCCTTCCAAGG  CAAGCTCCTTGTCTTAACAACTTCAAGGAAAGCCTTGAACGATGATCTTG  GGTGCATCTTCCTACCATCATCCTTCTCACCAGGTTCGTGCTTTGCACCA  CCCCATCGAATTGCAGAGGCAGTGTGGGACCGGAACCAGAAAATTACGTC  CTTGGAACTAATCCTCACAGCTGCCATTCCACAGACTACATCACCAAGAG  CCAGTGCCCCTGGGAAACCTGCATCATACAAGCTATCCGTACTCAAACCT  GTTGAATCCATATGGTATTCAAAAAGCCATGAGGCTATATCTTGTAGCTG |
| Unigene1734 | 2472 | 0 | 0% | ACTTGGTAATGCAGTTTGTGGGATGGCAGTTGCTTATATTACTAAAAGGG  ATTTTCTATTCTGGTTCAGGTCCCATACCGCAAAAGAGATAAAATGGGGT  GGTGCAAAGCATCACCCCGAGGACAAGGACGATGGGCAGAGGATGCACC  CACGTTCTTCGTTCAAGGCTTTTCTGGAAGTAGTGAAGTGCCGGAGTTTGC  CATGGGAGACTGCAGAAATGGATGCAATCCACTCTTTGCAGCTGATTCTG  CGAGATTCGTTCAAAGATACTCAAGCCAGCAATTCTAAGGCTGTTGTACA  GGCAGAGGTTGGGGATCTAGAGTTGCAAGGGGTGGATGAACTCAGCTCGG  TTGCCAGGGAAATGGTGAGGTTGATAGAGACTGCAACTGCTCCCATATTT  GCTGTTGATGTTCAAGGTTGCATTAATGGGTGGAATGCCAAGGTTGCAGA  GTTGACTGGACTTTCGGTTGAGGAAGCTATGGGGAAATCCTTGGTTCACG  ATCTTATTTTTAAGGAATATGAAGAAACCGTTGACAATCTTCTTCACCAT  GCTTTAAGAGGTGAGGAAGACAAGAATGTAGAGATAAAATTGAGGACATT  TGGCGCAGAAAACCATAAGAAGGCTGTCTTTGTGGTGGTCAATGCTTGTT  CTAGCAAGGATTATACAAATAATATTGTTGGGGTATGCTTTGTCGGTCAG  GATATTACAGGTCAGAAAGTAGTGATGGACAAATTCATTCACATACAAGG  TGATTACAAGGCTATCGTTCACAGTCCCAATCCTTTGATACCTCCCATAT  TTGCTTCGGATGAAAGCACATGTTGCTTGGAGTGGAACACTGCCATGGAG  AAGCTCACTGGGTGGTCCCGAGGGGAAATCATTGGAAAGATGATAGTTGG  GGAGGTTTTTGGCAGTTGCTGTCGGCTCAAGGGTCCTGATGCTTTGACAA  AATTCATGATTGTCTTGCACAATGCATTTGGAGGACAGGATACAGAAAAG  TTTCCGTTTTCCTTTTTTGACCGGAATGGGAAATATGTGCAAGCACTGTT  GACGGCCAACAAAAGGGTAAATATGGATGGTCAGATTGTAGGAGCTTTCT  GCTTTTTGCAAATTGCAAGTCATGAGTTGCAGCAAGCTCTGAAAGTCCAG  CGGCAGCAGGAAAAAAGTTGCTTTGCAAGGTTGAAAGAGTTAGCTTACAT  TTGCCAGGAAATAAAGAATCCACTGAGCGGTATACGATTTACTAACTCAT  TGTTGGAGGCTACTGACTTGACTGAAGATCAGAAACAGTTTCTTGAGACT  AGTGCTGCTTGTGAGAAGCAAATGATGAAGATCATCAAGGACGTTGACCT  GGAAAGCATTGAAGATGGTTCATTGGAGCTTGAGAAGGCTGAATTCTTTC  TCGGGAGTGTGATAAATGCGGTTGTTAGCCAAGTAATGATGTTGCTGAGA  GAAAGAAATCTACAATTGATTCGAGATATTCCAGAGGAAATCAAGAGTCT  AGCTGTTTTTGGTGATCAAGCAAGAATTCAACAAGTCTTGGCCGATTTCT  TACTCAATATGGTGCGTTACGCACCCACAACAGAAGGATGGGTAGAGATT  CATGTTCGTCCAACAGTGAAGCAAATCTCTGACGGACTAACTGTTGTGCA  TACTGAATTCAGGATGGTATGCCCTGGTGAAGGTCTTCCTCCTGAGCTAG  TTCAAGACATGTTTCATGGAAGTCGATGGGTGACTCAGGAAGGCCTAGGA  CTGAGCATGTGCAGGATGATTCTGAAGCTAATGAACGGTGAAGTCCAATA  CATCAGAGAGTCAGAAAGATGTTATTTCTTAATCATCCTTGAACTCCCTA  TGCCTCAGAGAGGCTCAAAGAGTGTTGTTGACTAGATTTTTCCCTCCTCA  AATTTAACCTGAAGATACTTACTGTTGCATATACTTCTGTGTATCTTTCG  TATAACTCACTTACATGGCTTACACTCACACCTCTGCAACCTGATTCGCC  TAATCGGTGTTAATAAATGAATCTTGAGTTACTCCTGGAAAGTTCTTCAG  TCTTATACCATCTGCTGGTTCCAGTCCACATCAAGGCTTATTGAGCTACA  AATCTTCTATCTACCTGAGTTGATATCTAAAATGTCAAGTTGTGATGCTT  CCAGGTCTTTATGCAACATGTGTGCTGCCATATATATGTGAGGTCACTTG  AGATTGTTGTAGATGTTTCCATAATGAAAAGTCATTGCAGACTAGCGGTT  GGATGGAATCGCAGCTTCCCCGGCTGTAAGCATGTATTTTAGGCCTGCTG  TATGTATTATATCAAATTCTCACGATGAAGTGTAACATATTGTCCAGGGT  TACTCCCAGATTGTTATTATTCTAAATCTCTTAGCTAGTATTTTATCTCT  ATGTCGGTGTAATAAAATCAGTTTCTCTTGCTCTTGTTGTTGATGTTCAT  GTAACACCTGTTTAGTCTACAG |
| Unigene14185 | 1234 | 0 | 0% | CTTTGAATGATTGTGGATTGTAGAAACATTTAATATACATAATGATGATT  TGATTCATAGCCAGAAAAGGGTTGCATCCTGGCCTGCTCAATCAAAATAT  GTTAAAACTATACAGTTTGCCATGTTGAAACACTTGCTCAAAATGCATGG  AGAACAAACAGTTGCTATCTTCTTCATCAGAAACTGAAACTATATAACTG  GAAAAATGGTGTTACATGAAACTGAATATACTATGTCTGGCTAGTAGGGG  GAGCTGCGAGAATTTCCACTGAACATATTCTGATGCTCAACGTTTCAGGC  ATGAGCTGTGTACGATTCTTGATTCAAACCTGAGGATGACATGCCGCTTA  CTTGAAATTTTATGTGACTCAGTTGAATGGACCGTATCTCTGCTCGTTAA  ATTCAAAAGAGTGTTTTTGTGTTTTGTCAGTAGTCAGTTTTCACGAGCCA  ATGGGAATTCAATGAGAATAAGGAAGGATGATTTTTCAGCCTCTCTCACG  TACTGCACGGTGCCATTCATGATCTTAACAAGCTTCTGGCAGATATAGAG  GCCAAGACCTTCCCTTGAGGCACCCTGGCTGTGGTAGAACATCTCATGGA  TTAACTTTTCTGGTATTCCAGGTGCTGGATGGGTGATCCGAAATTCAAGA  TGAACAATGTGTATATTTGTCCCTATACGTTCCTTTCGTGGGATTACTCT  GAATGTAACTGAGGATCCCTCGTAAGCAGTGGTGAAGATGAGCGCGTTAA  TCAAAAAATCTGATAGGACTTGCTGAAGCCTCAACTTGTCTCCATACAAG  TTCATGGATGACACTTCCTTCGGTAAATCACGGATGAGTTGCACTTGGTG  TTCCTTGCTCGGTATCATAGCTTGTGTTATCACAGCCTCAAGAGCTTCCG  CAAGGTTGAACTCACTAGATTTCATTTCCATATAGCATTCCTCTATACTT  TCAATGTCGGTGTCATCAACGATCTTGGTTAATTGTTCTTGACACGATAG  GCTTGTTTTCAGTAGCTTCTTCTGCTCTGCGGTTAAATCAGAAGACCCCA  TTAGGTCCCATGTAAAAGCTATCCCACCTAAAGGCTTTTTGATTTCATGA  CGAATATATGCTAACTTATTGAGGCTATTGGCTGCAGCCTGTTCTGATAT  TCTCTGTATTTGGAGAGCGTATTGAAGTTCTGGACTAGCCACATGCAAAA  AGCACAAGGCCCCAGTAATCTTTCCCTCCACATC |
| Unigene8582 | 639 | 0 | 0% | AAGGCCCCAGTAATCTTTCCCTCCGCATCAGTCCTTTTGTTAGCGGAAAG  CAATGCTTCTATGTATTTACCGTGCTGATCAAAGAACCCAAATAATAACT  TATCTGCATCCTGCCCTGCAATCACTCGGTTCAATACTATCCTCAGTTTG  GTTAAGGTGTCAACGTTTTTAACCTGACAACCAAAACTATCGATAGTAAA  AACCTCCCCAAGAAGCATCCGATCAGTGGCTTCTTCCTTCTTCAAACCAG  ACAATTTTTCCATTGCATCGTTCCACTCCAAGCATCGGCCATGCTCATCA  GTCATAAAAATTGGAGGAATAAGTGCAGATGGGCTTCGCACGATACCTAC  ATAATCACCTTGGATACGGGTATATTTATCTGTAATCAACTTCTGCCCTG  TAATATCTTGGCCAACAAAGCAAACACCGACAACATTTTCCTTCGTGTCT  TTACTACAACAAGCATTGACTACTAGGATCACTGGACCACTGTTTTCCTG  AGGACCAAATGTCTTAAGCTTGATTTCAACGTTTCTTTCTTCTATACCTA  GCAAAGCCAATGATAACATCTTCTTGACCACGTCGACTGAATTATCATCA  ACAAGGTCGACAAAGGGTGTGCCAATGGCTTCCTCAACA |
| Unigene12755 | 310 | 0 | 0% | TGAACAATATCAAAGGGGTGTGTTTTGTGGGTCAAGACATCACACCTGAG  AAACTTGTTATGGATAAATTCATCCGCTTGCAAGGGGACTATGAGGCCAT  TATACAAAGTCTTAATCCATTGATTCCACCAATATTTGCATCTGATGAGA  ATGCTTGCTGTTCTGAATGGAATGCAGCCATGGAGAAGCTAACTGGTTGG  ATGAGACATGAAGTGATAGGTAGAATGCTTGTTGGGGAGATCTTCGGGGG  TTTTTGTCGGCTGAGAGGTCAAAATATACTCACTAAATTAATGATTCTGT  TGTATCAGGG |
| Unigene27248 | 250 | 0 | 0% | TTCCCTCTGAAGCCCAAAAATTTTCAGTTTTAGCTCAACATTTTTGTCCT  CCTCACCAAGCAAAGCTCTGCATATGATTCTTTCAACAGCTCTACGTGAG  TCCTCATGAACGATTTCATCAACCAGAGATTTTCCCATTGCTACACTAGC  TTGCAGTCCTGTCAATTCGGCAACTTTTGTATTCCACCCATCAATGTGAC  CAGATGAATCAACCCCAAAAATTGGTGCCGTTGCTGTCTCAATCAGTCTG |
| Unigene2802 | 797 | 0 | 0% | AGGAAGGCGGTGTGGAGCTAAACATGGAAGAATTCCTTCTTGGCAATATC  TTAGATGCTATCATTAGTCAAGTAATGATCTTGTTGACGGACAGAAGTTT  ACAGCTTTTTCATGAGATTCCTGAGGAAGTGAAAACACTCTTGGTTTATG  GTGATCAAGCTAGGCTTCAGCTGGTTTTGTCAGATTTTTTGCTTAATGTG  GTCCATCATGCACCTTCTCCACATGGCTGGGTAGAAATCAAGGTTTTGCT  GGGTCTGAAGATAATAAAGAACAACAATGAATTTATTCGTGTCCAGTTCA  GAATGACACACCCAGGTCAAGGTCTTCCTCCAGCTCTTATTAAAGATATG  TTTGAGAGTGGAAATCGGTGGATTACACAGGAGGGCCTTGGATTAAGCCT  ATCACGGAAGCTTCTCCTTATAATGAGTGGCCATGTCCATTATATTAGAG  AGCATAATAAATGTTATTTCCTCATTGATCTTGAACTCAAAACCAAAACA  AGAAGACAACAGGATTCGCAAGCAGAAACGGGCAGAATGTCCTAATTTAT  TCATCCATCTACTGTCTTCGTTTTGATCACCAGGGTGAAGTAGATTCTGT  TATCATGCAATGATATGGCTGGAGAAAATCATAGCAGTGTACGTTGAACC  TTAACTGTCATTGTGGCTTATGTTCTTTGTCACACTACCGCGTCCTGTAT  TAAGTTACTCCCTGTGACACTAGTCCTAGCATGCTTCTACTTTACTATTT  CAGCCATTTTGTGATGGTATATTTACAACATCAGTACATCACATATG |
| Unigene11739 | 594 | 0 | 0% | AACTGTATCCCAATAATGTGTGTTATTGTCTCATCATCTCCATATATAGG  AGTCAGCCGCAATCTGTTCATCAGAGGAGATCCATCTTTCCGAAAGTTCA  ACAAATCGCCCTGGAATTCAATACCCTCCTCTAGACATCTCCTAATTTCT  GAAACTACTGTGGAATCCACTAATGGATGTCTTCTCTTTGCAAAAGGACC  TCTGCATTGCAAGAACCGGCAATTGCGACCGAGTACTTCCTCAGCGCGAT  AGCCAGTGACCATCTCGAAGACGGTGTTGACATAAATAATGGGATGGTCG  GGCTCAAGCGCGTCCGTCACGATGAACCCACAAGGTGCCGTCTGCAGCAG  ATTCTCCACCGGAAAAGGGAGGGGACTGCCGCTGCCACCATCCTCCACCT  CTTCGTCGCCGCTGAGATCGGAATTGCTATCCCACTCCATTCGCAGCTTT  CAGAATCAAACGAGGAAAAGAGAACTCCCTCCCAACAAGTTCAACCCATA  CTTAGACGAAAAGCCCTATCTGAGAGGTGAAAAGGAGGAAACGCAACGTGC  TGCCTTTGTGTCTGTCCGGCTCTCTTTGGCGAAACTAAAAACG |
| Unigene9169 | 1986 | 0 | 0% | ACGCCACGCCACTATTCTCTTATTCATCTAAAGCAGAATTATCATCTAGA  GTTAAAAGAAAATTAGGATCCTTCACCAGAATCACCACTACAACATTACA  ACATTTAGAAACCTTAACAAAAATACTATAAACATGCAGCAGCCTTTTTT  TTTACCCTTTCCTTTCTTCATGTTTTGTCTAAATTACAGATGCTGAATGA  ATGCAACAGAGTAACATAACAAGTAAAACCTACATATATAAAATACCTAT  TTGTCTAACAAATACAAGCTAACCTAACAAGAAAGTCCACTGTAAGCTGA  GAATGAGAAGAGCAGTGATGATGAGCGGGCAGTTTTACACAAATCTTACA  AAAAACAAACATGGTTTCGAGTCACTCATTGAAATATTCATCCCGAGCCA  GAACAATCTCAATCTCAATCTTGTACACGAGAAAATCCATTACAAATGTC  CTGATTGGACCAATATTTTGTCAAGTGCTTTTGCTGACTGGCCACAACAT  TCTGTACTGAATGAACTCTCACCATTGATATTTTCCATAGAGCTGTTTCC  TATTTTTCACAGTAGATGGTATTTAATTTTTTTCTCAATCAGATGGCAGA  ACTTGCCAATGAGAGCTCATGAAGCTCACTTAGCATCCATTCCTCTCCAG  TTTGGCCACCAAGGACTATAGCCCTTGTTCCCCCTACAACACAAGTACTA  TGTCCCCATGCAAATCTGGGAGGCCGCCCAGGTACATTTAGTATCCTCCA  TGTAGGCTTTTCATCAGTTGGGTCTAGGAGATACAGCTGGGATGCAGAGT  GAAGACCAGCCACAGACCCACCAAAGATCAGAATTCTCCCACCTGGGAGG  CTCACAGCCACATGATCAAGTCTAGGGGGAGGTGCAATGCCCCCTGGGTT  TCCTGCACCAGGCATTCCACTTCCGGTTACACATCTCCAACAAGGTTCCT  CCTCGCTTAAATCCATTGTGAAGACATCACTAGAACGAAACCGTAGGGGA  CCACTCTTAGCCAAACCACCAAACATCAATATTTTCCTTTTACCATAAAC  TGACAGTGTATGGCCCAACCGAGAAGGTGGGGTCCATGCTACAGGTATCT  CTCTCCAAACAGGTTTCTCCATGGAGAGATCAAGCAGGAAAGTATCACTT  AAAAGTACTCCAGAATCTGCACAACCACCAGAGACTATCAATTTAGTACC  ATCAAGAGTGCAGGAGCTGTGCCATGATCTTGGAAGTGGAGGAGCTAATC  CAGAGATTTCACGCCAAGTTGGAGGCTTGGCATCCAAATCTAAAACAAAA  ACATCATTGAGTAAGCCCTGCCTTCCACAACCACCAAATACCACCAAGTT  AGACCCATTTACACAAGAAAGTGTATGACCCCAACGACCAGGTGGAGGAG  AGCTGACTTGAACATGTTGCCACTCTGGGTTACTAGAATTGAGGTCCAAT  ACAAAGGTATCATTCATGGGTTGCATATTGACCCCTTCCCCACCAAAAAG  AACCACCCGATTTCCAACTGCACAAGCACTAAAGTTGCACCTTGAAGGTT  CAACAGCACCTCCAACAGTTAGCTTCCTCCATGCTGCAGCTTCAAGAGTA  GTCAATTCCCTTGCCAGTCCACCCCAGCCTAGTCTCTTTGCACCAGGCAC  AGTCTCTAAAACACGAGTTGTTTCACTACCCCAAGCATTCTGGCAGACCA  TTCTCCAGAGATCCTCATTTTTTGTCAGTTCATATAGTCGCAGACAGACA  GAACCAACTGATGCAATATCTCTAGGTGTCAACCGCGAAAGTATCTTAAG  AGACAGCACCTCATCACTCAATTGCAGTATCCCACAAACCCCACGACAAA  CATTTCGGTCTCCCACTGCAACTGGACAGTAAGTGGATAAACCAGAACAA  AATCGATCAGATGGTTTTGCAGATCCTTTTATTGAATATCCAGGCACTGG  ACCCAGATCGATATTTGTATCGGTGAAAAACTGTAT |
| CL1764.Contig1 | 3005 | 0 | 0% | AAAAAAAAAAAAAATGGGGAAGAAATTATAGTGGCTGAGATTGCTCCTGA  TTCCCAAACTGTGGTTTCTGGACTCTCTTAGGTCTCCACAACTAAATACC  CTGTCACTTCTCTGCTTCTTCATTTCTCTCTTCTCTCCATTTCTTACACT  GGATTTCCAGAAACCGTTTCCGATTCAGATCCCACCATTGCTGGTCACCA  TCAGTTCTCCGGCAACCTGCCGGTGAATTCTGTTCATGCATGATCAACAA  CCTAGCGCTAACCGTCTCGATGCTTCCAGACTTCTCCTCCACCATGGCTT  TCTCCAAAGGTTTTGGCGTCTACGGGTTCGTTTTGTAGTGTTGCCTTATC  AGTTTTTGTTGACTCCTACTACTACTACTACTGCTACAACGACGGCTTTA  TTGTTCGATATTAAGGGTAGAAATGAAGGCAGGTGTTTGTTTTATACGTT  GCTCGTTAGAGAGGATTTGAAGCAGCGTTAGCTGCCCAGGTCTGGTTATG  GACACCTACTCTTCTGGAGAAGAATTGATTATTAAGACAAGGAAACCCTA  TACAATAACTAAGCAGCGGGAGCGATGGACAGAGGATGAGCATAACAGGT  TTCTTGAAGCCTTGAAGCTCTATGGACGAGCTTGGCAACGCATTGAAGAA  CATATAGGGACAAAGACTGCTGTGCAGATCAGAAGTCATGCACAAAAGTT  CTTTTCAAAGCTGGAGAAGGAGGCTTCTTCTAAAGGGGTTCCGATTGGAC  AAGCTATTGACATAGAGATTCCACCACCACGCCCCAAAAGGAAACCACGC  AATCCTTATCCTAGAAAGACGTGCACAAGTGGTCCCACATTTCAGGTTGG  AGCAAAGGACGGAAAAATTGTAACATCAGTTTCTTCTTCGCATTGTCAAC  AAGTCCTGGACTTGGAGAAAGAACCTGTTCATGAGAGACCTAATGGAGAT  GAGAAGCCAACAAATACAAAAGAAAATCAGGATGACAATTGCTCGGAAGT  CTTGACCCTTGGCCATGAAGCTCACTGTTCCTCTGTTTCTTCTGTGAATA  AGTATTCTGTATCAACGCCAGTGGGGCACAGAAACTCAAGCACTTTTAGG  GAATTTGTCCCTTCACTGAAAGAGGATAATGGCACGAGTAAAGCTTTAAA  CTCAGAGACTAGCTCGCAACATAAGAAGTCAGTTCAAGGTGAGAAAATAG  ATGAATTTAATGGTGCATTGCCAGCAGATGAGATGCAAAGTACGCAGAAC  TACCCCAGGCATGTAGCTGTTCACATTCTAGGTGGGAGCAATGGCCCTTG  TATTCAAAGTCCTCAAAATATGCAATTTGAAGACTCCATATTTCAGCCCA  TGGTAGAAGTTCAAGGAAATCCTAATCTGTTCACGAATCCAGCTGCATCT  GCTGCTACTGAACATCAGAGTAATGTGCCCAGAACTACTCATTCATCATT  TCCAACTTTTCCTCCTCCCTTTACCCCAATTCGCCATAATCAAGATGACT  ATCAATCGTTTCTTCACATCTCCTCCACTTTTTCGAGTCTTATTGTGTCT  ACTTTGTTACAAAACCCTGCAGCCCATGCTGCAGCAAGCTTTGCAGCTAC  ATTTTGGCCCTATGCTAATGGGGAAACTTCTGCAGATTCTCCTGCATGTG  TGCAGGGAAGTTTTCCATCTAGACACGTGAACTCTGCTCCTAGTATGGCA  GCTATTGCTGCTGCAACTGTGGCAGCTGCCACTGCATGGTGGGCAGCTCA  TGGACTGCTTCCATTGTGTGCTCCTCTTCATGCTGCTTTTACCTGTCCTC  CTACCTCTACAACTGCAGTTTCATCAATGGATACTGGTGAAGTTCCAGCT  GCCAAGACAGAGAAGAAAGAGAATGCTCCTCCAAATCCTCCCTCAGAGGA  ACAACAGCCAGAGCCAGAACACTCTGAAGCTTTGCGAGCTCAACCTTTGG  CCTCTAAATCACCAACTCTGTCAGCATCAGATTCTGAGGAGAGTGCAAAT  GCAAAGCTTAATACAGAATTGAAAGCTGCTGATCGTGAGAAGGCTGCTGA  AGAAAATGAGCTTCATGATTCGAACAAGACAAAGACCAGAAAACAGGTGGA  CCGCTCCTCCTGTGGTTCCAACACACCTTCTAGCAGTGAAGTAGAGACAGAT  GCATTAGAGAAGCAGGATAAAGACAAAGAAGAGCCAAAAGAAGTTGATGCA  AATCATCCAGCCTCAGAGCCTAGTAATCGTCGAAGTAGAAGCAACACCAATAT  TAATGATTCTTGGAAGGAAGTTTCTGAAGAGGGGCGGCTGGCT  TTTCAAGCGCTTTTTTCCAGAGAGGTATTGCCGCAAAGCTTTTCTCCTCC  ACATGCTTTGGAGAACAACATGCTGCAGAAGGACAATACGGAAGAAGAGG  TGCAAAATGTCAATGGGCAAGATAGAAATGTATCACTTTTAGATCTCAAC  AGCAAGACATGTCCCGCCCATCCAGAGGAAGAGAAAAATGCACTGTGCAA  GGGTGAGAACAATGGGGAGGAGGGGCTGCTGACAATTGGGCTTGGATATG  GAAAGCTAAAGGCCCGTCGAACAGGTTTCAAGCCTTACAAAAGGTGTTCA  GTGGAAGCCAAGGAAAACAGGTTAGTTAACACCAGCATCCAAACGGAAGA  AAATTATCCCAAAAGGATACGCTTGGAAAAAGAGGCATGAACTTGAGATTG  TTGCATGCAATTAAGCAAGGGAAACCTTTGTTAGTTTGCATGTATTTCT  AATTTTCCCTTCTATCTGCTTTCATTTCATGTTTAATATCAAGTCCAGTT  CACGAGACTTTTTCGTGTAGGTGTGTGTACTATATGTCTAGATTGACTTA  TTTACTTTCCAGAACTTATGCAACTGTTCCTAAAATCTCATTTTATAGAG  AGACTTAGACAAGAACCATTAAGTATTGAATAGGCCCTGATTTGGCCTTT  TTACTCAAAAGGTATGTCGTCTACATTGTCTTTTATTTAGGTTTTTTTTG  CCCAA |
| CL1764.Contig2 | 2789 | 0 | 0% | AAAAAAAAAAAAAATGGGGAAGAAATTATAGTGGCTGAGATTGCTCCTGA  TTCCCAAACTGTGGTTTCTGGACTCTCTTAGGTCTCCACAACTAAATACC  CTGTCACTTCTCTGCTTCTTCATTTCTCTCTTCTCTCCATTTCTTACACT  GGATTTCCAGAAACCGTTTCCGATTCAGATCCCACCATTGCTGGTCACCA  TCAGTTCTCCGGCAACCTGCCGGTGAATTCTGTTCATGCATGATCAACAA  CCTAGCGCTAACCGTCTCGATGCTTCCAGACTTCTCCTCCACCATGGCTT  TCTCCAAAGGTTTTGGCGTCTACGGGTTCGTTTTGTAGTGTTGCCTTATC  AGTTTTTGTTGACTCCTACTACTACTACTACTGCTACAACGACGGCTTTA  TTGTTCGATATTAAGGGTAGAAATGAAGGCAGGTGTTTGTTTTATACGTT  GCTCGTTAGAGAGGATTTGAAGCAGCGTTAGCTGCCCAGGTCTGGTTATG  GACACCTACTCTTCTGGAGAAGAATTGATTATTAAGACAAGGAAACCCTA  TACAATAACTAAGCAGCGGGAGCGATGGACAGAGGATGAGCATAACAGGT  TTCTTGAAGCCTTGAAGCTCTATGGACGAGCTTGGCAACGCATTGAAGAA  CATATAGGGACAAAGACTGCTGTGCAGATCAGAAGTCATGCACAAAAGTT  CTTTTCAAAGCTGGAGAAGGAGGCTTCTTCTAAAGGGGTTCCGATTGGAC  AAGCTATTGACATAGAGATTCCACCACCACGCCCCAAAAGGAAACCACGC  AATCCTTATCCTAGAAAGACGTGCACAAGTGGTCCCACATTTCAGGTTGG  AGCAAAGGACGGAAAAATTGTAACATCAGTTTCTTCTTCGCATTGTCAAC  AAGTCCTGGACTTGGAGAAAGAACCTGTTCATGAGAGACCTAATGGAGAT  GAGAAGCCAACAAATACAAAAGAAAATCAGGATGACAATTGCTCGGAAGT  CTTGACCCTTGGCCATGAAGCTCACTGTTCCTCTGTTTCTTCTGTGAATA  AGTATTCTGTATCAACGCCAGTGGGGCACAGAAACTCAAGCACTTTTAGG  GAATTTGTCCCTTCACTGAAAGAGGATAATGGCACGAGTAAAGCTTTAAA  CTCAGAGACTAGCTCGCAACATAAGAAGTCAGTTCAAGGTGAGAAAATAG  ATGAATTTAATGGTGCATTGCCAGCAGATGAGATGCAAAGTACGCAGAAC  TACCCCAGGCATGTAGCTGTTCACATTCTAGGTGGGAGCAATGGCCCTTG  TATTCAAAGTCCTCAAAATATGCAATTTGAAGACTCCATATTTCAGCCCA  TGGTAGAAGTTCAAGGAAATCCTAATCTGTTCACGAATCCAGCTGCATCT  GCTGCTACTGAACATCAGAATTCTCCTGCATGTGTGCAGGGAAGTTTTCC  ATCTAGACACGTGAACTCTGCTCCTAGTATGGCAGCTATTGCTGCTGCAA  CTGTGGCAGCTGCCACTGCATGGTGGGCAGCTCATGGACTGCTTCCATTG  TGTGCTCCTCTTCATGCTGCTTTTACCTGTCCTCCTACCTCTACAACTGC  AGTTTCATCAATGGATACTGGTGAAGTTCCAGCTGCCAAGACAGAGAAGA  AAGAGAATGCTCCTCCAAATCCTCCCTCAGAGGAACAACAGCCAGAGCCA  GAACACTCTGAAGCTTTGCGAGCTCAACCTTTGGCCTCTAAATCACCAAC  TCTGTCAGCATCAGATTCTGAGGAGAGTGCAAATGCAAAGCTTAATACAG  AATTGAAAGCTGCTGATCGTGAGAAGGCTGCTGAAGAAAATGAGCTTCAT  GATTCGAACAAGACAAAGACCAGAAAACAGGTGGACCGCTCCTCCTGTGG  TTCCAACACACCTTCTAGCAGTGAAGTAGAGACAGATGCATTAGAGAAGCA  GGATAAAGACAAAGAAGAGCCAAAAGAAGTTGATGCAAATCATCCAGCCTC  AGAGCCTAGTAATCGTCGAAGTAGAAGCAACACCAATATTAATGATTC  TTGGAAGGAAGTTTCTGAAGAGGGGCGGCTGGCTTTTCAAGCGCTTTTTT  CCAGAGAGGTATTGCCGCAAAGCTTTTCTCCTCCACATGCTTTGGAGAAC  AACATGCTGCAGAAGGACAATACGGAAGAAGAGGTGCAAAATGTCAATGG  GCAAGATAGAAATGTATCACTTTTAGATCTCAACAGCAAGACATGTCCCG  CCCATCCAGAGGAAGAGAAAAATGCACTGTGCAAGGGTGAGAACAATGGG  GAGGAGGGGCTGCTGACAATTGGGCTTGGATATGGAAAGCTAAAGGCCCGT  CGAACAGGTTTCAAGCCTTACAAAAGGTGTTCAGTGGAAGCCAAGGAAAA  CAGGTTAGTTAACACCAGCATCCAAACGGAAGAAAATTATCCCAAAAGGAT  ACGCTTGGAAAAAGAGGCATGAACTTGAGATTGTTGCATGCAATTAAG  CAAGGGAAACCTTTGTTAGTTTGCATGTATTTCTAATTTTCCCTTCTATC  TGCTTTCATTTCATGTTTAATATCAAGTCCAGTTCACGAGACTTTTTCGT  GTAGGTGTGTGTACTATATGTCTAGATTGACTTATTTACTTTCCAGAACT  TATGCAACTGTTCCTAAAATCTCATTTTATAGAGAGACTTAGACAAGAAC  CATTAAGTATTGAATAGGCCCTGATTTGGCCTTTTTACTCAAAAGGTATG  TCGTCTACATTGTCTTTTATTTAGGTTTTTTTTGCCCAA |
| Unigene12806 | 1266 | 0 | 0% | GGCTTCAATGCTTTTCGTTTCATTGTAAAACTCCATACAATAACTTTACA  ATCTCGTTTGTTGGGAGCAAATAAATTCGCGCAGGCTTCCATTTCTTTGC  AAAATGGCACTGTTCCCATTTGAACATGGAGATAATACAAAGAGGCGAGT  GTGAGTACACCATCCCCTGCATGTTGCACAGGTGCAATACTTGCCTGATA  AGTAATTAAGTACCATTTCATCTTAATATGGCTTTTGAGCCACTGATAAC  CAGAACAAAGTGGAGCAAAAGTCATCCCAATTGAGGGAAGTGTCACGTTA  ACATCACACGAACCACTTTGATCCTGTTTAGAGAATTAGATACTTCACAC  ACACGGCCTGATCCTGATCAGCATCCTTCGATTGAACTGTTTGTGTTGTA  CTTCCACAACGGAGTGATGTAGAACCGTTTCTTGATCAGGTAAAAAGCTA  GCAAGGCAGGGAACTTGAGGAATGAAAGCTTCATAGATAACCAAATAGCT  TCAGAGTTGGGATTAATATCCTGAGGTATCATCTTCAGGAGAAGAGTCTC  TTAATGCCTGCTCATCCTCCTCATCTTCGTCTTCATCATAATCAGCAGAA  GCAGGTTGCCCATTAAGATCCACATTTACACCATTTACCTTCCTCACAAA  CTGCCCCCGCACACGGGGTCTTCTTTCAGCAAGCCTTTTGCGATTAACGT  ACCTGATCTTCTTGTCAAAACAACGCTCCTTCCTCTTCTGCCTAAACTTG  ATCAATGCAGCCTCTCTTCTGTCAACTTTATTCAACTTCACTTCAGATGA  AGGTGGACTCCCAAATGATGGCCATGAGTTAGGAGGCATCTGACCGTGTT  GTGGTAAGCACATACTAACCGGGTAATAAGGAAATGATGCCACCCCATTA  ACATGAGGAACACACTGAGGAAGGTGATTGTACTGAGATAGCATAGCTGA  AGGAGCATGATTCTGAAGGTCATGCAGGTTCTTTTGGTACAGTTGTGCAG  ATGATGGAACCATAACTTGATTCACAGCTCCTGACATATAATATGGGTAA  GTGGTTTGTGCGGGTAAACTTGATAGATCATGTTGAGTCTCATTTTGAGG  AGGTGCATGCTCCTGAGAGAACTTTTCATCCTTGAAATTCCTCTGTTGAG  GAAACTCCCGAGACACGGGAGTGGAGGACCTCTCCATGGAAAGAGAATCT  GGAACACTAGAACTACTTGGGAAGTCATCTCCTTGAGAATAGTTTTCCCA  TGCCTCTCCATTTTCA |
| Unigene7343 | 682 | 0 | 0% | CTCTCCATTTTCATGTATTTGGGTATCACTAACTACTTGCTGAATGCATG  GTTTACATTTCTCTTCGATCCTCACAATTTGGGCAGTCTTATAGTCAACA  TTAGCACTACCTTGGGAAATGTTTTTGGGCATGCTTGCTTTGACATAAGT  GAAGAAGGCGGACGACTCACCAATCTTTAATTCACTCTTCTTTGGACCAG  ATGAAAATTGACCTATTCGGCGGTCACTTATACCCGGCACATCAGGCCGA  CATTCAGAGAAATGAATTTGAGGAGGCTCTACAGCAGCAACAACCGCAGA  TTCATTCTCCTGATGAGATGCCATGACCAATTCTGGATTGGTACTCTTTC  GTGACTTGTCATCCGTGTCATCCGAGAACAGAGTCGTACTGGTTGTATTA  GCATCACTAGGGTCCGATGCCACCAGATCAAAGTCATAGTTCAAGATGTT  CTTTTCTGCCAGCCCGAGCATTCGCCGCCTTCTCCACATATGTGTCCACA  AGTTCAAAAGTTCATTAGTTCGTAGAGGCTTTACAAGATAGTCTGCCGCA  CCTAACCTCAAGCACTTGACAACAATGGAGACCTCATCCTGCGCCGACAT  CATGATAACAGGTATGCGACGGAGTTCTTTATCCCTTGTGATGTACTTTA  ACATCTTAAAGCCTTTAGTCATTGGAAGGTCA |
| Unigene836 | 829 | 0 | 0% | GAGCGTGAAATCTTCCCCACCAGGGCGCTGGGGCCATACTCTCTCCTGTC  TGAATGGTTCCTGGTTGGTGGTATTTGGAGGATGTGGAAGGCAAGGATTG  CTCAATGATGTGTTTGTTTTGGACTTGGATGCCAAACAGCCTACATGGAA  AGAAGTATCTGGTGGAACTCCTCCACTTCCTAGATCTTGGCATAGCTCTT  GCACAATAGAAGGTTCTAAATTGGTTGTCTCAGGTGGGTGCACAGATGCC  GGGGTACTTCTTAGTGACACATATTTGTTGGATCTCACTACAGACAAACC  AGTGTGGAGAGAAATCCCTACATCATGGACCCCTCCTTCTAGATTAGGGC  ATTCTCTCTCAGTTTATGGTCGGACAAAAGTTCTAATGTTTGGTGGACTC  GCCAAGAGTGGGCACTTGCGGCTGCGATCAGGCGAATCCTACACCATTGA  TTTGGGAGATGAAGAGCCGCAGTGGAAACAACTGGAGTGTAGTGGAGTCA  CAGGCATAGGCAGCCAGAGTGCTGTGGTTCCCCCACCTCGACTTGATCAT  GTTGCAGTCAGTATGCCTTGTGGGAGGATTATTATTTTCGGTGGCTCCAT  TGCGGGGTTGCACTCTCCGTCTCAGCTCTTCCTTTTAGACCCTTCTGAGG  AGAAACCATCATGGAGGATTCTGAACGTTCCTGGTCAACCCCCTAAGTTT  GCCTGGGGCCACAGCACCTGTGTAGTTGGAGGGACTCGAGTTCTGGTATT  GGGTGGACACACTGGGGAGGAGTGGTTACTTAATGAATTGCACGAGTTGT  GTTTAGCAAGCAGACAGGATTCAGACCCA |
| Unigene12571 | 1550 | 0 | 0% | TTGCACGCAAAAAATGCAAATGCATCCAGTTCCGAGGAAGCCTTATGCCA  GTTGCATAATCTTCAGCTGCCCGAAGAAGTTCCACAAGCTGTACAGCTGC  ATCAAGCGCATCTGGGGCCCATGATGGTGGCGCTTCTAGAAGTCCAAGAA  GAAGTCTTTGAGTGGCGCTTGGAGTTGCAATGGCATAATATCGATGAAAC  AAACGTGCATATGGCTCCAGGGCTGGAAGCCCAGCAACTAGATGCTCATC  CAAAGCCGTTGTTGCAGGAGGAAGTAGAAGTGCAGGGACAGCAGCAGCTG  TTAAAGTAGCAGTCTCATAGCGAGCAACTTCGTCATCACACACACTTAAT  ATAACACCAGCACCATTTGCAACTGCCCACCTTGGAGTTGATGGCATGAG  CTGAGGATGCTTTCCCGATCCGCGGGACGAGGCAGTTGGAGGTGGCTTCA  GCTCTCCAGCAGCATATTTACCCATGACACCACTGCACCAACGGAAGTAG  TCACTTCTGATAGCCAGAGGGGCAGCAAGCAATATATCAGTAATCCAGGG  AGACAAAGGCCTTAAAGGTTTTCTTTCCTGTTGCACCAATGGGACGTGGC  AGGTTTCACCACCTCCAGAGTTACTTGTCGTAGCATGGCTGCCACTACTA  CTTCTCTCTGTTTCACTATTATGTTGTTCCATCTTGTATATTGGGCGATT  GTAATGAGTTAAAATCCGCAAAATTTCTCCGCATGCCAGAGCCCACTGTT  CAGAGTATTCATTCTCACTACTAGGGCAGACTAAGGAAATGAAAGAAGCA  AATGGAGGGCTACTTTTATCATAAACCAGTGTTCCTTCAATGATAGAAGA  TATAATTGGAAGAACAACAGCATGCCCATGCTCCGGATGATGAAGGACAA  ACATTGCCAAAACATCATCAAAAAGGCGCATCTCCTTTGATGGATAACGG  TTGCGAATCAGCTCAGCAATATCCTCGGGGAATTGTTCAGATGTAAACTG  CCCAAAGTACTCAACATAGGCTGTGGTTTGAGCCTTTCGTTGTTGGGCAT  CTTGCGGTGGAGGCCAGAACAACGAGGAGAATTGAAGACCATCAATCCAC  CTCTCAGATGAACTGGCCATCTGATATAGGAAGACAAAGAGGAATATATC  TTCCAGACAGCTCCAATCTCAAATGATCAAAAAACCCTTCTTTTCGCTTT  CTAACCGCTACCAATCAAGAACCAAAATTTCGTAATTCAACATTCCAACA  TCTACCACCCCCAGAATTCACCAAATATGTATATATTATCAGTTCAAAAA  TAATTAATGAAGCTTTACAATTTTGAAGTCTGTTTCTGTTTCTGTTTCTG  CTTCTGCTACTGCTGCTGCAATTCTCCGAAAAACCAAAAAAAAAAAAAAA  TCAATTCACTACTCTCTCTATGCCTCTCAACAGCCTCAATCAAACTCCCA  AAATATCAACACTCTGATAGTTGTTGGGTGCAGAGAAGAAATCCAGGTCT  CCGCTTCTCTCGGTTTTTTTGGATATTTGCTTCTGTGAATACAATTGTCA |
| Unigene3692 | 2414 | 0 | 0% | CAGCTTACGGCTTGATTCCTTTAAGTTCTTCAGCAGTTGATCTTCCTGAG  ATAGTAGTTGCAACTCCTTTGCAACCTCCTATACTATCATGGAATCTCTA  CCTACCTCTCCTTAAGGTCCTGGAATATCTTCCTCGTGGAAGTCCATCAG  AAGCATGTCTTATGAAGATATTCGTTGCCACAGTGGAAGCAATTCTTCAG  AGAACGTTCCCAGCGGAGTCCTCCAGAGAACACACTAGAAGAACAAGATA  TTTTTCAAGCATAGGGTCTGCCTCCAAAAACCTTGCTGTGGCTGAGCTTC  GAACAATGGTTCATTCCCTCTTTCTAGAATCATGTGCCTCTGTAGAACTT  GCTTCGCGCCTACTATTTGTTGTCTTAACTGTGTGTGTTAGTCATGAAGC  TCAGTTCAAAGGAAGCAAGAGGCCGAGAGGTGAAGATGGTTATTTTCCTT  ATGAAAGTACTGAGGACTTACAAGTCACATATGAGAAACAAAGAGATGGA  AAGATGAGAAAGTTAAAAAAACAAGGGCCAGTAGCAGCATTTGACTCTTT  TGTTTTGGCAGCGGTTTGTGCTCTTGCCTGTGAGCTTCAGTTGTTTCCTT  TGGTTTCCAGCGGTGGTAATAATTCAAACTCAAAAGATGCACAAGCTATA  GCTAAGCCTGCCAAAATAAATGGATCTACCATTGAGTGCAAGAGTAGTAC  TGATTCTGCAGTGCATCATACTCACAGGATCTTAGCAATTTTAGAGGCAC  TTTTCTCCCTGAAGCCCTCCTCAATTGGTACCTCATGGGGTTACAGTTCA  AATGAGATAGTTGCTGCGGCAATGGTTGCTGCACATGTTTCTGAACTATT  TAGACGGTCAAAGGCTTGCATGCATGCTCTCTCTGTCTTGATGCGATGCA  AGTGGGACAATGAAATATATAGTAGGGCAACATCTCTATATAACCTCATT  GATATTCACAGCAAAGCGGTTGCATCCATTGTTAACAAAGCTGAGCCATT  AGAAGCACACTTAATGCATGCACCTATCTGGAAGGACACCGTTATGTGTT  TTGATCGCAGAAAGCAAAATAAACTTACAAACGGTGGCTGCTTTGATCCT  GGGCAACCATCTGCCTTGCAATGTGACAATTCAGCCCATTCAGAAATTCA  TCTCAAAAGTGAAGGAGCATCCCGTTTGGATGAAGGTTCAGGACATACCC  TGGGAAAAGGCCTTGCAAATTTTCTATCGGATGCCTCCGATTTGGCAAAT  TTCCTCACAATGGACAGGCATATAGGATTCAACTGTAGTGCACAGGTTCT  TTTAAGATCGGTGCTTGTAGAGAAGCAAGAATTATGTTTCTCTGTTGTTT  CACTTCTATGGAACAAATTGATTGCAGCCCCTGAAACCCAACCAAGTGCA  GAAAGCACTTCTGCCCAGCAAGGATGGAGACAGGTAGTTGATGCATTATG  CAATGTTGTGTCGGCATCACCAACAAAAGCAGCCACAGCCGTTGTTCTTC  AGGCGGAGAGGGAATTGCAGCCTTGGATTGCCAAAGATGATGATCAGGGT  CAGAAAATGTGGAGAGTCAACCAGCGGATTGTAAAACTGATCGTGGAGCT  AATGAGAAATCATGAAAGTCCAGAGTCACTATTAATTTTGGCGAGTGCAT  CAGATCTACTTTTACGTGCCACAGATGGAATGCTTGTCGACGGAGAAGCT  TGCACGCTGCCACAACTCGAGTTACTGGAAGCAACAGCTAGAGCAATACA  GCCTGTCCTGGAGTGGGGAGAATCTGGGTTAGCAGTTGCAGATGGTCTTT  CAAACCTATTGAAGTGTCGTCTACCGGCTACAATACGATGCCTCTCTCAT  CCAAGTGCACATGTCCGTGCTCTGAGCACTTCAGTCCTTCGCGATATACT  GCATACCACTTCAAGTAAATCTAATTCTAAACAAATCGAAATAAATGGCA  TCTGCAGTCCCCCCTATCAGTATTTTAACATAGATGCTATTGACTGGCAT  GCTGACACTGAAAAATGCTTGACTTGGGAAGCTCATAGCAGGCTTGCAAC  AGGAATGTCTATTCAATTTCTTGATACAGCTGCCAAGGAATTAGGTTGTA  CCATTTCCATATGAATTTAAATCAGGTTTGTCAGTATAATTTATTTTACC  AAATGAGACTTACAGATAGGAAATGGACCTTATCAAATTGGCTTCAAAGA  TATGGTATTGTCACAGAAACTATCTTAGTTCTTCCAAGTTGTACGTACAC  AGCATCTTAATATCCCCATGCTGTTCTTGATGATTGTGTATACGGTAGAC  AGGGCCTTGATAAGGTCTTATGTTGTGTGTGTAATTTTATTCTGCTCAGG  TTTGATATGTACCATAGACCGCGGACGAAGATGTAACTTGGATTTTATTT  CTAGCATAATCAGC |
| Unigene5963 | 295 | 0 | 0% | AGGAGGAGGAGGAGGAGGAGGAGTGTGGAGACGTGGAGGTTTGGGATT  CGCTGATCAGGAGCTTCAAGCAAGTGCAGACTGTACTGGACAGGAACCG  TGAGCTTATCCGTAGAGCCAACGACAATCACCAGTCGAAGATTGCAGACA  ACATCGCCGGCAACATCTCTCTCATCCGCCAGATCAACGGCAACATCTCCA  AGGTCGTCAAGATCTACTCCGATTTGTCCGTCAACTTCGCCGGCATGGTCCG  CCTCCGGATGACGACCACTCCTCCTCCGCCCGCCACTGACACCAG |
| Unigene4309 | 688 | 0 | 0% | ACCAACCCCATCAACAAAAAAAAAAAGAAAAAAAGACACATTAAACA  CTTGAATAATACTCAACTCAACCCCTTTTTTTATTTCATATAATAACAGCATC  AAACAATGATAATAACTTGTAAATAAAGCTCAAATTTTGAGGCCTCCCCA  GTGTCTCAATCGTCATCAGCGTGCTTGTTTTCTTGATGGAAAGCAGAGGA  GAAGTCAGAAGACATATCAGAGTAAAGAGAGACAACCTTGGAGATGTTTC  CATTGATTTCCTGGATCAAAGCAACGTTCTTGACCATGTTGTCAGGGATC  CTGGACTGGTGATTCTCATTGACTTGTTGAATCAACACCCTGTTCCTGTC  CAGAACAGACTGGACTTCCTTGAAGCCGCAGTTGAGTGACATCCACACTT  GAGGGTCCCCTCCAGCGTCTTGGTCCTCCAGAGTGTGGCTGCTCTTGCTG  CTCCTGCGGTGCTTGTTGTTGCGGTTGGTGGCAGCGGTGGTGGTGGTGTT  GGAGAGGTTGTCCATGGTGGTTATGGTGGTGGATTCGGCTTCGGAGGAGG  AGGAGGAATCGAGGGGTGCAGGTGTCATGTGACAAGTCAAGAGTCGGGCA  TTTTTTGTGGTGTTTTGGGTTCCACTCCCATTGTTGTTTTCAGACAACAA  TTGGTCGATATAGATTCTCTCTAGATGGGTTAGCGGTT |
| Unigene5237 | 2243 | 0 | 0% | GGGTTGTCTGGCTATGACTGTAACAACACAGTCTTAAACACGAATGTGAA  ATGGCAGTGCCACAACGTAATTATCACGTTTGCAAAGTCTAAAAACTACC  CTCCCCTACAGACATCGACCCCATTCCCTATATATTTGACACGATATTCA  TATTACAAGGACATCTTGTAACACTCTAGAGATATATCTACATGTGAATA  AACTATGAAGTCCATAATCACAATTTAGGAGGACAAAAACAAAATATTAT  GACTAAAAGGGGGAAATGACCAAGCATCCTCAAGCGGTACATAAAGTGTC  AGTGTATTTACCAGCCAAACAAGCTTAAATTGAGTCATACTGTTTCCTCT  CTTCTTGTATAGACTGGAAAATTCGAGCCACAGATTCAGTAGCTGATCTA  GGATTATGAGGCACAACTCTGATCACTCTTGTTGGCTGCTCTGTATCTTG  AGGCTGTGGGGCTCCCTCTGGAGGTACTGAAGCCATAGAGAAGTGTGGCT  GTGCTTCTTGTCCTTCAGCAGTGGCCCCTGTCTCATCGTGTTGCATTCTT  TCACCTGTGCTACTTGCTGTGCTTCCTTGAACATCCATGTCTTTAGATGC  TTGAAACTGCATTGCTTGTGGAATGGCTCCTTTCTTCTGAGTAGGAACAT  TACATGAGCTTTCGTGTTGCACATTAAAATTAGCTCCTCCACCAGATAAG  AGACCATTTTGAAGAAGGGAACCAGGTCCAGCAGACTGGTTCTTTTGTTC  CGCAGCTGAACTTGAGACAGATTGGTTCATAATTGGCATGCCATACGGAG  GGAAGTAACCATGGCCAACTGGATGAGCACCAGGCATAGCCCCAATTCCT  TGATGAGTATGAGGAGCTGGAACCCCATAGGCTGGATGCATAAAGTTACC  CGGTATTGGAGTTGGACCAAAAGGTCCACATCCTCCACCACAAACCGGTC  CCATGAATCCAGGACCAGGATATGGCTTGTATATGAGCCCTTCAGAAGGG  GACATGACAGGAATCAACCATTGCTGCCCAGGCGATTGATGGAAATACCA  AGGAGCCATCCTGGAATCAGAAGCCACAGAAGCTGCTGGTGGGTTTGCAA  GGTAGGACCCATAATTTGAAGGCTGACTACCATTTTTCACAGAAGACACA  GACATCTTCCCCACTGCATTTTCTGCTGAACATTCCATGTTATGTTTAGG  CTTCTCAAAATCATCTTTGCGCTTAACAATGTGTGGCAGTGGTTTTACAA  CATACTCTGACGGAAATTTCTTCGCAGGAGAAGAATTCAAAGAAGCTTTG  CCAAGGTAAGCACTGTCCTCAAGCAAAAGGTGCGGCGATCCGGCAATCAG  TTGTTGGACCTTTATCAGTCTATGCAACTCAAATACTTGAACTGCAAACA  CTCTTTGTTGATTGGCAATTGCTTTTCTCGCCTTCCAGAAATGTTTTTGA  CCTATTATTCCTACTACATCATCAGGAGAGATATCCAAGCCTGATATAGA  ATCCACCATGGAGGTTTCAGAGACAGCATCACTTTTGTCTCTATCTCCCA  ACTGTAGTGATCCACAAGTCTTTTCCTCACGATATTCACTATCAATATCA  GGCTCAATGGGACAGCTATTGTCCTCCCTTAAATGAGAGCCACTTCTTGA  CTGGTGAACAACTCCCTTATTTATATCTCTCATTGACTCAACACCATCAC  CACATCTGCGGTCAACTGGTTGTGATCTAGTTCTAGTTTCTGGCCGTAAA  GAAGTATCATTCTCATTTGGTCTGTTAAAATGAGATTCAGGGAGATCTCT  ACTCTCTTGAATTGGAGATGCACTGGCCTCCTTCACAAGCCCATCAATCT  TTTCTCTGCTTGATAAAGTTGCAGAAGGTTTTAATGAATGGTCTCTACTT  GAGAAGTTGGTATTTGGGTTCTCTTCACTTTGATGTCTTGCCTCTTGTCT  CAAATTGAGATCTGAAAGGCTGCTTCGTTTCGGATCGTTATCATAAACAT  TTTGAGACTTTAACGAATGACCAGGATAAGGCGGGCCATAGGGAGAAAGT  TTTCCTCCATCAACACCATTATGAGTTTTACTCGAATTTTGATTCTTTCC  TGAGTGTACATAAACAGGAACAGTAAAATCATCCTCATCACCAGCCTTCT  TTCTCTGCTCAAGCTGAGGCAAGGGAATGGTAAGAGTTGATCCGGCAGAC  TGACGAGCATGAAACTTCTCAGTCAAACGAGACGGTGTTTTAT |
| CL3477.Contig1 | 2611 | 0 | 0% | CGATCACAAAGTCAGCTGAGGCTGAGTCTCGTGTTTCATTTCCACCCTCA  GAAAACCATTCCGTGTGACCCACGGGCACCACACCAGTCTCTGGTCTCTC  ATCTCTGCACTTGAATGGGATGGTTTGAGCTCAAAACATGAAAAAAAATT  AAAAAAATCAAAAATTTAAAAAACTAAAAATTTAAATAAAAAAAAATCGA  GGGGGAGTGAGAATGGAGGAAGTCTCGACGGAGCCAGTTGTTCCGGCGGT  GAAGCAGGAGCTGAAGTGGTCAACCGCGCAGGTTCCGGCCGAATCTGACGG  CGGCGGCGGCGGCTGCACGGACACGGCGACGATGGAGGTGGCGGAGGTGG  AGCTGGACAAGGACATGCTTTGTCCGATTTGCATGCAGATCATAAAGGACGC  GTTCCTGACGGCGTGCGGGCACAGCTTCTGTTACATGTGCATCATCA  CTCACCTCCGCAACAAGAGCGATTGTCCGTGTTGCGGTCACTACCTCACC  AACAACCAACTCTACCCTAACTTCTTGCTCGACAAGCTATTGAAGAAGAG  TTCTGCTCGACAAATATCGAAGACTGCTTCGCCTTTGGAACATTTTCGTC  TGGCATTACAACAGACAGGGTTGCGAAGTGTCCATTAAGGAGCTTGACAA  CCTCATATCAATCCTCACGGAGAAGAAGAGAAAAATGGAACAAGAAGAAG  CTGAAAGAAATATGCAAATACTGCTGGACTTCTTGCATTGCCTACGGAAG  CAAAAAGTTGATGAACTGAATGAGGTGCAAACTGATCTCCATTATATTAA  GGAGGACATAAATGCTGTAGAGAGACATAGGATTGAGTTGTATCGTGCAA  GGGACAGGTACTCTGTAAAATTACGGATACTTGGAGATGATACTAGTGCA  AGAAAACCATGGCTTTCATCAATAGACAAGAAAAGCAGTGGTCTCATGTC  CAGCTCTCTAAATGTGAGAGGTGGGATTTCTGCAGGAAGTGTGCAACACA  AGAAAGTGGATGGAAAGGCTCTAGTAAGTTCCCATGGACTCCAGAGAAAG  GATTCTTTGACGGGGTCAGATTCACAGTGTTTTAATCAATCAGGTGTATC  TGTTGTAAGGAAAACGAGGGTCCACGCACAGTTCAATGACCTACAAGAGT  GTTACCTCCAAAAACGGCGCCAGTTGGCAAACCAACCACATATTAAGCAA  GAAAGTGATAAAAATGTCATACACAGAGAAGGTTACAATGCCGGTCTTGC  AGATTTTCAATCTGTACTAACCACTTTCACTCGCTACAGTCGACTGAGGG  TTATTGCTGAGCTTAGGCATGGGGATCTATTTCATTCAGCCAATATAGTA  TCAAGCATAGAATTTGACCGCGACGATAAGTTGTTTGCTACTGCTGGTGT  TTCCCGACGGATCAAGGTTTTTGATTTCTCTGCGGTTGTTAATGAACCCG  CAGATGTACATTGTCCTGTTGTGGAGATGTCAACACGTTCTAAACTCAGT  TGCTTGAGTTGGAATAAATTTTCTAAAAATCATATAGCTAGTAGCGATTA  TGAGGGAATAGTCACTGTTTGGGATGTAACAACTCAGCAGAGTGTGATGG  AGTATGAAGAGCATGAAAAACGTGCTTGGAGTGTTGATTTCTCGCGCACT  GAACCCTCAATGCTTGTATCTGGTAGTGATGATTGTAAGGTCAAAGTTTG  GTGCACGAATCAGGAAGCTAGCATTCTAAACATTGACATGAAAGCGAATA  TATGTTGTGTCAAGTACAATCCTGGATCTAGCAATTACATTGCGGTTGGT  TCAGCAGATCATCACATCCACTATTATGATTTAAGAAACATCAGCCAGCC  ACTTCATGTGTTCACTGGGCACAAGAAAGCTGTTTCATATGTGAAATTCC  TGTCCAATGATGAGCTTGCTTCTGCTTCTACAGACAGCACACTACGGTTG  TGGGATGTGAAGGAGAACGTGCCGGTTCGAACTTTTAGAGGCCACACGAA  TGAAAAGAACTTCGTCGGTCTCACAGTAAACAGTGAATACATTGCATGCG  GTAGTGAAACAAATGAAGTATACGTCTACCATAAGGAGATCTCGAAACCT  GTAACGTGGCATAGATTTGGTTCACCTGATATGGATGATGCCGATGAAGA  TGCAGGATCATACTTCATCAGCGCTGTATGCTGGAAAAGCGATAGTCCCA  CTATGCTAACTGCAAACAGTCAAGGGACAATTAAAGTTCTCGTACTTGCG  GCTTGAGTAAGTTGGCAAAGAAGACAAAATTTAACACCAAAAAGAGAGA  AGTAAAAAAGAAAAGGCATTGGAATATCCACTCATGCACTGTAATTCAATG  TGATGAGCTATTATTTGTATATGTCATTAAAATGTGAAAATAGGAAGATG  TTGTAGGCCACATATTTGTCGTGTAAATTCTGCTATTGTATGTATGTAAT  TAATAGTGGCATTTGGGGTGTTGTAGCATTAAACTGCTGATTGATGTTGT  ACCTAAATTTACTCTGCAATGCAGCCCAAAAAATTAATTCTGTCTGTAAC  ATTCTCCCCAACACTGCTACAAAGTTTATTAAAGTTTAGCATCTTTGTCG  TGGCCCACCAG |
| Unigene13951 | 1520 | 0 | 0% | TCTTCCTCAAGTCCTTACCATTTGAGTAAAAAATTTCCTCATTTCCTTCT  GTCTATTCTTAAATTCATGTACCATAATAATGTATATTAATGGAACATGT  ACCAATTTGGTGTCCTTCTCCGGAATCTTCGTTTCCATGCTACTAATAAC  CATGATAACACAATTCACCATCATTACCATTATCAAATGTGCACTTTCGT  CTGAGAATTTTCTTTTCTTCATACCAATTTTGAGTTCTCCGAAATCTCCA  TTTCAATAATAATGACGAGCATGATAACTTGACTGATTATGGTTATCTGA  GAATTTTCTTTTCCCCAGAAAAACCTGTAGAACCCCATCGGACCCACCAG  CCACAAAAGTGCACTGGTCGTCCTCAACTTGTCTCCAGCACACACTACTT  ACGAACCCACTTCCGGTCTGGACAGGAGGATCCAGCACAGCTGGCTCGAA  CCCATGCACCCAGATGGGTTCACCCCACCTTTTATCATACACAAAAACTT  GATTATTCTCCGAGCCGCAACCCAGCAAGCCGCCATGTCTCCACACCGAT  AACCCAACAAAGCTCCTGCAGTTGACGTGCCCTTTGTACGTGCGAATCGT  GCGAGAATCGCGTAAACTCCACATCTTCAAGCACCCGTCCGTACCAGAAG  AAACCAGCGTATTCGCATCTAGAAAACGAATATACGTGACCGTCTTTGTG  TGTCCGTCAAATACCAGGACCGGATCCGCCATCTTCCGGACATCGTATCC  GTACGCTTTTCTATCAGCGCATCCAACGGCCACCAACGCGCCACCGAACG  GATTGAACTCAACGCAGCAGACAGAGCTACGCGAGTTGCTGGGCTGCACT  TTGGCTAAGCAGCCGTTGAAGCGCGGGTCCCACATTTGCATGGTGCCGTC  GTCGGACCCAGAGGCGCCCACGAGCGGGTCCCAACTCGAGTAGTCAACGC  TCCAGACCCGTCTGCCGCCGTGCTCGTCACGTTCAAAGACCGGAAACTTT  CGCTCCAGATCATACTCCGTCACCACCCCGTCATAGTCGCCGGATCCGAG  AACCCGTACGCCCGACCCGGGCTTCCATTTGAGGCTACTCAGCTTGGCTG  GAGTGCATATACAGTACTCACACGCACTAGCATGGTCCAACAAAACCACC  CCACCATTACCACCCCCACCATCACCACCAAGAAGAGAATTGAGGGTATA  AACTCTGATCTTCCTGGCCATTCCACCTGTGGCGACGACGGAGTACTCAG  AAGGGTCGAACTCGATGACGCCGAGAGCGTCAGTTGCGGCAGCGGCGGC  GGCGTTGGAGGAGACAACGGTGGAGAGTTTGAAGTCCCATTCACATCTGG  GCGTGGTACCACTGGTCTCGTTTTGTTCTTCTTCTTGTTGTAGCTGTTGTC  TGTTTGAGGTTTGTTGTTGATTTGTTTGTGGGTTTTGGGTTGAAGAAGAG  GAGGAGATGTTCATTGCCCGTTCATGTCAAAGTTTGTTTTTGTTCAAAGT  TTATTTTGGATTTTCTCGGC |
| Unigene14978 | 522 | 0 | 0% | CCCAATTTCAGTTACGAAAAGGTTGATGTTGATACCATTATTGGGCAGCC  AGGAAATATGCATACTGGTTTAGAACATGATGCAAAACCAATTTCATGTC  GTGATCATGAGACTGGTGCAGTGCAGTGGTTTACCGATAGTTTTGAGGCT  GCAACTCAACTTCCTTCGACATTATTAAGAAACAATTTTGAGGAAAATCT  TCCTGACCAACATTTAGATACTGGTGGCCATGCAAATGATAGCCGTGATG  GTCATAAAGAACAGTCAGATCATCCCATTACAACTGAAGTAGTACCAGTA  TTTCCTAAAGTTAGTTTACATGAGTTAAACAGTCAGGAAAGAGATTCTGC  AATCTCGCGGTACAAGGAGAAAAAGAAGACAAGAAGGTATGATAAACATA  TCAGATATGAATCAAGGAAGGTTCGGGCAGAAAGCAGGACAAGAATTAAG  GGGCGTTTTGCAAAGATTGAACATTAAAGTACCCGTAAGCAGTCAGCAAC  TTATAATTTTGTATGACTGGGA |
| Unigene15508 | 1505 | 0 | 0% | AGTACCAAGTAACAAATGAATACAACTGTTCTAATCACAGCAAGCAGAAA  TGGAAGACAGTGAAAACTTAGAGCTGGAGTTAACTAGAAGCTACTCTCTC  TTAACAAGCATATACAATTTATCCTGTTGCTATACATGTCCATAAAAACA  AAAAGACAATATTGCATCTGCAAAGTTTCGTTTTTCTGCCTGACCTCACC  CATTGTTCTTTGGCATATATTAGATGGTGTGCTATACATGTCAACAGAAA  ATAAAAGCAAAAACTACAACTGGCAGACTTTTCCGGCTGAGACCCCTAGC  GATCATGAGCCTTGGCAACCCATGATGCCATGGCGACCCCCTTAAAGAAG  TGTGTCCAGAGTTATGGTGATGAAGCGGAGGAAAAGATTAATAGTTGACA  TAGTGATCAGGGATCATATATATGACTGAAACAGTACTTAGCATTCTCCT  TTTTCTTTAGAAAAGAATTAAGCAGGTGAAGTTGCGCGTTCAAATGGTAG  GAGATCATAACCTGTAGTTTGAACAACTCTGCTCAGAAGCTGTCAAGATC  CATCCTTTACGGTAATGGATAGTCAACCATCTGCTCCTGAGATTTATTGT  TGAGCATTTGAAACCAGTTTTGTGTTTTTTTTTTTCCTAACATCCACCCT  TTTCTCGCGTTGAGAACCTAGGCCCCTGAGATACAATCATACAACTTGTC  ATTTCACAGGAAAAAAATCCATCAATTTCATCCATTGTTTAGATAATAAT  AACCGACTCTTTCCAACTAAACATGGTTTGTTTCTTTCCTTTTAAAGCAT  GGTTAAAAAAACTGTACTCTCAGTAGCTTCTGGTTTGGCTCAGAGGGTCA  TAATCGTAAGCATCACCGGCTTTGACAAAGCGTCCCTTCACACGCCTTCT  GACATCAGCCCTTGCCTTACGAGAGGCATACCTCACTCTCTTGTCAAACT  TGCGGGTCTTTTTCTTTTCCTTGTAACGCATGACAGCTTTACTGCGGCTT  GCTGATGTAAAGGAAGCCTCAGGACATGGAGGGCACCATGGTGGCTCCCC  CATGAGAAGCATTGAAGAAGCATCACAATCTTGGTAATCTCCACCATTAC  TCTCACCAGTTAGGCCAGAAAATGAGAGGCCGGAGTGTCCTTGCTTTGTG  TTGAAACATAGGATTGGTTCAGTCTTAGTACTCATCATGGAATCTGCAGA  TGCTGCATTGCTGCATGCTGGCTGCATTGCATTGACCAGTCCAACTGACG  AACCCTCAGCAGCAACTGCACCCTGACAGTTGGAATCAGCAGCAGACATC  TCCTTTGTCCCAAATAAGCTGTCAATTCCACCATTTTCAAAAAGCTCTTC  TGAATGATTGAGAGTCACACCAAAGAGTTCTTCATAATTCTCAATATTCA  AATCCACTTCATCCATATTGAAGTCCTCATAGAAGTCATCATCTTCACAG  AAAGCAGGACCTTTCGTTGCAGGACAGTATAACTTGGGCAAAGCCGCATT  ATTAG |
| Unigene15973 | 993 | 0 | 0% | CCCCCTCCCTCTCCTTCTCATTCACCATCAACACAAAACAAAACGAAAAA  TTGAAAAAAATAAAAATAAAAACAACACGTAATAACTAACCCCTCCAATC  CATCATTTCTCATGTGCCTCACTTCCACCATAATAACCACCAAAAAAACC  TCAAAAATGTTATTTCTTGCTTATTCAATCATCCTCAGCTAGGGTTTTGA  AAAAAGGAAAAAAAAAAACCCAAAAATAAAAAATAAAAAAATCCATACTT  TGAAGGAAAAATAGAAACTTTTTTTAAATCTTGAAGCGAGAATATGGTGA  ATCCGAAATCTGGGGGTGCGAAAGAGAGTGTGCCGTGTGATTTCTGCAGC  GAGCAGGTGGCGGTTCTTTACTGCAGAGCTGATTTGGCGAAGCTGTGTTT  GTTCTGTGATCAGCACGTGCACTCGGCGAACCTGCTGTCAAGGAAGCACG  TGCGCTCGCAGATCTGTGATAATTGCAGCGGCGAGCCGGTGTCGGTGCGG  TGCGCGACGGACAACCTGGTGCTGTGTCAGGAGTGCGACTGGGATGCCCA  CGGTAGCTGCTCTGTGTCCGCCTCACACGATCGTGTCCAAATCGAGGGTT  TCTCTGGATGCCCGTCTGCTCTCGAGTTGTCCTCCATCTGGGGTTTCGAT  CTTGAGGACAAGAAGCCACGTTTTGGTGGTGGGTATTGGAACAACGAAGG  AGGAGGTCAGGATGTGGTGGTGGGTCCAATGGAGCTGTCTTCGTGGATGT  ACAAGCCACCGATGGGTGCGGGTGGTGGTGGTGGTGGAGGAGTAGTGAGT  GTTCAAGATTTGATGGTGCCAAATGATCATTTGATGTTTTCGAGGCAGAG  TCCGAGTTGCGGGAAGCTTAAACAGGTGATATATAAACAGTTGGTTGAGT  TGTTGAAGAGGGACTTGATGGGAAATGAAAGTGGTGGTAATGTTGATAAC  AATAATGATAATGATAATAACAACACTAGTGTCAATAATAATA |
| Unigene16245 | 557 | 0 | 0% | CCACACTACATGATTCATTCATCTGTTATATTACAACATCAAAAGTTTCA  CTTGCCTTCACAAACCGACCTTTCACCCGCTTTCTACTATCAGCCCTGGC  CTTCCTGGACGCATAACGTATATGCTTATCATATCTTCGATATTTCTTCT  TCTCCTTGTAACGTAACATCGCCTTGCCTCTATTTTGCGCTAGCAATTTC  ATATCAGTCTCAGGTTTTGTCCCGCTTGTAGTTTGATTCCTAGCTGGAAC  AGATTGAGGAGTGACTTGATCAGGTCTGGTGTGGTCCCAGGTTTCGGATT  CAACCTGTCTGCTTGTTGATGGTGCTCCTGTAACTGGTGTGTTATTGCTT  TCTTCTGTTGTTGGTGCATAACTTGATAATAGTTGATGACACTGATTCTG  AGCAGCCTGATGTGCAGGATTAGAATCCCAGATAAGACGCCCTTCATCAA  CTGAACAATTAAAGTCTCTCAAATCCACAAACGGAGGCGACATTACCAAA  GACACACATGGCGTTTGCTGTTCCAGTAACTCATCATTACCTGCATTTTC  CCCTCCA |
| Unigene3300 | 1546 | 0 | 0% | CGACCCCAAATTTGTGATTTTATATAGGAAGTACACAATCCACTTAAGTT  GCAACTCTTCAGGTAAATGCTTAAATTCCGCTCATTTCAAACCTAAAGGA  TGAATTCTCCACCTTTGCATCAATAAGATTACCGATTTAAATCGGAATCA  TCCAAACACGTGATGATTAACTAAAACAAAGATTCCCAAAAGAGCTGTAC  AGCCCAAAAACATCATGGATTTTTTTTGTTTTTTTGGAACTGAAAAACAT  CATGGATTATCACAAATTCATTACATAAAAACTCCTAAAAAGATCTCAAC  CTCACCCTAACCGTACTTTATATACAAAAAACCCCCAAAAAAAAACAAAA  CAACAATTTTATTCCATCAAAATAAAAACTGAAATATTAAAAGGAGGGAA  CGACGCCGAATCCATAAAGACTACTACTACTGCTCCGGTCCGCTTCGATT  TCCGAGTCCGTACGCTTGGCGAACCTTCCTTTTATCCTTGGCCTCATCTC  CGCATACGCTTTTCTCGATGCGTACCGTATCGTCTTTTCAAACTTCCGGT  TCTTCCGCTTCTCTCTGTACCTCATTACACGCGCCTCCCTGTCCGCTGAC  GACAGCTGCTGCACCGTCTGATTAATCGACTCCCCTCCTGACCTCCCGTA  CGGATTCGATATGTCCGTCATCGTGCTGCCATCTGGCACCACTCCTACAT  CCAACGACGATGACGATACACTCTGGCTGAGGCACTGGGCGGTGTTGTAC  CCGAACCCGAAAGGCTTCGACCCGGGGAAATCTAAGTCAAAACAGGAGTC  GTTAACTAACGGCGCGTTAACGTTTTTGCTCTGAACCGGGACGACTCCGT  CCGTGCCTGAACTGTTCTGTTCCCGGGCTTCAAGTTTCGGATCCGGATGC  CCGTAATCCAGATCCAAATACGGATCCATCTCAGAAAACACGAACTGACC  CGTATTCAAGTCCGGATTCTCCTCCACTGCTTTCGGGTTCGGTAACAGCC  AGGAAGCCGCTTCGGCTTCCTCTCTGCTGACGTCTCCAGTGGCTCCGCCT  CCACCTGCGGCGTCGGCGTCGTCGAAGTAGCTATGGTCATCGAGGAAGCT  GGCGACGCCGCCGTTGTGCTTGGCGGAGGAGCTAACGGAGTCGTAGAAGG  GGGTGACAGGAACGCGCTCGTGTCGGCGAGCGAGCGGGTTGGCGGAGT  GGATGTCGTGGTCGCAGGTGACGCAGAGGGCGGCGGCGTCGGCCTTGCA  GGTGACGTGAGCCGGCGCCTGCTCGCAGACCTCGCAGACCCAGACGCGCG  CATGGCGCGAGGCCAGCTTGTTGGCTGCGTGGATCTTGGTGTCGCAGTTGA  CGCAGAGGAAGGCCGAGTCGGCTCGGCAGAACAGAGTCGCCGTCGCCG  ATTTGCACGAGTCACAGAGCTTGGAAGCCATTTTCGAATTCACTGAGTCA  AGCTTTTTTTTCCTCAAATCTCACTCTCTCTCTGCGTCTCTGCGTCTGTATTAT  TACAAGACTTTGAAATCACAGGACACAAAAACAAAACACAAACAAC |
| Unigene5163 | 433 | 0 | 0% | GCAGAAGATGGAAGCAGAGGAGGCACCACAGCTGTCACAGAGCTGGGAGC  GAGAGTGCTTCGAGAAGAGCTGGTTCGTGGAGTGTACTTCTCTATCACAC  AAGAAACAAAGCTTGGCTGAGTCAGCTCTGCAGTACAAAACAGCATTTCT  ATCATTACAGTAGTCGCAGAGTCGCTTTTTTTGCTGTCCTGCTGGCGGCG  TCAACGTTTCCTCGGTCATTTCTTGCTCTGTTTTCTTGATGGCGTTTGGA  TTTTTGTTGCTTTGAAGGAGAGCCAAAAGCTGAATAAATAAAGCTTTGAA  ATGGCGCTAAGTTGCTTATCGTTTCTTGGTGAGCAAGAAGTATCTTGAAT  ACAAAGTTTGATTTTTTCTCGTGCTCTTCAAATGAAGAAAGTGATATTTT  CACCTTTTGTAATCTGCAATGAGGAAAAGTTGG |
| Unigene7324 | 1122 | 0 | 0% | CAGGGAACAAGTGGATAACAAAAAAGAAATAAAACTGCAAAACATACATG  TCATTAGATTAAGAAGGCTACAATAAGTTTAGTAGAAGTAGAGTAGTAAC  AGGAGTGCTTGTTGCTTTAGAACGAAGGAACAATGCCATATCCGGCGTCT  GTGATCAACGTTGTGGAGAACATCTGGTCGACTTCAACTTCAACGTCTGT  GCGCTTGGCAAACCTACCCTTGATTCGTGGTCTGGTCTCTGCATAGGCCT  TCCTGGAGGCATACCTGATTGTCTTCTGAAACTTCCTAGTCTTCTTTTTC  TCTCTGTATCTCAGGACCCTGGCCTCCCTGTCCATTGGATTAAGCTGGGG  AGGCATTTGAATTGGAGGTCCTGAAAAGAGGTCGATTGTACCTTTTGGAG  GTCTCGAGTGGGAGATTGATATGTCACTTGTTGACTCTGGTACTAAACCC  ACATCGGTTGATGAAACTGACACACTGTGACTAATAGAACCGTTGTAAGC  AGCTTTTGAGGATTCATAGTCCAATCCCAGTTGAAAATTCTGCTGATTCT  GTTGATCTTTAACTTGGTACTGAACAGGAACAATGCTGTCACTGATATAA  TTCTTCCCAAAGTGTTGTTGATGCTGCTGATGGTGATTGTAGTGATCAGC  ATACTGATTCTCACCATTGTAGTCTACAAGGTCCAAATATTCATTAACCT  CTTCAGGAAACAAGAAGCTATCATTTGTGTTGTTGTTGTTCTTTGTTGGA  TTCATCAACAACCAAGAAGCAGCCTCCTCATCATCATCTTCATCTTTAGC  ACCTATGAACCCATCCTCAGTCTGACCATAGATGGACCCTGACATGGGAA  GTACGGGAACTCGGTGGTGACGGCGGGCGAGGGGGTTGGCGGAGTGGAT  ATCAGCATCACAGGCAGCGCAGAGGGAGGCAGCGTCAGCCTTGCAAAGG  AAAGCAGCAGGGGCCTGCTCACAAGCCTCACAAACGAAGACACGCTCGTG  CCTGGAAGCAACCCGGTTGGCAGCATGAACCCTGGCGTCACAAGCAGCAC  ATAGATAGGCCAAGTCAGCCTTGCAGTAGACAGTGCAAGCAGCAGACCTA  CAGGTGTCGCAGACACGTGCCCTG |
| Unigene7468 | 1044 | 0 | 0% | GGTAATGTTGATAACAATAATGATAATGATAATAACAACACTAGTGTCAA  TAATAATAATAACAATAATGTTGGTGGTGGTGGAGGAGAGAATTTGGTGC  CAACTACGCCGAATATTAAGAGTGGATGGCTTGAGAATGATATGGAGGCG  ATTAATGGGTTGGGAAATGGAGTAATGGAGGTGAATGGTTGTGTCCAGGA  GCTCTTGCAGCAGCAGACGCCCTTCACTTCTCTGCTTATGATGCAGCAGG  GACATGTGGATGCCAAGGAGACTGATGAAAACATGTTGTGGAATGCTAAC  TCTGGTGGTGGTCAGGGTGCTCAGATATGGGATTTTCATTTGGGACGATT  GAGGGCCAATGATGAATCTAGCCATTTAGAAGCTGGATATGGTGCTAATG  ATGCAGTATTCATGATCAAGAACTTTGGTGAACTTATGAAAGATGCGTCT  ATGACCAATACAGAAATGTTCGGAGATATTTACCAGATGAATTGTCCCAT  CACACATGATGATATGGCATCCTTTAATAATAATAACTCAAATAACCCGG  CGGCAAGTCAGGGCCCTGCAACATCTGAGAGCAACAACCTGCCAATGGCA  AGACCGTCATCAGGATCAGCTTTTGGTAAGCCCAAAGGCTCTACTAGCTC  AAAGGATACCCAGCTCATGGAACAGTCTTTTCTTGTTGGACATGATGGTA  TGGGCATAGCTTCTGCAACCAAGGCTGATCTGGAATTGCTGGCACAGAAC  AGAGGCAATGCCATGCAACGTTACAAGGAGAAGAAGAAAACACGGAGGT  ATGATAAGCACATCCGGTACGAGTCTAGGAAGGCAAGAGCCGATACTAGAA  AGCGTGTGAAAGGTCGATTTGTGAAAGCTAGTGAAGCTCCTGATGGTTGAA  CGAGATGCTGAGATACAGCTCCTTCCTTTCTCTATTGTACATATTTTTG  TTCACATTCCTTCCACAGTAAATGCCAAATTCAGCAGTCTTTATTTCTTT  GCATCTCATCTCTCTCTGTGTCTCCCCCTCTCCCTGTCCACCAA |
| Unigene3148 | 758 | 0 | 0% | AAATCGGATTACCAAACTATAAAACAAAAATTGAAAATCTTGAATAGAGT  GAAAATGGCAGCAAAGGCGGCGATCGGTTGCGAGGCGCTGGTTGGATCGC  TGACACCGTCAAAGAAGCGAGAGTACAGAGTCACCAACAAGCTCCAGGA  AGGCAAGCGCCCTCTCTACGCCGTCCTTTTCAACTTCATCGACTCTCGCTA  CTTCAACGTCTTCGCAACCGTCGGTGGCAATCGCGTGACTGTGTACCAAT  GTCTTGAAGGGGGAGTTATAGCGGCATTGCAATCTTATGTTGATGAGGAT  AAGGATGAGTCGTTTTACACTGTTAGCTGGGCTTGCAACGTTGATGGGAG  CCCATTCTTGGTGGCAGGAGGTATCAATGGCATAATTCGCATCATTGATA  CTGGCAAAGAGAAGCTACACAAGAGTTTTGTTGGCCATGGCGACTCAATA  AATGAAATCAGGACTCAGCCACTGAAACCGTCGCTTATAGTGTCTGCAAG  CAAAGATGAATCCGTTCGTCTCTGGAATGTGCAAACGGGAATATGCATTT  TGATATTTGCTGGAGCTGGAGGTCACCGTAATGAAGTTCTGAGTGTGGAC  TTCCACCCTTCAGACATATATCGCATCGCAAGTTGTGGCATGGACAACAC  TGTTAAGATTTGGTCAATGAAAGAGTTCTGGCCATATGTAGAAAAGTCAT  TTACATGGACTGATCTTCCTTCGAAGTTCCCCACAAAATATGTACAGTTT  CCTGTCTT |
| Unigene8962 | 744 | 0 | 0% | GCTCTCAAATAATAATGTGATAAAGTAAATAAAACAACTTTGTCACATTC  ATTAATGCTTTCCCACACTTTATTTAATCCATCTTAACCCATTTTTTAGC  AGTAATTTAGGAAGCTTCTAATACTAAATAGCTACACTTCATGCAACCTT  TAGATATATGCAAATATGATGGCTGGGAATCTTTAGCAACCTGTAAGGCC  ATTTGAACAAGATCAGCCATGTCAGCCTCAACTTGATTAAGATGTGATGA  TTTGACTGAAAATTCATGTAACTGATCAACTCTAGATTCATGTCTCCTGC  ATAATTTAACTTATAATTTATTCACTCTCTCTCTCTACTTAAAAGAGCTA  AAACTGCCTTTAGATAAAGATCTTCCAGGAGACCCACTTGGATAGATTCA  AGAAGTGGGTACAGCATCCCAACGCCATATCGCTCCATCCTCGCAACAGC  TAAGAATAGTGCTTCCATCAAAGGACACTGCAGTCTGTCTGATTGGAGAC  TTCGACTGAGTATGAGACAACCTTGCGATGAGAACAGGGGGACTACTTTG  CAGCTCCCATACATAGATCTTCCCTTCCCGATTCCCTATCACAGCAGTAT  TGTAATGGAAGTCGCAGGAAAACTTGATGAACCAGATATCACATTCAGGA  ACAGGGTATCTTTGAAGGATGTCAGCAGTGCCCTCCCCAGGAGACTGTTC  CTTCATTTTTGGTTCCCACAACACAATTTCATTGTCTACACTCT |
| Unigene11356 | 450 | 0 | 0% | GCCACTTTGAGTTTTGGTTGAAGAAAAGGCAAAAGATTTACCGGGATTAT  TTCTCTTGGAATTCTGGGAAACTAACTTTAGAAGGTGCTCTATCATCCGA  AATTTAGTATAACCACTGTACTTTCTCTGCTTGCCCATCTCTGCACAGAT  GATTTCAAGAAGCTCCCTGCGGCTAAATGAGCTAAGGATCTCTGGGGCAT  TTTTGGACCATTGGGCAATTTCATGAACCAGTTCTCTCTTCTCTATCAAA  CTAAGTTGGCTACATTTTGCAGGGTCAAGTACAAATCCTGAGAACATTGA  GTCCATTCCTGGAGTCCCCTCCTCTGCTTCGCTCATGACACTCCAAGAAC  GCAGCAGAGACAACGAATTCAACTTGTTTTAGAGAGCACAATGTGATGAG  TTCATGAAGCAGAAGAAATGAGAGAGAGAGGAAAAAAATCACACACAATG |
| Unigene13871 | 1792 | 0 | 0% | TCTGTAAAACGTATCAATTTGATTAGGATGTATAGCTTCAAAAACAGACA  TGGAAAATGTTAATAATTATGGTGTAAAAAGCACCCAATATGTGGTGCAT  TGTTTCTTCCATGTCATTGCAAGTACAAGAGCTCCTTCATGCCAATGCCG  TCCAATTCTCAAAATAATAGTCACTGCTCCTGTGTGATTCTGATAACTTA  GGAAATGAAAATTGTCTTCTAGACATTGAACTTACCGAAGGGGTCCACCT  TCGAGAAAAATTAGCAATTGTCTCCGTAATCAGCAGCTCAAATCATTTAG  CTGGACATACTAGTCCACAACACAATCATCACTTCTGAAAGTGATTATGA  AAAAATGCCTGTACAAAGAACAGTGAAACAATCAGAGGTAATCTATTTCT  TTGCAAGGAAAGAATAATCATGAAGCAAATAGCAATCCTGTTTATTAGAA  TGCCCCGCCCTAATCTAATGCCACAGCTTACTACAGAATCCGTTCCGTGG  CCTCTTGCTGGATATGATATCTGAAAAGGAGTCAACCAACTGTCCTGCTA  AGCTACTTGGATCATCAATCAAAGTTTGAATAAAAGTATTGACCACCCTA  CGTTCCTGCTCAGTCGATCTCAAGCTAAACCATGTTAACAATTTCAACCT  AAAATCCTGGTTAATGTGACCCTCACATTCCAGCCATCGAATTATTTTCA  CACAGTACTCAAAATTCTCATCTAAGCAACCTGAATTACTGGAAATGCGG  AATGGTGACCCATTTATCAAAGTGCTGTCGCAGTCATGTGCCTCTTCATT  TGAATGTGCTGTCCTTTTCCTGCACAACTCCGCCCGGGAATCAACAGCAG  GCACTTCTCCAGCTGGCCCATTTGCCCAGGTTCGGGAATCACCACTGCCA  TGGGATCTTGCTAAACGATTCTTCTCTACATCATCAGAAGCAGCATCATC  GTCTGCCTCAACAGCCTGCTCAAAAGTGCATCCATTATCTTCATCTCTTG  AGGACTCAAATGGAGGGGTTAATTCTTCATTCAAATCAGGCACTTCAACA  TTTAAGTCAAGCCCACGTGAAACAGATGGCAACTGATCTTCTTCTGGATT  TTCAGGTTTGACCATTTTGTTTGCTCCACAACACTTCTCTATGTCAGCAC  TGCAAAACCCTTCAAGGCAGCCTTTTTCCTGAGCCCAGGCCAAGCGCAAA  ATCTTTCCAAGGTCCCGAACCTTGAATCCAGAAGAATTAACTCCCGTTGT  AATTTTGGATTCCCTCTCAACACTAGAAGAACTTCCTTCAATGGGAGAGT  TTACTTTCTTACAATTCACGTCAGCTGTTGAATTGAGATTCTTATGAACT  ATCTCCACACTCTTGGTGAAACACTTGGCCTCAGAGTGACCCAAGTCACC  GTTCTCTGTGGAGGAAACAATCCGAAAACTGTACTCTGTACAGGGCTGCA  AATTGGATATCAAGATCCTTCTCTGAGACCTTGGAAAGACAGAAACAGGT  TGTATTGTATATGGTTCTTCTCGACTCTTGCAATACCAGAGCTTGTAGCC  TGTAATATTATTAGATGAAGCAGGTGATAGCTCCATCAAAATAATTACAA  CAGAGGAAGATGTCACTTCTTCAAATAGGAACCTGCAAGCAGCAGGAAGT  GAATCCTCTCTATGATTGGGATTCACATTAGAAATGGTAGCCAGCCATTC  ATCTGCCTTCTCTATAGCAAGAGAGCAAAGTTTCTGCACATCAGCAGCGA  CAGCAAGCCTGCTGACAATGCCACGTGCCATCTTTGCTGATA |
| Unigene15146 | 957 | 0 | 0% | ACGGTCAATCCATAGAGATCTCAAAGTGTGCAGGTCAACTCGTCATCTAA  AACACTTCAAGAAAAACAAAATTTGAGAGCTTCACGTTTCACTTCCCATA  AAGAACTAACCAACTATGATTCCGTGTTGCAGAAACTCCTGTCTCGACTA  ATTCATAGGGTTTCCACCTGAAAGAAAATTTGCTTATACTGCCTTTAATG  CCACCACCTGGTGCACAACCCATTTCCAAGAGCTGGTTTTTGCTCACAGC  AGATCTCGTCCATGAACGTGTGGATAAGCTGCCCCGCTAAGCTTGGCGGG  TCGTCAATGAAAGCATCAACGAAAACGCTGACCACCCTTCTTTCATGCAT  TGTTGCCTTCAGACTGAACCAAGTCAGAAACTTCACCCTGAAATATTCTT  CTATGTGCCCTTCGCGCTCTAACCATTTAACTACTCTGACAGAATACTCG  TAATTACTCTCTTCCCGTCTCTTTTCACAAACCAATCCTGGTATATCGCG  CATTCCATCAGATTTACCAGGTGTGGGAGGAGAAATTGATTTTGGACTTA  ATTCTTTGTCTTTGTTCTTGTTGATGTCTGCCAGGGATAGTGGAAGCTTT  GGAGGGTGCCCACCGAAGATTGATTTCATATTGCTAGAGTTCATTGATTC  CACCTTAGAGTGAATCTGAGCTGCTAAAGTGTTTTCTTTCTTTCTGTCTA  GATTGAACGCTGCAACAAAGCTTCCACTTGATGCAGCTGTGATCCATTTA  GCTTCCCAAACACCTACGACTTCTGTGCTTTTGAACAAAGAAACCTTGCA  GATGTACTCTGTTGAAGGATCAAGGTCAGTTATCAAGAATCTCTTCTGCG  GCCTTAATACAATATACGTAGGTTTGTCTGGATAATCCGTTACAGCAGAC  TTACGATGCCACAGCCTGCAGCCTGATATGTCTTTTAGTAGATTATCATC  GTATTCT |
| Unigene262 | 630 | 0 | 0% | CGAAAAAGTTTTTATTTTCTCTCTCTTTTTGGATTCGTGACTTCTCTAAG  CGAAATTCTCTGGGTTTTGTTCCATTAAAACCCTAGATTCCTCCCATTAA  TAACAGGGAGGCGTGGTAACACATTTTATTGAAGTTATAAACAATGGATT  TAGAAGATAAATTCCTCGCTAAAGTTTCTGGTGTTCAAAGCCTTTCTTCT  AGTGTGCAAAGCACCCCAGAGAAAATTGGGCATTCAGATGATGCCTCCAG  AAGCCCAGAACTCCTCCAAGAGTTTCTGAAATCTGGCCCAAAGAAGGAAC  TTCTTCGTAGTTGCTTTGATAAGGACAAGAGAAACTCAGCTTCATCGAAA  AGCAAAATGACAGAAATTCCTAAGGCTAATAACAAGACAATTAAGAAGCA  GGAAAAAAAGGTTTCTTCAAGTCCCAGTAATCAGCTTTCCAGAAAGCAAC  AGAGGAAGGGGGAAAACCCCATGCGGATTCCACCTGCTTCTGAGCAGTCT  CCAGATTTTGGATGTTCGAACTCATGGATCTGTAAGAATTCTGCTTGTAG  AGCTATTCTGTCCATCGATGACACATTTTGCAGGAGGTGCTCTTGCTGTA  TCTGTCACTTATTCGATGACAATAAAGACC |
| Unigene26983 | 293 | 0 | 0% | TGGATGAAGCTGTTAAAAAGCTTGTGTTGTAGATTTTGACAAGATGGTGC  TGGAAATCATTTTTTCCAATGACTCCAGAGCACAAGCACAAAGTCTTTGA  ACTTCAGGTCCTGAAGAAAGCCTGTTGACAATACCCCTACCCATCTTTAC  AGGTAAACCAGTTAACGGACCTACTTCATCCTCAAGCTTTTTAACAGCTT  CATCCACAATCTTAGAGATGCTTTGGTACTTTTCAGTCCCATGGAGAAGC  TTCTGACCTAACGAGACACGATAACACAGTATATCCACCCGTC |
| Unigene5939 | 286 | 0 | 0% | CTGAACAATACAAAGAAGTACATAAAAAGGTGGAGACTGCCTTATATATA  TTAACGAATGAAGTAGGGCCTCCGAATCTTTTATGCACAAAGATGGCACG  AGGTATTGTCAACAGACTCTCTTGTGGTGCTGAGGTCCAGAAGTTGTGTG  CTTCTGCAGTGGAAGCATTTGATTCCATGCTTCTTGTCGATCATGTGGAA  AAGAAAGAGCCAGCATCTTGCCAGATCCAATTCGAAGAATCCTCCCCAAC  CTCGGTCACTATCATACTAGAATACGATGATAATCT |
| Unigene6082 | 272 | 0 | 0% | TACTTTTTTTTTGGTCTTGATGAATTGTTGGGGTTTTGCTGGTAATGTGG  TAGGCTGAATTGTTGTTGTTTTTCAACGACTATGTCCATGGATTCTTCTT  CTTTTGAGGGAATAGCGGCTGACCCATCGAAATGCAGTAAGTTGAGTATG  GATGAGAAAAGAGAACTAGTATATGAATTATCGAAGCGATCACATGGTGC  CTCTGAAATGCTACAGTCATGGACTCGTCAGGAGATTTTGCAGATCTTAT  GTGCAGAGTTGGGAAAGGAAAG |
| Unigene9234 | 1292 | 0 | 0% | AACATGGTCAGATTTGAAGATGTTCGTGGAACATCCCTTACTGTGGTATT  GGGTTCTAAAATTCCTTCACCAGGAAATATTACTAGTTATGCCTTATGGC  ATCGGAAGGCACATGAGGACTTTCCAGTTAGATCTACTTGTATTCTGTCT  GTACCAAATACAAGGTTTGTTGTCAATGGACTTACTCCGGCAACTGAGTA  TCATTTCAAAGTTGTTTCCTTCAATGGTATGACAGAGTTGGATACAAGTA  AAGTTTCGATCTCAACAGCTAGTAGCGGGGATGAAGTCACAAACAGCTCA  GTAGTTGAAAGAAGTCAAAGCCCAACAACAAATTGTAGCAGCCTTTCTAA  TCCATCGTCGGTGGAAGATGAAACCAATAATATAATTCCAGACAGTGACC  AAAATGATGATCGCGAGGAAAATTATTCCAAATACTGCAAAGGCACTGAT  AAGATTGCTTCTGCTAATTTATCTGATAATGCTATTGATTGCATTGATGT  GGGTGAGAGAGCAACCCCAGCAGATGTAGTTTCTTTGTTGGAAGAGGAAC  GAGCAATTAATATAGATGGGTCCGTGCCTGATTCAGATGTTCTAAAGCTT  GAGAACAAGCACTCACCGGAGGGCCAGATGACCGAGGAGGTGAGCACTGA  TAATGGACTAGATACCCCAGTGCGGACAGGTATGGAATGTGTGCCGTACG  TGGGCAGCTCAGAAGCTGGTTTGCCAATCACTCCATGCAGGTTGGAAGTA  GTTATGGATGGGCAAGGAAGGAATGTGAGATCCAAATCAAGCAACAAAGA  TCAGGAAAATGGATTTGCAAAAGGAGACGAGCCCATGGTTGGTAGCACAT  CAAAGATAACTAGCGACAGACAGGACGAGGACTGTACAGCTAATCGTGTT  TCAGATATGGATTTTGAGCACTGTGTGAAGGTGATCAGGTGGTTAGAGTG  TGAGGGACACATTGAGAGGAACTTCAGGCAGAAGTTCTTGACTTGGTACA  GCTTGAGAGCAACTTCACAGGAGGTAAGGATCGTGAAGGTGTTTATCGAT  ACCTTTGTTGAAGATCCAGCAGCGCTCGCAGAGCAGCTTGTGGATACCTT  TTCAGATAGTATCACAAGCAAGAGATCATCAGTTGTGCCTGCTGGCTTCT  GCATGAAGCTCTGGCATTGATTTATAATGTCCTGTTATTTTGCTCAGATC  AGTTTCTCAGCATAACCCACTTAGGGTTACCATAACATAATTCTTTAGAC  ACCCCACTTAACATTGACTATCATCATCATCATCATCATCAA |
| CL3036.Contig2 | 1224 | 0 | 0% | AAGGGAATTCAGCTTTTAATGTTTACATATTTAATTTGCCTTCTTCCGAG  ATAAATTATCAATCTAATGCACTCGGTAGTACTGTAGCGGCCAATCATAG  CAGACAATACCATATATTTGCAGAGATGGAAGATGATGACTCTGAATATG  CAGATCGGCTGACGCTGAACAAAAATGTCAATTCTTCTGGGCTGCAGAAC  TTGTTCAGTGGTTCCAAATATAATAACAGTGCAAGTTATGCCAGTGAAGG  AAACTTACATCAATTTAGAGGTTATGGTAATTCTCAAGTTGGAAATCAAT  CTACTCGGGATATAGGTGTTCAGTTTGATATTGCTGAGCTTAAGAATTCA  TTAAATGCATTGTATTCTCCAGATGGAGGATCTCAAAAGCCTAAGAAAGC  AGGGAGGAAAAAGCGGAAGCTTGATCCTAACAATCCGGACACATCAGCTC  AATATCAGCAGCATGAAGAAGATGTAGAAATGCGCTTTAGATTTTATGAA  AGTGCTTCAGCAAGAAAGAGAACTGTCACAGCTGAAGAAAGAGAGCGAG  CAATTAATGCAGCCAAAGCATTTGAGCCAATTAATCCTTTCTGTAGAGTTG  TCCTGCGACCATCTTACTTATATAGAGGGTGTATTATGTATTTGCCATCC  TGCTTTGCTGAAAAGCATCTAAATGGGGTTTCAGGGTTCATTAAGCTACA  GCTTTCTGATGGGAAGCAATGGCCTGTTCGTTGCCTGTATAGAGGAGGCA  GAGCTAAGTTCAGTCAAGGATGGTATGAATTTACATTGGAGAACAATTTG  GGGGAAGGAGATGTTTGCGTCTTTGAGGTGCTTAGATCGAGGGAATTTGT  GCTGAAAGTTACTGTGTTCCGTGTCATGGAAGGTGCAGGATTCATGAACA  GATTGTCGCATTAGTAGTCAGTGAATTAAACTGGAATCTGCAGTGTATTT  TTATCTCTGGTTGCTAAAACCTCTTCGAGTAACCCTTTGTTTTCAGGTGA  TCTGTTACCAATCCAGTTTATTGTCCATAAACTTTGGTTGTACAACTTGG  TGACTTTAAGTGGTAGTTTAGGGGTTTCTATACAAAATTTTGTGGTTTGA  CTAGGGTTGTATATAGATTGTTCAGTCTAGTTGACGCTCAACCAAACTGG  AACAATGGGTTATAGTCATAATATCATTTTCGCTTTTAAGTTGGAAGATT  TCATTCTCACTCCATCTATGCATG |
| Unigene1789 | 1824 | 0 | 0% | CTGGCCGGTCCTCCTCGTCCTTCCCAATCTCCTCTTCTGCGAAATTCCTT  TTTCTGATTTAGCTGGAATTGAAGAGTGAAATCAAATGTGCCATCAAAAT  TCTTGTATGCATGTAGAAGAGAAGGCTGCTGGCGAAGAGAGTCTCTTGCT  TTTTTGCAAGCCTGTTGAATTATACAATATTCTGCGTTCCCGAGCTCGGG  AAAATCCTTCGTTTCTTCGAAGATGTTTAAGTTACAAAATACAAGCCAGG  CGCAAAAGGAGGTTGACAGCTGGAATTGTAGTTTTCAACTATAGGGACTA  CAGCAACATGCTACAAAAGACAGAAGTAACTGAAAACTTCTCTTGTCCGT  TTTGCTTGATGCAATGTGCAAGCTTCAAGGGTCTGCGGTATCACCTGTGC  TCATCACATGACTTATTCAACTTTGAGTTTTGGGTAGCTGAAGATTATCA  GGCAGTGAACATCTCCGTGAAAATTGATACACTGAGATCTGAGATTGTAG  CAGGTGGATCAGACCCACGACTGCAGACATTCTTCTTTTGTGCAAAGCCA  CGAAGACGTAGACCGAAAAACCTTACCCAAAAGCATGTCAACGTACAATT  CTTGGAGTTGGAATCGCCCAAATTACCCACGGAAGATCCACACCAAGAAA  TTACTGAGAAGGATGATGTGGAGAAGGCTTCCAAGTCACTTCCCAGTGAG  AAAAGGTTGCCGAATGGAAGGGACGTGACCAAAAACCATGAATTTGATAC  AGTGGAATGCATTGAACATGTAGCATCCAGTTTCAACATTCCTGGTGTTT  CAATTGCCATGGCTCAATCTTCTGTTGACCTTGAAAGTGGTAAATCAATA  GCTGGAAATGATGTGGCAGCCCCTGCTATGCAACATATTGTAAAATCTAG  AAAGTTAAGCACGGAACGGTCGGACCCTAGAAATCGGTCACTTCTACACA  AACGCCAGTTCTATCATTCTCATCGAGTTCAGCCTATGGCAATGGAACAA  GTGATGTCAGATCAAGACAGTGAGGATGAAGTTGATGATGACATTGCAGA  TTTTGAAGACCGAAGGATGCTTGATGATTTTGTTGATGTCAGCAAAGATG  AGAAGCAGCTGATGCATCTTTGGAACTCTTTTGTGAGAAAGCAACGGGTG  CTGGCAGATGGTCACGTTCCTTGGGCATGCGAAGCATTTTCCAAGCTTCA  TGGGAAGGAGCTGGTCCATTCTCCGGCTCTATTTTGGTGTTGGAGGCTAT  TCATGGTCAAACTTTGGAATCATGGCCTTCTTGATGGATGCACAATGAAC  AACTGTAATATGATACTTGAAAGATGTCAAGAAGAGGGATCAGATGTTGT  AAAAAGCTGAGGGGATGACTTACTGCTTTGGCTCTAAAGAGTATGACAGC  ATAAATAATCAAGTAACATCGATTTCCCCCTGTAACCCATTCATTGTGAC  TCTCTGTCTTAATTTAATTTAGGCCAGAAAAATTGTAGCATTTAGTTCAC  TTACAAAGAATGGAGCTGAGAGCTTCCTTCTTTGCTGTTGTAATCTTTAT  TTACACGGACATTTGATGTCATTTCCCTCTTAAGTTATTTAGGTACAAAA  CTTGGAGAAAAGAAAATGACTCGCTTCAATATAGCTTGTTGGAAACCTCA  ACCTCCTTAAAATTTGTAGAGCTCATGAACAGTTAAATAGCAAATTAAGT  ACCCAGAATAGATCCAATTTCTGGCTACTTGGCCCCTAGCTATTTCACTG  TGAAAGAAAATCTGTACCGTTCCATCAATATAGCTGAAACATAAGAACAG  TCTTTTCAAACTGTAATTCGCCAG |
| Unigene6914 | 2362 | 0 | 0% | GGTAAATTTCCAGTTTCTGATACTAGTTTAAAAAATATTCCTCAAAATTC  CCTCTGCGATTTTTCTAGGGTTTCTACCACTACAAGGTGCTCGAGAGAAG  GTTTCGTCAGCGATGAAATCGTTTATCGCTGGTGCGTAGCGAGAATGCCG  GGCATACCTTTAGTGGTCCGTGAAAGCTCCTCATACACTAGAAGCGCAGA  ACAGATGTGCCGTGAAGATTCTCGAGTGCATTTATCTGCAGAAGAGGAAA  TTGCTGCCGAAGAGAGTCTTTCGATCTATTGCAAACCTGTGGAACTTTAC  AACATTCTTCAGCGACGTGCTATAAGAAATCCGTCATTTCTTCAAAGATG  TTTGCTTTACCAAATCCAGGAGAAGCGCAGGAGAAGAATACAAATGACAA  TTTCCCTGTCAGGAACTGTGAATGAAGGTATACAAGCTCAGTCTCCATTC  CCCTTATACATTTTGTTGGCAAGACTAGTTTCTGACGTTTCAGTTGTCCA  GCATTCTGCAGTATATCGCTTCAGTCGAGCTTGTATCTTGACCAATTTCA  CCGGAGTTGAGGGCAGTACTCAGGTTCATGCAACTTTTATTCTACCTGAG  ATTAATAAGCTGTCATGGAAGGCCAAATCTGGTTCACTTGCAATCTTGCT  TGTCAACTTTGCTGGAACCTCAAATTCTTTGTGTGGAACTGATTTAACCA  GGGGTCGTTTCGGTATCACATCATTTTCATCAAATGATGGAGGACACTGT  CTTTTGGGCAAAATTCCGTTGGAATCTCTCTGTGTGATGTGGGAGAAGTC  CCCAAATTTGAGTCTGGGACAGAGAGTTGAGGTGACTTGTCCCGTTGACA  TGCATTCTTGCTTCTTAAAGTTGAGTTGTTTGAATAAGGACAAATGTCTC  TCAATTCAGATGCCGGTTAATTCTGGAGCCACGAGTACGACACAGCAGGT  GCATGTCACCATTTTGGCAGAAGAGTTTGGGGCAAAGGAAAAATCTCCCT  ACAATTCATACACATGTAGTGATGTTTCTTCGTCGTCTCTATCTCGCATT  ATTCGGTTAAGGGCTGGAAATGTTGTCTTCAACTATAGATATTACAACAA  CAAATTGCAAAGAAGTGAAGTAACTGAAGACTTTTCTTGTCCATTCTGCT  TGGTGAAATGTGGAAGCTTTAAGGGTATGAGATATCACCTGCCCTCATCA  CACGATCTCTTCAACTTTGAATTTTGGGTTACTGAAGAATATCAAGCTGT  AAATGTATCCGTGAAACCTGATACCTGGAGATCTGAGGTTGTTGCAGATG  GTGTTGATCCCAAGCAGCAAACATTCTTCTTTTGCTCAAGGAGACGAAGT  TACAAGAGATCAAAGAGCCTAGTTCAACGTTCAAAGCATGTACATCCACT  TGAGGCAAACTTGCCTTCAGGAGTCTGTGAGCTTCTTGACAAGGCTGACG  GGGGAAATGGTTTTCAAAATGTCAGCACTGGTGTTGCCGAGTGTACTCAG  CTTGTTCCTTCTAGCTTTAACATACCTGGGCTTTCAAGTGCTGTTGCACA  GTCTTACACTGATCCTGAATGCATTCAATCAGTATCTGGAAACAATCTTG  CTCCACCTGCTGTGCTACAGTTTGCGAAGACTAGAAAGTTATCTATTGAA  CGTTCTGACCCAAGAAACCGGACACTCCTTCAGAAGAGACAGTTTTTTCA  CTCACATAGAGCTCAGCCAATGGCTATTGAGCAAGTAATGTCGGATCGGG  ATAGCGAGGATGAAGTTGATGATGATGTTGCAGATTTGGAAGATCGAAGG  ATGCTTGATGATTTCGTGGATGTGACCAAAGATGAGAAGCAAATGATGCA  CTTGTGGAATTCATTTGTGAGGAAGCAACGGGTGCTGGCTGATGGTCATA  TTCCCTGGGCATGTGAAGCATTTACAAAATTGCATGGACCTGACTTGGTG  CAAGCCCCTTCTCTGATTTGGTGTTGGAGATTATTTATGATCAAACTGTG  GAACCATGGTCTTCTTGATGCTCGAACCATGAACAATTGTAATATTATTC  TCGAACAAATTGAAAAGCAGGACTCAGATCCAATGAAAAGTTGAAGAGAC  GTTTTTCTTTCTAAAATGATTAGTTACCATAACTAATCATTACTCTTTGA  GTCACCACAGTCCTCTGGAGTGTTCGATTTTGTCGGGTCTTCCTGCTGTT  CCCTACCAGGTTTAAAGGCCCTCCAATATTCTTCCTCTTATTACTCTGTT  GAACCATGTTAATGTTTAAAGATGTTTTTTCATACACGCAATAAGTTCTT  TTAAAGTACAATTATCAGAATTAGAGATAAAAGAGATCTACGGCAAGACT  GTAATTTATTTT |
| Unigene11831 | 1322 | 0 | 0% | ACAAGGGTTTCACTTTTCTAATCTTATTGTCAAAAACAAGATAATCAGTT  TACCTCTTCTACACCAAGATTGGATGAAAACAAATAAGCAATTATCAAAT  AGGGGGAAAAAAAAACAACAAAAAGAAAGAAAATCTACTTACTTAAACCA  TAGTAATATAAATCCACCTTAAATTCATAAACATCATCTCTTTTAATGGC  AGCTAAGAGAGTGGGGGCAAACAAAAGAATACAGAGTTTTGAATACTGAC  ACTTGTTGCTCACCACCATTCCAAAATCAAAATGAACCACCATTAGCAGC  ACAGTGGCGCAGACAGGGAAATTCACATCCCGTTGTAGTTGCTCCAACCC  AACTCTGCCATGGACTACTACAGGAACTGCCTCTTTCTCTGAAACATGCA  TCTTCAATTCTCTTACCATCCCGCTGCTCTTGATTTCTACAAACTCCTGT  AGTCTATAACAGTGACTTCATCTTCTGGTGCATCTAGATCTTGATAACTT  CTTATTCGTCGAGGATCCTGTCTAAAGGCTGGAGACAATAAAATTGGGCC  TGCTCCACTTAGCTGGGGCCCAGAACGCCCATTTCGACCACCACCTTCAA  AAGGGGGAGGGCCACCTTGCTCCCTGAACATCCGCATAGCAACTTCAGGG  GGTGCTGGAACAAAGGGCCCCAATGGACCAGCACCAGGAACAGGCATCA  ATACAGGTGGAGGAGGTATATCTGAGGGAAAAGGAGGACCAACAGGTAT  GCCTTGTCCGCCAAAAGCGTCAAACATTGGATCATCACGATTTACCCCATCA  GGACCATCATTATTCGATGGGAAATCACTTGATTGCGCATTGTCAGACCT  ATCATATCTATCACCACCATTAGGACGATAGTCACGTTCTCTACGGTTCC  CACGTTCATCTTTCAATCGGTTTTCTGACCCAAGCCTGCGTCTCAATGGC  TTCTCCCTCTGTAATGGCTGCTGCATCACAGGAGTCCCACCAGGTGCATC  TTCGTCATTCATGTAGTTTTGGTAATACAGCTCTTCACGCACTTTTGAAG  TCAATTCCATCACAAGTTCTGGGTGTTTCAGTTTAAGATGCTTGTGCACA  AACTCAGCAGCATGAAAAAGCTTTGTGCAACCTTTGGCTCCACAACCATA  TTTCCAGCCATATTTTTCATCCCTTATCTTCCGGACAAAGGGATCCAAGG  CATCAACAGCAGTCGCATCAATCTTCTCTTTAGCAGTCATAATTTCCAAG  GGATCTTGACTCCTCAATCTTTCTTGCCAACGGGACTCAAGTTTCTTTTC  CCACTCAACCCCATTACTGGCC |
| Unigene7433 | 1491 | 0 | 0% | CGGGGGATGAAAGGTGAAGGCAAAGAGAGTTGGTGAGCGGAAAACTCT  CTCAATTAAAAACAATAAAAAATAAAAAAAAAATAAAAAAGGCCATTTCTTC  ATTTGGTGGTAGTAGGCCAAGCCCTAGGGTTGGACCGTAAGCGAGTGATG  GCTCAGGTCATTAACATTGCCGTCGATTCCCTCGACCGACGCCGTGACCG  CAACGAGAAATCCGACGATAACGACGACAATCCTCCCCAATCATCACCGC  CTCCACCTCCAGCTCCTCCGGCTACTTCTAGAAGGCGCGATAGAGACTCA  CGTGAACGGCGTGAGGAGAGGGACCTCGACCGACCTCCCAACCGGCGAG  GTCCTGATTATTACGAGCGGAATCGGTCTCCTCCGATGAGGCGGAGGCCGA  CGGTGAGTCCTCCACCTCCTGCTATGTCCTACCGCGATAGAAGGCACTCA  CCTCCTCCTCTTCGTAGGTCCCCTCCCTATAAGCGTTCCAGGAGGGAGGA  TGGAACCTACGAGCCTAGGAGAGGTGCTAGTCCGCGTCCTCCTTTTGGAC  CTCCCGATAGAAGGTTTGGGTACGATTATGCTGGTGGATATGAGCGTGAA  ATGGGGGGCAGACCTGGCTATGCTGAAGAAAGGCCTCATGGCCGGTACAT  TGGTCGCCCATCTGGTGGCTATCAGAGTGGTGCTTCTGAATGGGATTCAG  GCCGCGGTGGTTACAGTGATGCTTCTAACACAGGCAGTAATCAAAGAGAA  GGCTTGATGTCATACAAGCAATTTATTCAGGAGCTTGAGGATGATATACT  ACCGGCTGAAGCTGAACGCAGATATCAAGAATACAAGTCAGAGTATATTT  CAACTCAGAAGAGAGTGTTTTTTGATGCTCATAAAGATGAGGAATGGTTG  AGAGATAAATATCATCCGACAAACTTGCTTGCTGTCATTGAAAGGAGGAA  TGAACTTGCACGAAAGGTTTCAAAGGACTTTTTGCTAGATTTGCAGAGTG  GGACATTGGACTTAGGTCCTAATGTCAATTCCTCATCTTCAAATAAATCT  GGACAGACTAGTGATCCAAATTCAGATGAAGAAGTAGACGCTGGTGGCAA  AAGAAGGCGGCATGGTCGAGGACCTGTTAAAGAAACTGATCTTATCGCAG  CTGCTCCTAAGGCGCATCCAATCAGTTCAGAACCCAGAAGAATTCAAATT  GATGTTGAACAAGCACAGGCCCTTGTACGTAAACTTGATTCTGAAAAGGG  AATAGAGGAAAATATATTGTGTGGATCTGATAGTGATAAAATGAATCGGG  AGAAATCCCATGGTGGTTCTAGTGGCCCAGTTATTATCATAAGAGGTTTG  ACCTCTGTCAAGGGCCTGGAGGGAGTTGAGCTGTTGGATACGCTTATCAC  TTATCTGTGGCGCATCCATGGAGTGGATTATTATGGGATGATTGAAACAA  GTGAGGCCAAGGGTCTCAGGCATGTCAGAGCAGAGGGAAAG |
| Unigene13940 | 1135 | 0 | 0% | GAGGATCATACTGGTTTCCTGCAGATGATAAAATTGCAGGGGGGATAAAC  AAAGATGGTTGAAAAGGTACAGATGGTTCCCAATCATTTGATGATATTTT  TGTTTTATAGTCAGCAAAAAACAGTGTTTTATCTGGCACAGAACCAGAGA  AGGGCGAAGAGCATCTCAGCGATGAAGCTGATCTAGATGTCCGACATTCT  CTATCACTTTGTAATAGCTTCTGAGTGCCAAGGGGACTTGAATTTAAAGA  GGAAGAATTAAGAGGCAACAGTGATCCTGTCCAGGTTGAGACACGATGAC  ACGTTAAATTGCCAGCCATTGGACCAGAAGTACTCAGGCTTGAATTCAGA  TGGCCCAAAACTGGTGAAGAATAATTGTTGTGCATATATTGACTACGATT  ACCACTCCGCTCCTCAAGAATGCTCGAATAAGAAGAGGGATTCTCACTGT  TATAATCGTTAGATGGAATAGCTGAACCACTTGAAGAAGATCTATATTCA  GGAAGCAAGCTGCCCCTGGCTATAGGAAAACTGCCCCTATATATTGGTGA  GGAAACAGGGTTGCGGTTCTGAAGTGATGAGAGATTGTGGTTTTCCTGCA  ACTGAAGCAAACTTTTGTTTTCTCCCTGTTCCTGTGGCAGGATCATTTCT  TCATTAACCAGATCTTCTCTTTTCACAGTTGCAGCAGCTACATGTTCCAC  ATGCTGCTTGGTAGTATTATTCACATCATCCTTCACATGAAGAAACTTAC  AGGAATTCCCTCTGATACACCATCCTTTAGCAAAGAAGTTGCAGATAGCC  GCTGGACGTTTGTTTGTATTGTCATTCTCAGCACCAGCTAACAAACTTCT  AGTCCTTATAACAGGTAAGCACAATCTTGATGCCAGGCTCCTACTATCAT  CCCCTAGATGCAAGTCCATCTGCGTATTTTCTTCCTCAGAACCCTGAATA  ACTACAGTCCCCTCATCAACATGATCCCGTGTTAGCTTAGCTACAACCAC  ATCTTTTCTAGTAACATCACATGGACTGGTGGCATCAGCATCCATAGTCT  CACTAGTATGCAGTTCATTTTGATCCACAACGGTTTTCACATTCCGAAGG  GCTTTGGCATCTTTAGTAGAAATCTCATATTCTTG |
| Unigene5288 | 866 | 0 | 0% | GAACCTCAATTTTAAGCAAGTTAATTTAGCCTCAAACAAACTTCCATGAC  TTCACCAGTTACTAGGATGTATAGGAAACTGTACAAAGACACAAAAAAAA  TCAGCCAAGCATCTCAAGATTTGCCATACTTATCAACATATCCCTTAACA  AGCTTGGCAATTTTTGGTTGGGATGAAGAAAGATAATGCTTAGCTGATTC  CACGGTGGTTGGAATTTGGTGGGGCTGTAATGTGCTAAGAACCTTGTCAA  CTGTCTTTTTGACTATTGTCTTATGCGCATCCTTGCTAAGATGACCCTCA  CGCCAAGATGGTTTCAATGATTCCTTAACCAAATCTATAAGATCAGCACG  TAGATGTCTCATTGGTTTCGACTCTTTGCATACATCCTCCATCTTTTGCT  CATCCATTTCATTGTTCTGTCTCACTCTGTCTACTTTCATGTCCTTATTG  TGTCTAGACCCATCATTTTTATGTCTGTCTACTTTCATGTCCTTATTGTG  TCCAGACCCATCATTTTCATGTCTTAAAATAACTTTGGCATGAGTCGAAA  CTGAGAGTTCTTCTCCTTTCGGCATGGTCCCATTTTGGCCATCACCTACA  GAAGTTTCCACTGCCTCTGCTCCAGCTGTATGTGAATCTTTCCCTGGTGT  TTGGCTATCATTTTCCAACACATTCTCATTGCTTGCACCATGAAAGGATG  CAGAACTTTTATCACCATTGCATTCTGGAGCAATGGTCCTAGAGAAAGTA  GAATCACCATATATTTGCTGATACGTTGTATTTAGATTGGATGCCCCCAC  ACTAGCGAAGGAAAATTTAGAAGATAAGTCTCTTGTGTTAGGCAAGTCAA  TGCTATCACGAAGAGG |
| CL67.Contig1 | 2080 | 0 | 0% | AAACAAAGAGAGGAGAGAGAGAGAGAGAGCGAGTAGTGAGTGGGGTTGG  GTGGTAGAATAAAAATATTAAAAACAAGGACTCTCTAATCGCAACTTTCCT  CTTCTGTTAGGGATTGCAGAGAAAAAACTAGTAGACTAGCGCAGCACCACC  ACCACAACAACCTTAGCAGCCACACAATCACACACACACACACACATCAC  CACCTCCTCCACCAACCTCGTTTTTGAAAAATGGATTGATCGTTTTGCT  ATTTTCAACGGCTTTTCTTTTCGTTACATCAACAGCATTTCCGCCATGGA  AAACACTCAATCAGTTGCTATGCTTATGGACTCTACCACCTCTAAGATAC  AGCAACTTCAGAAAGCATTTGCTGAACTTGAAAGTCACCGGGCCATAACT  CTTAATATGAAATGGAAAGAACTTGAGGAGCATTTCCACGGGCTTGAGAA  GTCATTGAAGAGAAGGTTTCATGAGCTGGAAGACCAAGAAAAGGAGTTTG  AAACCAAGACAAGGAAAGCCCGAGAAATGTTACAGAAGCGTGAAGCTGCA  GTTGTGGCCAAGGAACAAGCCTCATTGGACAAGCTCCAGAAGAAGAGAGAT  GCTGCTGTATTTGCTATTACAGCTGCTCTAGAGAAGCAGAGGAAGGTATCACC  TGTAGAGCCTGCTGTCCTTGGCAATGAGAGTGAGAGTGGGGGGGCTACTGTT  GAAGACAAACTGCCTGATTCCATGTCTGTTGAAAGTAATTTGGA  AAGCATCAAAACCTCCTCTGAGAACGGAAGTGTTGAGCTGAAGGTATATC  CCCAATTATTCAAATTATGCGAAGAGATGAACGCAGAAGGTTTGCATAAA  TTCATATCAGACAACCGTAAGAACCTTGCTGCTCTGAAGGAAGAAATTCC  ATTTGCACTAAAGGCTGCAGTAGACCCGTCCCGTTTGGTATTGGACTCTC  TGGAAGGTTTTTACCACATGGAAATGCCAAATGTGGATGGAAAGAAAGAT  TCAAACTTACTGGGTCTTCGCAGAACCTGTATTATGCTGATGGAATGTCT  TTGCATTTTGCTTGCCAATCTGGATGCGGATTCTCTTTCTGCTGTAATCT  CAGAAGATATTAAGGAACAGGCAAAGGCAATTGCTGAGGAGTGGAAGCCA  AAGTTGGATGCCCTTGATGTGGATGATAGCAATGGGAATTCCTTGGAGGC  TCATGCATTCCTGCAACTTCTGGCCACTTTTGGCATTGCTTCTGATTTTA  ATGAGGAAGAGTTATCCAGGCTGATACCAATGGTCTCTCGTCGTCGCCAA  ACGGCCGATTTATGTCGCTCCCTTGGGTTGTCCGAAAAAATGCCAGGTAT  AATTGATGTTCTGGTGAATAGTGGAAGACAAATTGATGCGGTTAACCTGG  CATTTGCATTTGAGCTTACTGAGCTGTTTTCACCTGTGCCTTTACTAAAA  TCCTACTTGAAGGAGGCAAGAAAGGCTTCTTCCCCGGTTAAGGTTGGGAA  TACCTTTCCCAATGCAGAGAATGAGGTCAATGAACGTGAGCTGGCTGCTC  TTAAAGCGGTGATTAAATGCATTGAAGAGCATAGCCTCGAGGAGCAGTAT  CCAGTAGATCCACTCCAAAAACGCGTTCTCCAGCTCGAGAAGGCAAAGGC  AGACAAGAAAAGGGCAACCGAGGCTTCAAAGCCTCAACCGAAGAGACC  CCGTGCCAATGGTGCTGGATATGTGCCGCCACGAGTGACCAACGTTGCTGCA  GACAAGACATTCTATCCTACTAGAGTTGCCGATAGGTATCCGCAGTACGT  CTACGAAAGACCTCCATATGTTTACACCGGGCCAGCTGACAACCACGGAC  CCCCCTCAATTCTGGGTGCTGCTACTTACAGCTTCACTCCCAGTCATGGA  AACTACTTTGGAAATGGCTACCAGTACCAGACCCCTTATCTTCACTAATT  TGGTTAAAAAAAGGTGACTATGATCCTCGCAACTTAACTGTTAGCCTGTT  AACCACTGAGTTCCCTGGACCTTGCAGTGTATGAATGAATTTAGTATTTT  TAAAAAAAAAAACCCAAAAAAAAAAAAAAA |
| CL67.Contig2 | 2142 | 0 | 0% | AAACAAAGAGAGGAGAGAGAGAGAGAGAGCGAGTAGTGAGTGGGGTTG  GGTGGTAGAATAAAAATATTAAAAACAAGGACTCTCTAATCGCAACTTTC  CTCTTCTGTTAGGGATTGCAGAGAAAAAACTAGTAGACTAGCGCAGCACC  ACCACCACAACCTTAGCAGCCACACAATCACACACACACACACACATCAC  CACCTCCTCCACCAACCTCGTTTTTGAAAAATGGATTGATCGTTTTGCTATT  TTCAACTGCTTTTCTTTTCGTTACATCAACAGGCTTTTAGTGTTTTTGTT  GCTCTAATTTCTTCGATCTCGTAGCCGAATATCTTAGTTGGTGATTCGAG  CTAGCTGATTTACTGCATTTCCGCCATGGAAAACACTCAATCAGTTGCTA  TGCTTATGGACTCTACCACCTCTAAGATACAGCAACTTCAGAAAGCATTT  GCTGAACTTGAAAGTCACCGGGCCATAACACTTAATATGAAATGGAAAGA  ACTTGAGGAGCATTTCCACGGGCTTGAGAAGTCATTGAAGAGAAGGTTTC  ATGAGCTGGAAGACCAAGAAAAGGAGTTTGAAACCAAGACAAGGAAAG  CCCGAGAAATGTTACAGAAGCGTGAAGCTGCAGTTGTGGCCAAGGAACA  AGCCTCATTGGACAAGCTCCAGAAGAAGAGAGATGCTGCTGTATTTGCTA  TTACAGCTGCTCTAGAGAAGCAGAGGAAGGTATCACCTGTAGAGCCTGC  TGTCCTTGGCAATGAGAGTGAGAGTGGGGGGGCTACTGTTGAAGACAAA  CTGCCTGATTCCATGTCTGTTGAAAGTAATTTGGAAAGCATCAAAACCTCC  TCTGAGAACGGAAGTGTTGAGCTGAAGGTATATCCCCAATTATTCAAATTATGC  GAAGAGATGAACGCAGAAGGTTTGCATAAATTCATATCAGACAACCGTAA  GAACCTTGCTGCTCTTAAGGAAGAAATTCCATTTGCACTAAAGGCTGCAG  TAGACCCGTCCCGTTTGGTATTGGACTCTCTGGAAGGTTTTTACCACATG  GAAATGCCAAATGTGGATGGAAAGAAAGATTCAAACTTACTGGGTCTTCG  CAGAACCTGTATTATGCTGATGGAATGTCTTTGCATTTTGCTTGCCAATC  TGGATGCGGATTCTCTTTCTGCTGTAATCTCAGAAGATATTAAGGAACAG  GCAAAGGCAATTGCTGAGGAGTGGAAGCCAAAGTTGGATGCCCTTGATGT  GGATGATAGCAATGGGAATTCCTTGGAGGCTCATGCATTCCTGCAACTTC  TGGCCACTTTTGGCATTGCTTCTGATTTTAATGAGGAAGAGTTATCCAGG  CTGATACCAATGGTCTCTCGTCGTCGCCAAACGGCCGATTTATGTCGCTC  CCTTGGGTTGTCCGAAAAAATGCCAGGTATAATTGATGTTCTGGTGAATA  GTGGAAGACAAATTGATGCGGTTAACCTGGCATTTGCATTTGAGCTTACT  GAGCTGTTTTCACCTGTGCCTTTACTAAAATCCTACTTGAAGGAGGCAAG  AAAGGCTTCTTCCCCGGTTAAGGTTGGGAATACCTTTCCCAATGCAGAGA  ATGAGGTCAATGAACGTGAGCTGGCTGCTCTTAAAGCGGTGATTAAATGC  ATTGAAGAGCATAGCCTCGAGGAGCAGTATCCAGTAGATCCACTCCAAAA  ACGCGTTCTCCAGCTCGAGAAGGCAAAGGCAGACAAGAAAAGGGCAACC  GAGGCTTCAAAGCCTCAACCGAAGAGACCCCGTGCCAATGGTGCTGGATAT  GTGCCGCCACGAGTGACCAACGTTGCTGCAGACAAGACATTCTATCCTAC  TAGAGTTGCCGATAGGTATCCGCAGTACGTCTACGAAAGACCTCCATATG  TTTACACCGGGCCAGCTGACAACCACGGACCCCCCTCAATTCTGGGTGCT  GCTACTTACAGCTTCACTCCCAGTCATGGAAACTACTTTGGAAATGGCTA  CCAGTACCAGACCCCTTATCTTCACTAATTTGGTTAAAAAAAGGTGACTA  TGATCCTCGCAACTTAACTGTTAGCCTGTTAACCACTGAGTTCCCTGGAC  CTTGCAGTGTATGAATGAATTTAGTATTTTAAAAAAAAAAAA |
| Unigene3715 | 867 | 0 | 0% | GTTCAACGAGCCCACAAACAATTTGTACTCAACTGCACCTAGGCGTTCTC  GTTCCCCGTCAGCATATCGAACTTGGATAGGACCTGCTCCCCCAGGAAGA  GTGTGTTGATTGTGCAAGGCTCTAATGGCCCTATCAGCTTCTTCTGAAGT  AGCATATTTTATAAAACAACAACCTTGTTGTTGTCCTGTCCTCTTATCTT  TAATCAAAGCAACCTCCACTACACGTCCATGGTCATCAAACAAAGGCCGA  ATATCATCTTCAGTAGCAGTCTTGGGTACCGACCCCACAAAAAGCTTGGC  AAAACTGCCACCATCACTGCCATCAGGAGAAGGCCCACCGCGGCCCGAAA  AACCTCGTTTCTGGCCCGAAAGCTGTGGCACTGGCGGTGGCGGCATCGGA  TAGCTCGGTCCAAACCCTCCTCCTCCTCCTCCCATCGGCCGAAACATTCC  TCCTCCTCCGGAACCACTCGTTGGCCTAAACCCTCCTCCAGGTCCTCCAA  CTCCAGCAGGCGGCGGCGGACTATCGAAGGGCCTGTGACCTCCTCCCGCA  CCGGCGACGCGGAATTCGCTAGGGCTACTACGACGGCGATCGTATTGGTG  GTAGGAGGAGTCTTCATTCGTAGCGTTGTTGTTGTTGAAACGGCTTATCG  GAGTGTCGGAGAACCGAGAAGACCGGTTGTTGTGACGGTTGTTGTTATAT  TGATGAGAGTCCTGGTTGGGAGAATCGAAGTTGTTGTTGTTGTTGCCGCC  ATAAAAACGGTCTCCTCTGTGCCTCTCCATTAGAAATTAATAAACACTAC  TCTCTAATAAATTTAAAAAACAATAATAATAATAATAATAACAAATCAAA  CCAGTAATAAAAAAAAA |
| Unigene12093 | 1981 | 0 | 0% | GGGCGAAAAGTGTTCGGTGGCCTCATATTCTTATACTTGTAAATTGCATA  TGAAAAATTAGGGAGAGAAATCTACTACACAAGATGCCCAACAATCTACA  TAGGAGTTTATTACTACGGTTAAAGCTAAGCACATGATTATAGCAAAATT  AATTTTATGTTACAAAATGTGAAATTAAAAAGGGGTAAAAGGAACAATAT  TTTTGCTACCAAGGCATCTATTGCCTTGCATTTTAAATGCTACAAAGGAT  TGATCCACCAAGCCAGTCACTGCCGAGCAGTAAATAAAACCTACAAATAA  TATGTGAGGAAGGAAGATTTTGGAGCAAAACTGTCTTCAATATTTCAAGT  TCAAAGCAGTTGTTCCCGCTTGTCCTTGCCAAAAAGTTTGTTGCACAGTT  CTCTTGATTCAGCATTTCTTGGCCTCTGGTTTGATTTTTTACTTCAAGTA  CCTGATGGTTTATTCTTCCACATCCATTCCTGAGTAGCCTGAAGCCCCTG  TTGGAAGCGTGCAGGATCATTAACCGCACCGGGCAATTGACCATAACCAA  TTTCCTGAGGATTGTGTTGACCTCTGCCTCCAGAAGCTTGATACGACAAA  GGAAAAGGCTGCTGCTGCTGCTGACGGAGCTGTGTTTGAAGCTGCACCTG  TTGTGTTTGTGGAACTTGCTGTGCAGGCATAACTTTGGGGTGTGACTGGG  TTTGAAGTTGTTGAACTGGTGGTTTCTGTTGCTGCTGCTGTTGTTCAAAT  GATGTCAACTCTTCAGGTTTCTCCCACTTGCTTTCACCAGTTATGTTGTT  ATAATAGTACTTGTATCCATCGGGGGAGGTGTGTTCGGTCCAATTACATT  TAACAGAAGCTGCAGTCTGACTTATTACAGGTACAGCAGAAACAGCAGAC  GTAGATGATGGAACCTCTGCAGCTGGCGTAGAAGCAGGAGCACTGGCAGC  ACTCTGGGGGGAAATACCAGGCCACTGAGACTGCTGTTTACTAGCCTGAG  AACTCTGCTGTAATGCTAAGCTTTGATTTGACGGTTGCATCAGCTGCAAT  TGTTGTTGCAGCTGAGAGAATGCTTGCTGTGAAGATTGAAAACTTGCCTG  CAGAGTTTGTGTCTGCTGTGATAGCATCTGCGCTAGCTGAGACGGGGACT  GCTGAAGAGGTTGAAGCAGTTGCTGCTGGACAGGAGCAGATAACTGCTGT  TGAGTGGTTGGAGCAGACAGAGAACTGGACTGCAGGTTAATATTTGGAGG  GGTTAGAGCTGTAGCAGATGAGGGGATCTGCTGAATGTGGGATTGAGAAG  CAGACAATGGTCCACTCATGGCAGGTAAATGTTGTGATGGCAGTGCTTGA  CCGAAAGGTGTTTGACTGGGAATCTGTAGCTGACCGACAGGGGGAGTTTG  TGTCTGTGAGTAAGGTGGAGCCTGAGGATGGAGTTGAAGGGAAGGAGGCA  TATGCTGAGGTGATTGACGAGGCTTCTGCAGTGGCGATATATGCTGACCA  TGGGATAGGACTTGTGACAATGGTGGGTTAAAACCCTGTTGTGTACCAGA  TGAAGTTGAGGAAGAAATTGGAAGCCCTGGAAGAGGACCATCAGCAGGA  CCACCCATATTTGAAGGTATCGCTAGATCACCCAACCTAGGAAGCAACTGA  TTCCCAAAACCACGAATACTAGGATTAGGCCCCATATTCTGAGGACTCAT  AGGATGCCAGGCATGAGGTGGTATTCGATCACCTATAGGGTCACCAAAAC  TGGGTGCTGGTCTAGGCCCTGGTGGCTGGAACCGGGGACCAAAACCTGGA  CCTCCAAACGAAGGATTCCTTGAATCTCCAGGTCTGGGCCTCTTAGGATC  AGCAAACCGAACAGTCAATGGTTGATCACAACCTCTCATTGTATAAATTC  CATTCAGAGCATTGATAGCTGCCAAAGCCATATCTCTATGAGAGTATTTA  ACAAATCCACATCCCCGACTCTGCTTCAATT |
| Unigene8904 | 828 | 0 | 0% | GGGCAGCAGACAATGCAAAAACCAACTCTGGGTTTTTAAGCAGTACAGCA  AGCAACTGAAGATCTGGTTTAGCTGAGCTGGGACTGGCACTGGCACCGGC  ACTGGCACTGTTACTGATACTGGCACTAGCATTGCTCATCTGAGATGGAA  TAGGAATTGGAGTAGCTGATGTGTGTACAATATCATTATTAGTGGCAACT  TGTGTATCTCCACTATCAGCAGCATCAGGAGGCTGTTCAGTTGGTATTTG  AGGTGTCAAAGAATCATCATGGTCCATTTCACGGTCCCATGGCTCCTTGG  GATTGGATGGTATCTCCTGGATTGTACGATAAACGGTTTCCCTCTCCCGT  TGGTTTCTTTTTTTCTGAGCTTCAACCTCTTTGCTATTCTCACCCGCACT  GACTCTCCAAATGTCGTTCAGTTTCACTTCTGGCGGTGTCCGCCATGGAA  TTAAGACCATCTTGCCCTTTTCCTGCAGATGCTCCTTCGAACCCATTTTC  TGCTTATCAAGAGAAGCTTCCAGCCCACTAGCAACTTCTGCGGAAGGTAT  TACTGATTTCTTTTGTTCCTCAGTTTTAGGCAGAATATTAACTTTAGATA  TTGGTGGTGAGATGCTGGCTGGAATGGCTGTAGATTTGTTTAAAGCTTCA  GCCTTTGCCTCATTACTTCCATTAGATGAACTAGTTTTTGCATGTTTACT  CTGCATATACTGAGCTCGCATTTTTGCTTTTTGGATATCATCGGCAGACA  TTGGACGACCTTGATTCATCGCAGCAGCTCTGGCTGCCACTGCCTGCGTG  CTTCTGCCAGCCAGTTTTTGGCCAGGCT |
| Unigene10275 | 587 | 0 | 0% | AAAATTAGCGGAGCCATAGAAAAACAAATTCGGGTCACACTATCTTTCCC  GCTCCCTCCTTCCCTCCCTCCGGTCAAATTAGGGTTTAGGGTTTTTTTTG  TCCCCTTAATTACAGTGTTTGTCTCTCAATCATCACCGTTGAACTAGTTT  TCAGCGACAATGATGTACGGTGACCCTCAACAGCATCAACATCAACAACA  TCAGCAGCCGCCAGTTGTTGGCGGCGGAGGCGGGGACCACTTCCACCGAG  GGCCACCGCCACCGCAACCGCCAATGATGCGACAGCCTTCCGCTTCTTCC  ACTACTCTGAATCCAGGCGATTATCACCACCCCTCCGCTAATCCTCCTTA  TGACGCTCATGCTGACAGTTTTACTGCAAAAAGAATGAGAAAGCTCACCC  AAAGGAGGGCAGTTGATTATACTAGCACTGTTGTGCGGTATATGCAGATC  CGAACATGGCAGCGGGATTCAAGGGACAGAACAGTATTGCAACCTACCCC  AGCAGCTGCAATTGATATGTTACCGACAGTTGCATATGCTGATAACCCAT  CCACCAGCTTTGCTGCAAAGTTTGTTCATACATCCCT |
| Unigene13338 | 749 | 0 | 0% | CAGGTTGAAATTCCTAATGCACATGATAACAGTGTGTGGGACCTTGCATG  GCATCCCATTGGATATCTTCTCTGCAGTGGTAGCAACGACCATACCACAA  AGTTTTGGTGTCGAAACAGGCCAGGAGATACTGCACGTGATAAATTTAAT  ATGGGTCAGAACCAAGGTTATGGTGAGCAAAATGCTGCTCTCGCTGGTCG  GATGCCTGGCAATTACCCAGTTCCTGAAACTCCAACAACTCCAGGACCAT  TTGCTCCAGGACTGAGTCGCAATGAAGGAACCATTCCTGGTGTTGGAGTG  GCAATGCCATTTGATTCATCAGCTCAAGGAGAGCAAAAGCAGCCTCTTCC  AGTTTCAATGCCTTTAGGAGCACCTCCTCTCCCACCGGGTCCGCATCCCT  CTCTTCTTGCTGCCAATCAGCAGCAGGGATATCAACAAAATTCTCAGATG  CCAATGCAACAACACCAGGGACACCCGCAGCAAATGCCCCCAATGCCAAT  GCCACCTCCAAATATGCCACAGCTGCAGCCTCCATCTCATTTACCCTTGC  TCCCACATCCTCATATATCTCGCCCCCCACCACAAATGCCCCAACTTGGC  ATGCCTCCCCCAATCCCATCTTCAATGCCAGGGTCTATGCCTGTCCCTTC  ATCAGTTCCAATGCCAATGCCTGGTCCAGTGGGGATGCAAGGCACAATGA  ATCAGATGGCTCCACCAATGCCTCAAGGCCATTATATGGGCATGAACCA |
| Unigene26240 | 461 | 0 | 0% | TTCATGCCCAACAAGCCAATGGAAAATAGACCCGTCAAAACTCCCACTGA  CAAAGTATTCTTCATGAAATGGATGCCAAGCCAGTGCAGTCACGTCCTTT  CGATGCCCACGGAAAGATTCAAGCTCCTTCATAGCTCTGATGTCATATAG  TTTGATTATCTGATCCTTCGAAGCAGTTAGCACCCAGTTACCATTTTGGT  TCCACTTGACACAAAGTACCGTGTTTTTGTGGCCATGGAAGGAACATAAC  TCTCTCCCACTTTTAGCATCCCAAAGTTTAACAAGATTGTCTTTCCCACC  TGAAACTAGAAGAGATTTTGTAGGGTGCCAGTCAACACTCTTCACATCCC  AACCATGGCCAGTTAATGAACGCTCTTCTTGGCACCGAGCAAAGTCCCAA  ACTTTAACAGTTGTATCATCAGAGCAAGAACAAAACTTCAAGTCTGTCCT  ACAGAAGCTTA |
| Unigene6611 | 362 | 0 | 0% | CATTATATGGGCATGAACCAAATGCACTCAGGAAGTGGTGGTCCTCCTAC  TGTTGGAGGCTTTCCGAATGGCTTGCCAAATATGCAGGGCCAACAGAATC  CAAGTGGGCCTCAAATGTATCAACAAGGTGGTGCATACAACCGTCCACAA  GGAGGACAAATGCCAATGATGCCAGGTTTCAATCCCTACCAGTCCGGGAA  TCAACCTGGTATGCCTCCGCCACCACCTCCAGGTCCACCACCACACGCCC  AGAATCAGTAGACCAGACCATCGTTGTACCATACTTGAAAAGCATCAAAG  AGTTTCTGCGGTCTACAACTGAAACAGAATTTTCTAGTTATAACTGTGTT  TGATCCTTTGAT |
| CL1325.Contig2 | 853 | 0 | 0% | TTACCTCTCTGAACAGACTGACGGGAGTGTTCCAAATACGCTGGTCATTG  CGAATTGTGAGGTTGTCAAACCTAGGGTTGCAGCGGCTGAGCACATATCT  CAGTTTAATGAAGAGGCACGATCTCCTTTTGTCAAGAAGTACAAGACTAT  TATACATCCTGGCGAGGTCAACAGAATCAGGGAACTTCCACAGAATTCTA  AGATAGTGGCCACTCATACTGATAGTCCTGATGTTCTTATTTGGGATGTT  GAAGCTCAACCTAACCGTCATGCAGTTCTTGGAGCGAATCATTCTCGTCC  AGATTTGATTTTGACTGGACATCAAGATAATGCAGAATTTGCTCTGGCAA  TGTGCCCTACAGAACCCTATGTTCTCTCTGGAGGTAAGGACAAGTCAGTG  GTTTTATGGAGTATCCAGGACCATATTGCATCATCATCTGGTGGATCTAT  CATTAAACAAAACTCCGAAGGTAATGATAAAAATGCTGATGGCCCTACTG  TCAGTCCACGAGGTATCTACTATGGGCATGAAGATACAGTTGAAGATGTG  ACATTCTGTCCATCCAGTGCTCAGGAGTTTTGTAGCGTAGGCGATGATTC  TTGCCTCATATTATGGGATGCTCGGGTTGGGTCTAGCCCAGTTGTTAAGG  TTGAGAAAGCACATAATGCAGATCTTCACTGTGTTGATTGGAATCCCCAT  GATGATAATTATATCCTGACCGGGTCAGCTGATAATTCTGTCCGCATGTT  TGATCGACGGAATCTCACTTCTAATGGAGTTGGGTCACCTATCCATAAAT  TTGAGGGTCATAAAGCTGCTGTTCTTTGTGTGCAGTGGTCTCCTGACAAG  TCG |
| Unigene3138 | 672 | 0 | 0% | TGGTCTCCTGACAAGTCGTCTGTATTTGGAAGTTCTGCAGAGGATGGTCT  TTTGAACATCTGGGATTATGATAAGGTTGGTAAAAAAGTGGAACAGGGAG  CTAGATCCCCAAGTGCTCCAGCTGGGTTATTTTTCCAGCATGCTGGGCAC  AGGGACAAAGTTGTTGACTTTCACTGGAACTCCTCTGATCCATGGACTGT  TGTTAGTGTCTCTGATGATTGTGACACTACTGGTGGAGGCGGCACACTAC  AGATATGGCGTATGAGTGATCTGATCTATCGGCCGGAGGATGAGGTTTTA  GCTGAACTTGATAAGTTCAAGTCCCACGTGGTTTCGTGTGCATCGAAGCC  TTGATACATATATGGAAGCTGAAAATGAAGATTTGTGATAGCTTATCATG  TTGATGGAGTCGTTGAGTTGTTAAGATTGATAGAAATTAGTAGGGATGTT  TCTATAGGTGTAGTGTTGAGGAGTTTAGGTTTTCAGAATTGAAGCTGGAC  GATTAAAGATTTACAAGCATTTCCTTCGTTGGATTTTATGAATTCTTCTT  TGTACACTAGAAATTCCTTGCTAAATTGTCATTTATGTTTAAAAGCCTTT  GCGAAATGAAATTGTAAGAACTCCCAATAACTTGATTGAGTAAAACATTA  TGAATTGGAGTAAACATAACCA |
| Unigene18631 | 515 | 0 | 0% | AGCCAAAATCCCTAACGGATTCCCCAATGTCCCTGTTAACACGCTCCCGC  CTAAATCCGCCCAAGCACTCATTCTCTTCCCTTCCCCTGTCTCCATCTTC  TTTGTATAAACCCGTCCACCAGCGCGTTTCCTCCCCTCTAAAACGGTAAC  CCTAAACCCTAACCTGAGCAGCTGGCGAGCCGCCGCCAGCCCGGCTAGCC  CCGCGCCAATGATCACAACTCTCAGCCCGTTGCTACTTGTCGGCTCCTCC  GGAATCTTCTCCTTGATTTCCCCGAAGTTTATGTAACCGTAGGTGTTTAG  ATAGTTATAGGCCGAGTCTAGTAGATGGTGACAATGTAGAGGGACGGCGT  CCACGAACATCTCCTTGTGGACCCAACGCGACACGTTCTCGCGCCATTTG  GAGATTATGTGGTTGCGGATCAGAATGTAATTGACCTGCTCCAGCCCCCC  CACCACGCGACACACCCCAAACTCTATCTCCTCTTCTGTTAATGAGTCCG  CCGGGAACCCTGCCG |
| Unigene15818 | 1379 | 0 | 0% | AGGGGTTGAGGTGATTGCTGGCAATCAAACGTTTCAAGCAGACATGGTCC  TATGCACCGTGCCTCTTGGAGTGCTAAAGAAAAAGACGATCAACTTTGAA  CCAGAGTTACCTCAGAGAAAACTTGCAGCAATTGAGAGATTGGGCTTTGG  GCTTTTGAATAAAGTTGCCATGATTTTCCCATATGTATTTTGGGGAGAAG  ACCTGGACACTTTTGGATGTCTCAATGAACAAAGCAATAAACGTGGGGAA  TTCTTTTTGTTCTACAGTTATCATACTGTTTCTGGAGGTCCTGCTTTGAT  TGCGCTGGTGGCTGGAGAAGCTGCAAAAATGTTTGAATGCACTGATCCAA  CCTTTTTGCTTCATCGTGTTTTAAATGTCCTTAGAGGTATATATAATCCA  AAAGGCATTGGCGTGCCTAATCCAATACAATCAATATGTACTAGGTGGGG  CAGTGACCCCTTTACTTATGGTTCCTACTCTCATGTTAGGGTACAGTCAT  CTGGCAGTGATTATGATATACTTGCAGAAAGTGTGGGAGGTAGGCTGTTC  TTTGCTGGTGAGGCCACAACTAAGCAATATCCAGCCACCATGCATGGAGC  CTATTTGAGTGGATTAAGAGAAGCTTCATATATTCTTCGAGCCACAAGGG  GTCTAAAAAATAATTTGAGGAGGTCTGTCCTGAGGAATGTTGGGTCAAGC  AATGATATACTGACAGATCTATTCCGAAGACCGGATCTAGCATTTGGAAA  GTTTATGGTTGTATTTAATCCTTTAATGGAAGACTCAAAATCAATTGGAC  TTATGAGAGTTACATTTGGAAGTGATGATGATGAAAAAAAGGTACTAGAG  AACAACTATAAACACCCTTCAAACCTACCGTTGCAGCTCTACACACTGTT  ATCTCGTGAGCAGGCACATCAACTGCAACAGTTGACTGGAGGAAATGAAT  GTAAGTTGTCTTATTTGACGCATAATCTTGGATTGAAGCTAATGGGATCT  AATGCTTTGGGCACTTTAGCTAGTTCTTTGATTTCTAATATTGCTAATGC  AAGAAGATCAGGTAGGGGCAGGAACCGCATTTCTGCCATACAACAAATTA  CTTTCTAGACTTGTTATCACTTCTTTCAGGTGCTTCTTTCTTATGGAGCC  TAATAAAAGTACGCATGGGCTCCATAAAAATTCAAACGAGTTCCAAAAGT  TAGTATGCTTTTGTTTCCGGAGGTGCTATTTTTTGTAAATAAATAGATCC  GATTAGGATATTCATTAACTTCAAGAGAATTTTCTTGAAGTTGGACTGGG  GAAAGCTCATTTGTTGTTTGTCTACAGTAGAAATTTTTTGTTATTGACCA  TGACCAACTTGACAACAGTTGGTCCTTAA |
| Unigene18312 | 384 | 0 | 0% | TCAATGGGGAGAAAAGATGATGGTCAACCAAGTAATGTTTTGTGGGTTGG  CTACCCACCTTCTGTTCAGATTGATGAGCAAATGCTTCACAATGCAATGA  TATTATTTGGCGAGATTGAGAGAATTAAGAGTTTTCCATCCAGAAATTAC  TCTTTTGTTGAATTTAGAAGCGTTGATGAAGCCCGGCGTGCCAAAGAGGG  TTTGGAGGGTCGGTTGTTTAATGATCCTAGAATCACAATTATGTATTCTA  ACAGTGAGCTGGCACCAGGAAAAGATTACCCTGGGTCTTATGCTGGAATG  AGAGGGCCAAGACCAGAGATGTTTTTCAGTGATCATCCATTTAGACCGCA  GATGGATGCTCCTGGCCATAATCATTCAATGCTG |
| Unigene2059 | 451 | 0 | 0% | CCTTGGGTAAGAAGCATCTGCATTGAAAGATGACAAATTTGTGTCTTTGA  CCTCACTTGGATAGTGCAATGACCCCTGACTGGAACCAGGATGATCCAAC  CTCAAAACAACACCAGAAATGCTCAATTTGCCAGGTACTTTCAGTACTTT  TTCAGAAAAATCTGATGGGGGTACAAGAAACAAGGTATTACTGTCATCCA  ATTTAGCCACCGCTGCTCGCTGCTTCTCCTCAAGATAATGCATAAATTCA  TTGTAAGACCCCATATCAGCATCACTTCCAGGAACAAAAAAGACAACCCA  AGCACCTGATGCTTGATAGTAATGTTTAGCAAGCATGTCTAGACTAGTCC  TTGCAGTACAATCTAAGAACTCAGGCAACATCATGTCCATAACTTTTCCC  ACAGGAAAGCAGCGAGCACGACAGACAGGAGTTCCTCCCTTTGCTATAGT  T |
| Unigene5074 | 377 | 0 | 0% | AGCAAAAGTTGTATAGATACATACAATAATCCGGAAAATAAACGAAAATG  AGGCCTAAAAGGATAAATAATGAGAAATCGCAGAAATGAGAACAAACCGG  TCGAGCGAACTCGATTTTGATGGGGGTGCCGTGGAGAGGGGTGCCCTGGA  GAGCCTCTTTCGCGGCTTTGGCGTCATCCACTCGCTTGAAGTACACGAAC  GCGAAGCTCCGTGAAGAGTACGTTGCCACGCTGTCCAACGCGCCGTACCT  CACGAACAGCTGCATCAGATCCGAGTCGTTTGTTTCCGGCCTCAGGTTCC  CCACCCACAGGTTGTTCGACGGAGCCGCCCGCTCCTCCGACAAACCACCC  TCGCCTCTGTTACCCCGTGACATCCAG |
| Unigene7507 | 1276 | 0 | 0% | GAACAAGTTTTGCCAGTTGAATATAATAGGTTTTTACATGATGATTCAAA  GGTTCCAGCTGAACAACATTTTCTGCATGCTAGTGAATCTTTTCCAATTC  AGCCAAGTTCCTTGGATCGTGGGTCAAGTAATAGTGCTGCTGTCTCTCAA  GCTGGAGTTGCTTTGACTCCAGAACTGATTGCTACTTTAGCTTCATTCTT  GCCTTCAGCTCCTCAGTCTTCTGCTGCAGAAGGAGCTCCACCAACATTGA  GCTCCTCCTCAATCAGACCGCAATTACCTCAATCTTTTCCTCCAAGTAGT  ACTTCTTCTCATGGGCTCTATGTAGATAATGCATCATCTGAGTCTGCTGG  TCAGTCAGTTGAACGGTTAGGAAATCCATTAAATCCTATGCCCCAATCTC  AAGTCCATTATTACTCATCTTTTGGGAGTACATCGAACCAGTCTGCTCAG  GTGGTACATGGAAGTACTCAATTTCAAGAGTCAAATGTCAGCTTGCAACA  TCAAGGCACGTTGTCTTCTAGGCCATTGACTAATTTCTCAATTCCTCCTC  AAAGTGCACACATTGTTGTTTCCCCTTCCGTCACTCATCAGTATCAGTAT  GATGTTCCCCCCAACAATCAAAGAGTTGGTATGGTGCATGCAACAGATGC  CTCCACTGTATATGCTTCACAAGCTTTTCCTCAAACTGGAAATGCTGCTG  CCATGTCAAATCCTGTTACTTTTTCCCAACCACCAAACATCATGCCCTTT  TCAGCTGAAAAAGTGAATCTGGATCACCCAAATCAGGTACAGCAGCTCCA  GTCTGTGCTCTCTGGAGCTGGTCAGGGCATGTCTGAGGATGAGGTTGATA  AGAATCAGCGATACCAGTCAACACTGCAATTTGCAGCTAATCTCCTCCTC  CAGATACAAAATCAGCAACAGCAGCAGCAGACAAACACTCCTGGAGGGCG  AGGGACTGGAAATCAGCTTTGAGATTTTGCTAATCTAGGTGGTTTGGGCC  AACTATGTGTTTATCATTACAAAATGGCACATTTATTAAGTATAATGGGC  AAAAAAGGTGATCTAGTTCTACATTGCATTGGATTGGATTTGCAAAAAGG  CTTTTAATTGTCAATTCATAGGACTATTTGCTGTCGGCTCTTGGAGATTG  TACATAATGGTTCCCCTCCATTTTTGGTTTTTCCTTTTTATTTTTTCAAC  TTTTGTAAGGTTTATCCTTTTTATTTTTTATCACAATCAGTCAAATGAAA  CTATCTTGGTTTAACTTTTCATGCCA |
| Unigene9309 | 435 | 0 | 0% | ACTTGTTCATCCTTTGGACGGGGTAAACTGTAATCAGCACGATGCGGCGG  AATGGTCTGTCTATCAACAATCTGTGATGAAGGCATTGATTGCTGAGGTA  ATTTGAGTACAACACCATATAAACGCTCTGGACCCATTACTTTCAATACC  TTGGTTAAGAAATCTGATGGAGGCACCAAGAATAAAGTTGTTCCATCATC  AAATTTTGCAACACCTGCACGGTCTTTTGAACCCAAGTAGCGTAGAAATT  CTGTATAAGAAGCAAAGTCATCTTCACTGTCTGGCAAAAAGAAAACAATG  TCAAATCCAATAGAATCAGCATAGTGCTTTGTGAGCATATCCAATCCTGT  CCTAGCTGAGCAATTAACAACTTCAGGCAACTCAGTCCCTATCCCTTTGC  CAATTGGCACACATCGAGCATGACAAACTGGCGTA |
| Unigene12858 | 440 | 0 | 0% | GTTTTTTATTATATATAACCAGTACCCACTACTATTGTTGGCTCCAACTT  ATCTAAAATCCAAGACACTACTCTTTTCATCTAAGCACAAATTCTCCCTC  TTTTTCTTCTCTCAAAGTACAATCTTCTCTTGAGCTTTGATTTCACAATC  TCTCTCCCTCTCTCTCTCATCATGCCTTCAAGACTCTCCGATGCTTTTAG  AGCCCACCCTGTCCATCTCCACCACAAACACCTAGACTTCAACTCACTCC  AAGAACTACCTGACTCCTACGCTTGGACTCAACTCGATCATGATCATGAG  TACTCACCTGCCGGCGGGGACTCATTCGTCTCCGAAACTGTGCCGGTAAT  CGATCTCCACGATCCTAATGCCCTTCAACTCATAGGCCATGCATGCAAAA  CGTGGGGAGCTTTCCAAGTTATAAACCATGGCATCCCAAC |
| Unigene21359 | 238 | 0 | 0% | GGGGGAGGATGACTTGGGCCTACTAGTTTTGGAAGTGGTGAGATCTGGAC  ACTTTCTGGTGGCCCATAGAGATAGGCAATGGATAACCTCTGCTTGCTCC  GGTTCACCACTGCCCGATGGAGCACACTCGGGTATGACCCGTTTGACAAT  ATGTGAAGCAAGTCACCCACGTTGACAACCAGTCCACCCGGGACAGGAGG  GACCGTGACCCACCCAGTTCCTTCTCTTAGAACCTGCA |
| Unigene25332 | 300 | 0 | 0% | CACCCAGTTCCTTCTCTTAGAACCTGCAATCCACTAGTGCTGTTTTGGTA  GAGGATGGTGAGAAGGGTTGAGTCTGTATGTGCAGCTAGACCCATGGCCC  GGTCCGGATCCGGACAAGCCGGGTAGAAATTCAATTGCAATGCTGAACAG  GCATCTTTGTAATCACCTTTTGGTCCAGCCCATTTAATGTCTCCCTTTGT  TATGCCTAATGAGCCCAGCATTAGCCACATCAATCTACCTGCTAGCTTTT  TCATTTCTTTCTCATATTCTTCAATTACACCACAGTATTCGCTATAATCT |
| Unigene25388 | 546 | 0 | 0% | GGTTTATATTGACTTGGTCTCTGAGACGATACCATCTCTGGAAGTGAAGT  GATCCATGCGTCTAGTGGCGGTGCCCCAAAATAACCCATTGACATTCTTG  CCCTCCGTGAGTTCGCCAGCGCTCTATGTCTCACACTTGTAAATCTCCCA  TTCGTCAGAACCTGTAAGGCATCTCCAACAATCACGTAGAGTTCCGTAGG  GTCAGGTGGAACCGGGACCCACAGTCCATCGTGCAAACAAATTTGAAAGC  CACCAACGTCGTTGGATCTCAAGATGGTCAAGATCTGAGGGTCAGAATGC  TCGCCAAATCCGATTCGGCTTTTAGGGTAGTGATCCTGATGAAATTTAGC  TGATGGGTCCCAATCATTGACGTCTTTGACTGCAGGATAGTGATTTAGCC  TAAGTACCGAGTCATTTTGGACGTCTCTGATGAGTCTACTGAAGGCAAAC  TTATCGGTGATCCCCAAGCCCTCAGCCACTAGATCAAGGATTTCACATGT  TAATTCTCTAACCGCTTGTATGAAATCATTCACCGCACAACTGAAT |
| Unigene27444 | 232 | 0 | 0% | GTCCTGATGTGTTGTTCGATCTTAGTACAGAAATTATCTGGGGGTCTGTG  TGCTCTCCAAATCCAATCAAATTCCTACCACTCAGTGCTTGCAACTCTGG  GCATGGTGGGTAGTGGTTCAGCCTGAAACACGAGTCACTCTTGTCATCCT  TCAACAACCTGCTCAGTACATTCCTTGGCTGTATTCTGAGCCCATCTGCC  ATCAATTCCAGTACTTCAAAGGCCATTTTCTT |
| Unigene22418 | 254 | 0 | 0% | GTTGGGTCGCAATGAGGACCTGTGCCTAGGGCCAGCCCTGAATTATTGCA  TGGAGGATAATAATTGCATCTCATTATTGAGTTTCCATCTTCAAAATATT  TCTTATAGTGCAGACCATCTTCAACCCCCAAGCTAATTGCCAATAGCTCA  AAAATTAGATCAGATAGTTTCTTCATTGCTTCACAGTACTTCTGATAAAC  CCACCCAGTTTGTTCAAAATCTTGTCCTAGAACAGAGTTGAAGTATTCAA  CAAC |
| Unigene4959 | 305 | 0 | 0% | TCTCTCTCTCTCTCTCTCTCTTTATAATATCACTTAAAAAGGCCCTCCAA  AAAGATTAGTATACTCTTTTTAATGCAATAATGGCAATTCACTGCGTCAC  AAATATACCATCCAAGTCTCACCCCCTAAAAGATGAGCAACAACAGAAGC  AGCTGGTGTTTGATGCTTCTGTGCTCAGGCACCAAACCCACATACCAGAA  CAATTTATATGGCCTGATGATGAGAAGCCTTGCCCTAATGCACCAGAACT  CCAAGTACCACTCATAGACTTGGGAAGTTTCCTCTCTGGTGACCCGGTTG  CAGCC |
| Unigene213 | 1107 | 0 | 0% | AGAGTTCTTATGATAAGTTGTTTGAACATATTATCAGAACTGCAATTTGT  CACCCGAGTCATCATACATAATACTCTTGCTCATCGTTTTCACCAAACTA  AACTTCATTTTCTTTGTAATGTTGCATTCCCCAAGAGCTCAAATAACTAA  CATTCATACACACATGACTTCCCTATATGCTGATCTAACCACTATTTCAT  GGATAAATCCAAGGAACAAATTGTTCTAAAGAAAGAAAAGAATTCCTTAA  AATTTACAAGCCACAACTAATTTCTATATTTGAGGGGAAAAAATTGCAAT  ATGTATACCTACTCTAAGCAGCACAATGCCTCAGCTCTTCCAATCCACAA  TACTAGCACAGAACAAAAATCAAACAGCACAGCTCTGCCAGCATTCTTTC  CCCAAAAAATCTGGTTCTATGCAAGATAGCCCTAGGTTGTTGCATCATGA  GACTACCACCTCTGTTCCACTATAAGCCACCGTAAATATACACCAGTCTG  AGCTACATCTTTGTGAAAACAATCCCCAATAATGCCTCCGATAATTGCCA  GTCAATAAAAACATTCATTTTCAACCCAGCTAGCCTGAATTAGTAGTCTG  ATTCAACTGAACTACATTTTCTTCACAATTAGAGGGGTTTGATATGGATG  TTACAGCCTGACTATATCCATTGGACTTGACAGTTCCCAAAGGGCTTTCT  TTTGAGAATGTGACACTCATTGGCTCAGAGGTCTTGATGGCAGATTCTTC  AAAGACCACCTGCTGTTGTAGCATCTCCATCTGCCTTAGAGATGGCACAT  CACCGTTACAGTACCTGTGCCACATGTTCCGGTATGTTGACTCCAAACCT  AGTGTATAGTTTTGCCCATCACAGACAGGGGACTTAGACATAAGGCCCCG  AAGACTCATCCTTAAGTTGGAGAGAGCAGTAACGTCAGAGGCCAACTGCA  ATGCCAACTGGACATACTCATCTTCATTTTTTGCAATCAGATGTTGCAAG  CCAACTTTGTCAAGAAGACTGACACCAACATTATGAGCATGCACTGAACC  AGCCATGGTGACACATGGAACTCCCATGTACAACGATTCACAGGTCGTTG  TAGTTCC |
| Unigene5529 | 559 | 0 | 0% | GTTTGGTTTTATTGATAAAGCCATCTGATAGCAATCTACAGCTTTATCGA  GGTTGTCACGGTCTTTGTATATCACTCCTAAATTGTTGCATGCTTCAGCA  CAATGGGGATTGAAATGAAAAGCAAGTTCATAAAAAACAATTGCCATGTC  AAACTTCAGCATCTCACCATAAGCAACCCCAAGATTATACATGGCATCAG  CATAGTGCCAATTATAATACAAAGCCTTCTTGTAATACGCCACACCTTGA  TTAATGTCTCCTTCCAATTTAACCTTTGTTCCCAAATCAGTTAAGGCTAT  TGCCATATTGTTCTTTGCAATCTCAAAATTTGGAGATACAGCTAAACACC  TCTCATAACAGGCAATAGCTGACTCTAAGTCACCACGATTTTTATAGATG  ACACCCATATTGCAGTATGCTTCAGCATACATAGGCCTCTCCAAAGCAGC  CTTGTCATAGCAATTAAGGGCCGTGTCATATTGCATCATTTCAGAATAGA  CAACGCCAAGGTTATAATATGCAGGAGCATAATGAGGATCTATTTTTAGG  GCTTCATAG |
| Unigene15359 | 508 | 0 | 0% | TTTGAACAACCTTGGCGTTGTTTACACTGTTCAGGGTAAAATGGATGCTG  CTGCAAGCATGATTGAGAAAGCTATTATTGCTAATCCAACATATGCCGAA  GCATATAATAACTTAGGGGTTCTCTACAGAGATGCTGGTAACATATCGCT  GGCTATTGATGCTTATGAGCAATGCCTTAAGATAGATCCCGATTCTCGCA  ATGCAGGCCAGAATCGATTGCTTGCTATGAATTACATAAACGAGGGACAT  GACGATAAGCTTTTTGAGGCACACAGGGATTGGGGTAGGCGCTTTATGAG  GTTATATCCACAGTACACATCATGGGACAACCCAAAGGATTCAGACCGAC  CACTTGTGATTGGATATGTATCTCCAGATTATTTTACTCATTCTGTATCA  TATTTCATTGAGGCCCCCCTTGTCTATCATGACTATGGAAATTACAAGGT  GGTTGTTTATTCAGCAGTAGTGAAGGCAGATGCAAAAACTATTAAGTTTA  GGGAGAAA |
| Unigene15577 | 1333 | 0 | 0% | AAAAAAAAAAAAAAAAAAACCTTTAAGATGGATTGATCAGAAAGGCATGA  TATGAGTGGTCTTAGTATCATAACTGGAAAGGCAAGACTTGTAATTGACC  CGAAAAATCTTCAACAAATCGGCGCTCATTTGCCTGACGGAAGGAGAGCA  ATTGCTCTGCATCAGCAACAGTATCTTGGTCAGGCCATTCCCACTCGTCA  CCGCCTCCTGAGCCCTCTGGTCACTGAACAAATAACACGCGCTCCACAGT  ATCGTCACGGCGTGCTCCGTCGCTGCGCTCGACACCTTCAACATTCTATT  AACAATTGCTTGAGCCAGAGTCGAGTCACATGAAACCTCACTCCTCCCTT  GCTTCGTCGAACACATCATCTCCAACAGCTTCAACGCCTTTTCGCAAACG  GAAGCCGTACAATTAGGGTCGGTTATGAGCTTTTTCAGCTCCGTGATTAT  TTTCATGTTTATCAACCTTAATTTGACACGTTTCGGCATTGATATCGTGA  TTAGACAAGATAAGCCGGCGTCGATGACGTTCACCTCCGAATCGGAGCCT  ACGCATTTCAAAATTTCGGTTAATAATCCGTCGTTTTCGGCGATTTTTAG  CTTGGATTCGGCGTCAACGGCGATAAACTCGAGGATTTTGAGAGAATCGA  TTCTTGAATCTTGTTGTTGTTGTAAAACAAGCAGAAGCGAGCGCAAGCAA  TCTTGATTATCGCTGCTGTTCAATATCAAGTTCTTCAACTGTTGTTGATC  TTCGATTTTGGTCAAAATCAAGTCCAAAACCCTAACGACTCGCTCTAATA  CGTTGACGTCTTTAACATTGCCAAGAATTTCAACAAGCATCGACAAGAAC  CCGTCCAGTTTGGCGAGGAAAGCCTTGTTCTCGTCGGATTCCTTGGCGAA  CGAGACAATCTTAGACAAATCCTCGTTCTTGAAGTCGGTGAGTTGGACTA  TGTCCTTCACTTGCTCCTGAGAAACCACGGAGAAAGCCGTCGTCGTGGAA  TCCTCCTGCCGTCGGACGGAGTCCAACCAGATCTGGATTAGTCGTTGCAA  GGTCCGGTTCGGCACGAATTCTTTACTCTGAAGGACCTGCATCGTCGCCG  GACACGTGTTGTTGCCGCTCTCCAACCACCGTTGGATGCTGGCCCGGTCG  TACGTTACGCCGGTGCAGAGACTCACAGGCGATTTCATCACGTCCAGTGA  TATCGGACACCTGAAGAAACTCGGAACCGTAATGTACAGATCATCCCTCA  CCATTTTTTTTAGTTTTTTTCTCTTCAATGTTTGTGAATGTTTGGCTTCA  CAGAAAATTTTATTTAAAATAAAACCCAACAAG |
| CL141.Contig1 | 1254 | 0 | 0% | GCCGGCTCGATGCGAAAGGTCTAATTAATTGCATCATTAAATCGTATTAA  TTTAATTTAATTATTAAAATTAAATTTATTTGATTTCCGTAGGTCGCGAC  CTACTTCGCTCAAGGCCTGGCGCGGAGAATCTACAAAATCTACCCTCAGG  ATGGTCCGTTGGAGTCGTCCTACGACGATATCCTCCAAATGCACTTCTAT  GAGTCCTGCCCTTACCTCAAATTTGCACACTTCACAGCCAACCAAGCTAT  CCTTGAAGCCTTCGCGACGGCCACGCGCGTCCACGTTGTCGACTTCACGC  TCAAACAAGGCATGCAGTGGCCGGCTCTCATGCAGGCTCTCGCGCTCCGG  CCCGGTGGCCCGCCCGCTTTTCGGCTGACCGGCATCGGCCCGCCTCAGCC  CGATAACACTGACGCGCTGCAGCAGGTGGGCTTGAGGCTGGCCCACTTGG  CCGACGCAATCGGGGTCGAGTTCGAGTTTCGTGGGTTTGTTGCGAGCAGC  TTGGCGGATCTTGAACCCGAAATGCTGGATATCCGACCCGACATCGAGAC  AGTTGCGGTCAACTCCGTTTTCGAGCTCCACCGGCTGCTGGCCCGACCCG  GTGGGATTGAAAAGGTTGTGTCTTCGATCAAAGCGATGAAGCCGAAAATC  ATTACGGTTGTGGAACAAGAAGCGAACCACAACGGTCCGGTTTTTTTGGA  CCGGTTCACGGAGGCTTTGCATTACTATTCGAGTTTGTTCGACTCACTAG  AAGGGTCCGCGTTGACTCCACCGAGTCAGGACGTGGCCATGTCCGAGGTG  TACCTGGGGAGACAGATATGCAATGTGGTGGCTTGTGAGGGAACGGATAG  GACTGAGAGACACGAGACGTTGACTCAGTGGAGGACTCGGTTGGAATCGG  CCGGATTCGACCCGGTCCACCTCGGCTCGAATGCATTCAGGCAGGCTAGT  ATGTTGCTGGCTTTGTTTGCTGGCGGAGATGGGTATAGGGTGGAGGAGAA  TAACGGTTGTTTGATGCTTGGGTGGCATACGCGGCCGCTTATAGCCACCT  CTGCTTGGAAACTCGCCGCCACTGAGTTGTGATGGACTCAGTGGGGTATT  TGACTGAGTTGAGGATTCGAGTTTGTGAGGGAGAGAACTAAACTAAATCA  GCCAATCCAAGTGTGTAATTGTAGTTTTTTTCTTTAATTTAATTTTAATT  TTAATTTTTTTTTTGGGTTTAATGTGTATGAATGACCATGTTTAATGTAA  TTTG |
| Unigene15619 | 1601 | 0 | 0% | TGTTTTTCTCTGTTTTTCTTCAAAATATGAGGCTGCTTCTTTGAAGATCA  CATTCTCTGCTGGGTTGGTTTATTTCTTCTTTTAAAGGAGTTTGTTTGGT  TCCTTCAGTTTAGGACTTGGTTTTTTCAGCCATGGCTGGTAGTAATGAAG  TCAATGATTCAAAGAGAGTTGTTCCACTGAACACATGGGTGCTCATTTCT  AATTTCAAGCTCGCTTACAATCTCCTTCGACGTCCTGATGGAACGTTCAA  TAGGGACTTGGCAGAGTTCCTTGACAGGAAAGTGCCAGCCAACACCATTC  CTGTTGACGGGGTGTTCTCTTTTGATCACGTTGATCGAGCCACGGGTCTG  CTCAACAGGGTCTACCAGCCTGCTCCCCAAAATGAGGCTCATTGGGCCTT  GGCAGAGCTTGAGAGGCCCTTGAGTACCAATGAAATTGTCCCAGTCATAG  TTTTCTTCCATGGTGGAAGTTTCACACATTCGTCGGCCAATAGTGCAATC  TATGACACCTTCTGTCGCCGCGTTGTTAGCATTTGCAAGGCTGTTGTGGT  GTCCGTAAATTACCGTCGATCACCTGAATACAGGTACCCCTGTGCATATG  ATGACGGTTGGGCTGCTCTTAAGTGGGTCAAGTCTAGAACTTGGCTTCAA  AGTGGTAAGGATTCGAAGGTTTATGTTTACTTAGCCGGGGATAGTTCTGG  CGGTAACATAGCTCACCATGTCGCGGTGAGGGCAGCCGAGGCAGAGATCG  AAGTACTGGGAAATATACTTCTCCATCCAATGTTTGGTGGCGAAAAGAGA  ACTGAATCAGAGAGCAGACTGGACGGGAAATACTTTGTCACGATACAGGA  TCGCAACTGGTATTGGCGAGCTTTCCTCCCCGAGGGGGAAGATAGAGATC  ATCCAGCCTGTAATCCATTTGGGCCTAGGGGCAAAAACATTGGAGGACTC  AAGTTTCCGAAAAGTCTTGTTGTTGTGGCTGGTTTGGATCTCGTTCAAGA  CTGGCAATTGGCATATGTTGATGGTCTCAGAAACACGGGCCACGAAGTGA  AACTCCTTTTCCTGAAGGAGGCCACGATCGGATTCTACTTCTTGCCAAAT  AATGATCATTTCTACTGCCTCATGGAGGAAATTAAGAACTTCGTGAGTTC  TGACTGTTAATAATCTACTTAACCTTAACTACAGGCAGCTATACATAACA  AAAAGCTTGACATTTCACTCGGGTATTTTTTTTTTTCTCCTTTGGCAGTG  ATGGTGTGTAGCTATACTTATAAGATTGTGTAGTATTACTTCTTACTGTG  TTACCATTTTTGGTGGAAAAAATTAGAGTTCTGCATTCTTTGTTACTGGT  TCTAGCTCCGGAGTAAGGATCTTATAGGCCTATGTGTCTTAGATGGATGA  AAGGCAAATCTATCTCAGAGCTGCCAGTTTCTGTGGTTTGAAGGATCAGA  ACTTGTTGATCGGATTTGGCTGAACCACCAGATTTAGAGCCTACCCCTGC  TAACTTGGGGTATTACTGTGAGTTTGGTGGTTAATCAATTGGTTGTAGTA  AAGTGGGGTAATTTGTAAGCACTAATTTCAAAATGTGAACTGAAATATAT  A |
| Unigene1628 | 937 | 0 | 0% | CGCCCAGATTACATACATAAGTTGAAAAAGCAAAATCCAAGCCAAATTAA  GCAAAAAAACAATGAAAAACGCCAATTAATTAAAAAGATATACATATAAT  AGTTAAATTTTTGCATCAGCTTCGGTTACTGGAATTCATGGCCTCATAAT  AACGAATCGAAAGCAGAGACAAGGAAAGATGGACCTCATCTCTCCCCCAT  CGGGCCGCTGGCTTTGGTCCGATCCTCGGGAACGGAGACCAATTCGAGCC  CGAGCCCGAGCCCGAACCCGACCCCGACCCAGAAGGACCCCTGGAGAGCT  GCCAGAGATAGAGCGAGTGGAGGCGACGGAAGCCACCGAGAGCGAGGACG  ACGGATCTAAGCTGCTGGTAGCCGCAACCAAAATTGGCCCAGTGGCGCGT  GCAGATCAGCTCCCAGAGACGCTCGTCCTGGGCCGTCTTGTGCCACTGCT  TGCTGACGCAGGAGGCCGTGGCCAGTGTCTTGGCGTCGACGTGCTTCAAC  ACCTCGAAGAGGAGGTTCTCATCGAGGTTGGCAAACCCAGTCTCCTTCAC  TTCCTCCTGTTCTTCTTCAACTTCGACTTGTTTTCCATTTCCATGTTTGT  ACCCATTATCATCATCCACCTGTCTGATTTTCTTCATCTTTTGGTCGCCT  CCCGGAAGATCGACGACCGTACGCTTCATGGCTGGCAGTAGCGTTCGGAG  GTGTAATGTGAGGGAGAGGAAGTGATTGCTGGTGGGTTTGGGTTGGGTTT  GGAGTTGTGGGTGTGGGTGTGGTGTGTGTTATGGGTTAGAGAGCTGTTTT  GGTGGGTATCGAAGACGACAATGCAAGAAATCAATGCGCTTCGATACTTT  TTTTTTCTTTTTATCTTTTTTCTTTAATTGACCATCTTGTCCTTCTTTAA  TCTTCCTCTCTCTCGTTTGTTTTGTTTTTAATACTTG |
| Unigene12569 | 1076 | 0 | 0% | GCAATTTTGTACTATTTTATTTAAAAATAAGATAACTGACAGCCGGTTGA  TTAGCTAGTGCTCTCTCTCTCTCTCTCACAGCATATTAACCTTGATTCAA  ATTCATTGAGTGCCATTTCATGAATAGAAACGGACAAGGCCAACAATTAA  AATACATATATACGATACTCTACTAATAGCAAACCATCAAATTACAGCAC  AAATGCATGGCCACTGAATTATTGGGCATTGTTGCTTGTAGATATTTATA  ATGTTTCATCAGGAATAAGCAAGAGGCACCAACTGGCCGGCTTGGAGCAT  AATGCATGGAATATAGCTAATAATGATTTGACAGAAGAGAGAGAGCGCAC  AAGAGAATCTTATAGTTAATAAGACCTTTAACTGATGAAATGAAGCTCTA  ATATGCTGTAACAGATATATCCTTCTCAGTTTGAGCCTTCTCCATGGTAT  AAATCAGCGGAACTTTTACGGCGACGCTCGTTGTGTCCAGCCAAACGCCT  ACGACAACTTCTCTTGGCTTCATCAAACTCTGATACTTCATGGAACCTGC  TACATTGCTGGCAGAAACGTTGTTGGAGTCCGGCAAGACGAACAGTAGCA  GCCTTAGCATGGAACTCACAGACCTTGTGGCGCCTATGGTATCGCTTGGC  GTCGGACATATCAACCGTACAGTTATCCACTTGACAAGAAGCAGCAGCAC  TGCTTGATCCTTTCTTGCCTTTCCTCTTCTTGTCATCCTCTCCAAACCCT  AACCCACTATTATCTTCATCATCATCTTCTTCTTCATCCTCCACCACCAC  CTCCTTCAAGCTCCTCTTCCCTTCAGCTTTGCTTGTGTCCATCTTGTTTG  AAGTAAGTAAGAGCTAGAAGGAAAATTACAACTATATATCCAAGGAGATG  GATCGAAGAGGAGGAGGAGGAGGAGAATTATTATAAACCTGGTTGATAAG  GTTAAAAAAGAGAGAAGAATAATTGAAGGACCACAATTTCCCATTTGATT  TTCACCATCACACCAGTAAAGGACATAGGAGGCTAAGATTTATGATTGCT  CCATATACACGGACATATTGATTTTT |
| Unigene4477 | 1690 | 0 | 0% | CTTGTTTACTTCTAAATATAATATATAACCCATCTCTCTCCATTACTATC  TCTTTCTCTCTCTCCCTCTCTCACTCACACACGCTCTCATGATGATGACC  TACAATACCTCCATGAATGGATACTAGTGACAGCAAATGGAACTGGGTTC  AGGCTCTTTGACTGAGTCTGGTGGTGGCTCTTCTCCACCAAACTCCTCAA  CCGAGTCACTTAATGGTTTGAAGTTTGGGAAAAAAATCTACTTTGAGGAT  GTGGGTAGTTCAGTTAAAGCATCTGGAGCGGGCTCATCATCAACAACAAC  AACGGGGTCATCAGGTTCAGGTTCAGGGCGGAAGGTGAGGAGTGGTGGTA  GTGGAGTGATGCAAAGTGGGCAGCCACCAAGGTGCCAGGTGGAGGGAT  GCCAGCTGGATCTAAGTGATGCAAAGGCTTACTATTCTAGGCATAAAGTCTG  TTCCATGCACTCTAAGTCTCCCATGGTCATTGTTGCTGGCCTTCAACAGA  GATTTTGCCAGCAGTGTAGCAGATTTCATCAGCTTCCTGAATTTGATCAA  GGAAAACGAAGTTGCCGCAGACGCCTGGCTGGCCATAACGAGCGTCGAAG  AAAGCCGCCACCTGGATCACTGTTGGCTGCTCGCTATGGCAGGCTCTCTT  CTTCTGCCTTTGAAAACAGCAGTAGAGCTGGAGGATTTCTAGTTGACTTC  AGTGCATACCCGAGAGGAGTTGCTAACAGGGACCAATGGCCAGGGACCAG  ATTGTCTGAGCGGGTACCTGGGAATCAAACCACTGTCACAGCAAGGCCTC  TTCCACATCCGCCATGGCAGAATTACTCTGAGAATCCTCCATCTGATGTT  TTCGGACAAGGTTCAGCTGGAGGGACTGGCTTTTCCAGTCCTGGAATTCC  TTCAGGAGAATGCTTCACAGGAGTTTCTGATTCAAGTTGTGCTCTCTCTC  TTCTGTCAAATCAAACATGGGGCTCCAGAAACCGAGCATCAGGTCTTGGC  GTGACTGACTATCAGCATGCTCAAGGGGCACCCATGGCACAACCAGCATC  ACCTCGTGGTGTTGCTGTTGACCAATATCCAAACGTGTCGTGGGGTTTCA  AAGGCAACGAAGCTGCCAGTAGCAGCTCACACCAGATGCACCCACATATA  GGTTTGGGTTCCATTTCACAGCCTGTTAACAGTCAGTTTTCAGGTGGGCT  CGAATTATCCCCACAGAGGAGGCATTATGCGGATGTTGAACAATCCGGGG  ATTATGAGGACTCCGCTCCACAGATGAACTGGTCACTTTAACTACTCCTT  TTGTGTTCGGTTGTTATCGAACTTAACCAGGCCTGCCTGGTTGTTTTCTT  TTGAATTGTAATGATTTCCCTACCATCAGGCTTATGACCTTTTTGTTTTT  TGTTGGGCTACTACCAAACTGACTCCTGGTTTGTATCTTCCTAGGACTGT  AAAACGCTTGTAAGCCAACTTCAAGGCTTGTTCTATTCAGGCTTCAGTCA  GCTGGTGTCTGAGTATCAATTATCAACTGTTTGTCTAATATCATCATGGC  AGGAAAATGCTTTAAGAAAATATTGTGATGACAGTATCCAGAGCTGCCTA  TGACTAGCGCGGCTTATTAATTATTCAAGGATTCCCTCTCAATGACTGGT  GATGGAAGACAACAGAGTGTTGAGTTTACCTCATTTCCAG |
| CL1113.Contig1 | 903 | 0 | 0% | CGCAGAACAGGGAGATGGGAATCACACATTTGGGATTGTGGGAAGCAAGT  CTACTTGGGGGGATTTGATACTGCTCATGCTGCAGCCAGGGCCTATGATC  GAGCTGCAATCAAGTTCCGAGGAGTTGATGCTGATATCAATTTTGATGTT  ACTGATTATGATGAAGATATTAAGCATATGAGTAGTTTTACCAAGGAAGA  ATTCGTCCATATTTTGCGTCGCCAGAGCACTGGCTTCTCCAGAGGAAGCT  CGAAGTACAGGGGAGTGACGCTGCACAAATGTGGGCGGTGGGAAGCTC  GCATGGGGCAGTTTCTTGGCAAGAAGTACACATATCTGGGATTATTTGATAC  GGAATTGGAAGCTGCAAGGTCTAATGGATCACAAGTTTGATTTTTCTTTT  TTGCAGAATCCTAAATTACAACTTCATTTAACCTGTTGTGTTGCTATTCC  GCAATTCAGGGCTTATGACAAGGCTGCCATAAAATGTTATGGAAGGGAAG  CAGTTACCAACTTTGAGTCTATCACATATGAGGAGGATATAATCTCTGAA  GCCAATAAAGCTGGTGCAATTTGTTGTTTTGTAATTATATTTATTGTTCT  TATTAGTATTGAATTTTGTGAGCTAATAAATCTTTTGGCGTAGACGGCAA  CAACAATCTAGATCTGAACTTGGGGATTGCCCTCCCACATACACCCAATG  GTCACAACAATACGAGCAATTTGAGAGGATATCAATCCGAAAATGGCTGG  AATGACATGCTGCTTAACATGGGAACAATGCCAATTGAGAATTCTGCATC  AGTGGCAGGGAGGGTCCTATCCGCACAAGGCCCTGCAATGGCTTCTGATC  ACCCTTCCATCTGGAGCGGTGTATATTCAAATTCTTTTCAAATTTACAGG  GTA |
| CL1113.Contig2 | 823 | 0 | 0% | CGCAGAACAGGGAGATGGGAATCACACATTTGGGATTGTGGGAAGCAAGT  CTACTTGGGGGGATTTGATACTGCTCATGCTGCAGCCAGGGCCTATGATC  GAGCTGCAATCAAGTTCCGAGGAGTTGATGCTGATATCAATTTTGATGTT  ACTGATTATGATGAAGATATTAAGCATATGAGTAGTTTTACCAAGGAAGA  ATTCGTCCATATTTTGCGTCGCCAGAGCACTGGCTTCTCCAGAGGAAGCT  CGAAGTACAGGGGAGTGACGCTGCACAAATGTGGGCGGTGGGAAGCTC  GCATGGGGCAGTTTCTTGGCAAGAAGTACACATATCTGGGATTATTTGATAC  GGAATTGGAAGCTGCAAGGTCTAATGGATCACAAGTTTGATTTTTCTTTT  TTGCAGAATCCTAAATTACAACTTCATTTAACCTGTTGTGTTGCTATTCC  GCAATTCAGGGCTTATGACAAGGCTGCCATAAAATGTTATGGAAGGGAAG  CAGTTACCAACTTTGAGTCTATCACATATGAGGAGGATATAATCTCTGAA  GCCAATAAAGCTGACGGCAACAACAATCTAGATCTGAACTTGGGGATTGC  CCTCCCACATACACCCAATGGTCACAACAATACGAGCAATTTGAGAGGAT  ATCAATCCGAAAATGGCTGGAATGACATGCTGCTTAACATGGGAACAATG  CCAATTGAGAATTCTGCATCAGTGGCAGGGAGGGTCCTATCCGCACAAGG  CCCTGCAATGGCTTCTGATCACCCTTCCATCTGGAGCGGTGTATATTCAA  ATTCTTTTCAAATTTACAGGGTA |
| CL1113.Contig3 | 814 | 0 | 0% | CGCAGAACAGGGAGATGGGAATCACACATTTGGGATTGTGGGAAGCAAGT  CTACTTGGGGGGATTTGATACTGCTCATGCTGCAGCCAGGGCCTATGATC  GAGCTGCAATCAAGTTCCGAGGAGTTGATGCTGATATCAATTTTGATGTT  ACTGATTATGATGAAGATATTAAGCATATGAGTAGTTTTACCAAGGAAGA  ATTCGTCCATATTTTGCGTCGCCAGAGCACTGGCTTCTCCAGAGGAAGCT  CGAAGTACAGGGGAGTGACGCTGCACAAATGTGGGCGGTGGGAAGCTC  GCATGGGGCAGTTTCTTGGCAAGAAGTACACATATCTGGGATTATTTGATAC  GGAATTGGAAGCTGCAAGGTCTAATGGATCACAAGTTTGATTTTTCTTTT  TTGCAGAATCCTAAATTACAACTTCATTTAACCTGTTGTGTTGCTATTCC  GCAATTCAGGGCTTATGACAAGGCTGCCATAAAATGTTATGGAAGGGAAG  CAGTTACCAACTTTGAGTCTATCACATATGAGGAGGATATAATCTCTGAA  GCCAATAAAGCTGGTGCAATTTGTTGTTTTGTAATTATATTTATTGTTCT  TATTAGTATTGAATTTTGTGAGCTAATAAATCTTTTGGCGTAGACGGCAA  CAACAATCTAGATCTGAACTTGGGGATTGCCCTCCCACATACACCCAATG  GTCACAACAATACGAGCAATTTGAGAGGATATCAATCCGAAAATGGCTGG  AATGACATGCTGCTTAACATGGGAACAATGGTAAATGCAGAATCCTTCTC  AATTAAAATTTCAT |
| CL1113.Contig4 | 734 | 0 | 0% | CGCAGAACAGGGAGATGGGAATCACACATTTGGGATTGTGGGAAGCAAGT  CTACTTGGGGGGATTTGATACTGCTCATGCTGCAGCCAGGGCCTATGATC  GAGCTGCAATCAAGTTCCGAGGAGTTGATGCTGATATCAATTTTGATGTT  ACTGATTATGATGAAGATATTAAGCATATGAGTAGTTTTACCAAGGAAGA  ATTCGTCCATATTTTGCGTCGCCAGAGCACTGGCTTCTCCAGAGGAAGCT  CGAAGTACAGGGGAGTGACGCTGCACAAATGTGGGCGGTGGGAAGCTCG  CATGGGGCAGTTTCTTGGCAAGAAGTACACATATCTGGGATTATTTGATAC  GGAATTGGAAGCTGCAAGGTCTAATGGATCACAAGTTTGATTTTTCTTTT  TTGCAGAATCCTAAATTACAACTTCATTTAACCTGTTGTGTTGCTATTCC  GCAATTCAGGGCTTATGACAAGGCTGCCATAAAATGTTATGGAAGGGAAG  CAGTTACCAACTTTGAGTCTATCACATATGAGGAGGATATAATCTCTGAA  GCCAATAAAGCTGACGGCAACAACAATCTAGATCTGAACTTGGGGATTGC  CCTCCCACATACACCCAATGGTCACAACAATACGAGCAATTTGAGAGGAT  ATCAATCCGAAAATGGCTGGAATGACATGCTGCTTAACATGGGAACAATG  GTAAATGCAGAATCCTTCTCAATTAAAATTTCAT |
| CL1113.Contig5 | 723 | 0 | 0% | CGCAGAACAGGGAGATGGGAATCACACATTTGGGATTGTGGGAAGCAAGT  CTACTTGGGGGGATTTGATACTGCTCATGCTGCAGCCAGGGCCTATGATC  GAGCTGCAATCAAGTTCCGAGGAGTTGATGCTGATATCAATTTTGATGTT  ACTGATTATGATGAAGATATTAAGCATATGAGTAGTTTTACCAAGGAAGA  ATTCGTCCATATTTTGCGTCGCCAGAGCACTGGCTTCTCCAGAGGAAGCT  CGAAGTACAGGGGAGTGACGCTGCACAAATGTGGGCGGTGGGAAGCTCG  CATGGGGCAGTTTCTTGGCAAGAAGTACACATATCTGGGATTATTTGATAC  GGAATTGGAAGCTGCAAGGGCTTATGACAAGGCTGCCATAAAATGTTATG  GAAGGGAAGCAGTTACCAACTTTGAGTCTATCACATATGAGGAGGATATA  ATCTCTGAAGCCAATAAAGCTGGTGCAATTTGTTGTTTTGTAATTATATT  TATTGTTCTTATTAGTATTGAATTTTGTGAGCTAATAAATCTTTTGGCGT  AGACGGCAACAACAATCTAGATCTGAACTTGGGGATTGCCCTCCCACATA  CACCCAATGGTCACAACAATACGAGCAATTTGAGAGGATATCAATCCGAA  AATGGCTGGAATGACATGCTGCTTAACATGGGAACAATGGTAAATGCAGA  ATCCTTCTCAATTAAAATTTCAT |
| CL1113.Contig6 | 643 | 0 | 0% | CGCAGAACAGGGAGATGGGAATCACACATTTGGGATTGTGGGAAGCAAGT  CTACTTGGGGGGATTTGATACTGCTCATGCTGCAGCCAGGGCCTATGATC  GAGCTGCAATCAAGTTCCGAGGAGTTGATGCTGATATCAATTTTGATGTT  ACTGATTATGATGAAGATATTAAGCATATGAGTAGTTTTACCAAGGAAGA  ATTCGTCCATATTTTGCGTCGCCAGAGCACTGGCTTCTCCAGAGGAAGCT  CGAAGTACAGGGGAGTGACGCTGCACAAATGTGGGCGGTGGGAAGCTCG  CATGGGGCAGTTTCTTGGCAAGAAGTACACATATCTGGGATTATTTGATAC  GGAATTGGAAGCTGCAAGGGCTTATGACAAGGCTGCCATAAAATGTTATG  GAAGGGAAGCAGTTACCAACTTTGAGTCTATCACATATGAGGAGGATATA  ATCTCTGAAGCCAATAAAGCTGACGGCAACAACAATCTAGATCTGAACTT  GGGGATTGCCCTCCCACATACACCCAATGGTCACAACAATACGAGCAATT  TGAGAGGATATCAATCCGAAAATGGCTGGAATGACATGCTGCTTAACATG  GGAACAATGGTAAATGCAGAATCCTTCTCAATTAAAATTTCAT |
| CL1113.Contig7 | 732 | 0 | 0% | CGCAGAACAGGGAGATGGGAATCACACATTTGGGATTGTGGGAAGCAAGT  CTACTTGGGGGGATTTGATACTGCTCATGCTGCAGCCAGGGCCTATGATC  GAGCTGCAATCAAGTTCCGAGGAGTTGATGCTGATATCAATTTTGATGTT  ACTGATTATGATGAAGATATTAAGCATATGAGTAGTTTTACCAAGGAAGA  ATTCGTCCATATTTTGCGTCGCCAGAGCACTGGCTTCTCCAGAGGAAGCT  CGAAGTACAGGGGAGTGACGCTGCACAAATGTGGGCGGTGGGAAGCTCG  CATGGGGCAGTTTCTTGGCAAGAAGTACACATATCTGGGATTATTTGATAC  GGAATTGGAAGCTGCAAGGGCTTATGACAAGGCTGCCATAAAATGTTATG  GAAGGGAAGCAGTTACCAACTTTGAGTCTATCACATATGAGGAGGATATA  ATCTCTGAAGCCAATAAAGCTGACGGCAACAACAATCTAGATCTGAACTT  GGGGATTGCCCTCCCACATACACCCAATGGTCACAACAATACGAGCAATT  TGAGAGGATATCAATCCGAAAATGGCTGGAATGACATGCTGCTTAACATG  GGAACAATGCCAATTGAGAATTCTGCATCAGTGGCAGGGAGGGTCCTATC  CGCACAAGGCCCTGCAATGGCTTCTGATCACCCTTCCATCTGGAGCGGTG  TATATTCAAATTCTTTTCAAATTTACAGGGTA |
| CL1113.Contig8 | 812 | 0 | 0% | CGCAGAACAGGGAGATGGGAATCACACATTTGGGATTGTGGGAAGCAAGT  CTACTTGGGGGGATTTGATACTGCTCATGCTGCAGCCAGGGCCTATGATC  GAGCTGCAATCAAGTTCCGAGGAGTTGATGCTGATATCAATTTTGATGTT  ACTGATTATGATGAAGATATTAAGCATATGAGTAGTTTTACCAAGGAAGA  ATTCGTCCATATTTTGCGTCGCCAGAGCACTGGCTTCTCCAGAGGAAGCT  CGAAGTACAGGGGAGTGACGCTGCACAAATGTGGGCGGTGGGAAGCTCG  CATGGGGCAGTTTCTTGGCAAGAAGTACACATATCTGGGATTATTTGATAC  GGAATTGGAAGCTGCAAGGGCTTATGACAAGGCTGCCATAAAATGTTATG  GAAGGGAAGCAGTTACCAACTTTGAGTCTATCACATATGAGGAGGATATA  ATCTCTGAAGCCAATAAAGCTGGTGCAATTTGTTGTTTTGTAATTATATT  TATTGTTCTTATTAGTATTGAATTTTGTGAGCTAATAAATCTTTTGGCGT  AGACGGCAACAACAATCTAGATCTGAACTTGGGGATTGCCCTCCCACATA  CACCCAATGGTCACAACAATACGAGCAATTTGAGAGGATATCAATCCGAA  AATGGCTGGAATGACATGCTGCTTAACATGGGAACAATGCCAATTGAGAA  TTCTGCATCAGTGGCAGGGAGGGTCCTATCCGCACAAGGCCCTGCAATGG  CTTCTGATCACCCTTCCATCTGGAGCGGTGTATATTCAAATTCTTTTCAA  ATTTACAGGGTA |
| Unigene8992 | 970 | 0 | 0% | CTCCCATTATTACTTGCGTTGTGTCTAATAAATATTCGAAATACAACACC  CCAAACCTCATATAGTTAATTCCATGGTTAGAATTTAGTTTGAGAAGGCA  GAGTCCAATTAGAGTCCTATCTAGCCAAGTGGAGGGAAGAGACTTATTAT  ACACTCAAATCAGATAAAAATACAACAGCAGAGCGGCTGATGAGTACTTG  GCAACAAACTTACATAGGTAGTGAGGCGGGGGTGAGTGTGCCTGGCGCTT  ATAAGTATTGCTGGTTCCTGAACAACCACCTGCCTGTTGGATGTGCTATT  TTAGCAAGGAAAGGGTAGCCATTGCTCCCCCAGATGTGTCAGTGCCATTC  TTCAATGCTGCAATCTCGCTTTCTAGACGCTCTTTTTCCTCTCTAAGTGC  CCTTTCCTTCTCATGGAGGGTCACTATTGATTCCATCATCAGTTGTGTCT  TTCTAGCTCTGGTTTGTGTCAGTGCAGCATCAAGTTGTTTCTCCAGTTGG  ACAAGACAGGTCACAGTCATCTGCTCAAAGTTTTGCCCTTCAAGTTGCCT  TTGAACTATTTGCAACAACTGAGCATGTGAATGCAGACTTGAATGTTTAG  ATTGAGAGCCCTCTGCATTAACACTGTTCACAGCCAGAGCTTCCTCTTCA  AAGCGACTCCGATAGCGGTCCAGGATGCTGGGCAAACTGTTGGCGCTGGA  GAACTCGTAGAGCTTGCCGCGGCTGGAGAAGATGACGAGAGCGACATCGA  CGTCGCAGAGGACGGCGAGCTCGCGAGCCTTCTTGATCAATCCAGTACGG  CGCTTGGAGAAGGTGACCTGGCGGCTGCTCTTGTTCTCGATTCGCTTCAG  CTGCAACTTCCTCCGGCCCATTCCGGTCCAATTCAGGGAGAGAATTCTTT  TTCTGTTTTTCGTTGTTGTTGTTTTAGGTTACACTGGTTGGGATATTTAA  ATTGTGCAAATTAATTAACG |
| Unigene10736 | 1206 | 0 | 0% | GGCGCTACTAAAACATTATAACTTCTGGTTTACATTGGAAAATCAAAGCA  AAAATAATACAGTAAGGCAAATTATTTTATGTTCAACTGGAAGTCATTAA  TGGATATTTTTACAAACTAATCACATTTTGACTGATAAGGCTACCTATTG  CGCATTTCCGGGAGTCCAATGAAGAGTTCAGTCTCAACGTCAGACGTATG  GCAACTTTGGCTGCAATTTGTTACAGCTTCTGTTTGTTTTGTTGATGGCT  GCAATGGACTTGCTTCACACTTTTTACATAAGCTTGCATTTTCTTCCAAC  AAGAGTCTCTCCTTTTCTTTTAGTTGCTCTCTCTGCTCGTTGAATAGCTG  ATCCTTTCTTGCCCGAATGATTCGCAGGCTCCTTTCCAGTTGGCCATCAA  TCTCATAGAGGTCATCAACACTACATGATTCCAGATCTTGCCCTAAAAGC  CTCCGCTTAGAAACTTCAAGAAGTTCGATCTTCTTCACCATATTTTCTAT  ATCTTGCTTCACTTGCTCCACGTATTGTTCCGTATGAGGCCTGTCAATTT  GCCCATCATTTTTCTTTTTACGGTATCGTTCCAATGTCTTCTGCATGTCA  GAACTTGAAAATTCATAGAGTTTTCCTTTCTGTGAGAAGATTATGGCAGC  AACTTCTGCATAAAAGAGAACTGATAGCTCATAAGCCTTCTTCAACAAAC  CGTTTCGACGTTTTGAAAAGGTCACTTGTCGGCTTGTAGCATTCTCGATT  CTCCTCATCTGGATTTTTCCTCTCACCATATGTTCGATTGTATAGAAGCA  GCTGCTCTTCACTAATTACAAGAAATGAGAATCAAGAGATGTGAGGAGGA  GAGAAAATTAAATTAGAAGAGAGAAAAATTTGTGTTGATTGAATTGTGGA  GGGGAATACTACAAGACAGAAAAGAGAAGAAGGTGCAGGAGGAGAATG  AAAGCAGTGCAAAAATGGAAATACTTGGAAACCCTAGAGAGGTGTAAGG  CAGAGGATCTTTTGATGATGGTTGATGATGTCTCATTCTCTTTATACACAAAC  CAGAAACATATGTTTTGCTGATTATTCGTAATTTTATTTTTATTTTTCAC  TGCTGTTTCAATTTAGTTCTTTGCCTCACAAAAAAGGAAAGCATAAATCC  AAGTGAACATCACTCAGAGACAGAGAGCGAGAGAGTGTTGGGAGGACA  AAATGAAG |
| Unigene13279 | 1054 | 0 | 0% | TAATTAGGTAGCACTATAATCATAGTACGTAGTCCGAAATCAACAAAATG  GAGAAGACAAACATGCATACACTTACTAATATATACCATTAATTACCATT  GTTGCAACTATTATTTGCTTGCGCCGAGAGGGAGCGAAATTGAATGCAAA  GATATGAAAAACGGCGGCCTCACATTTGCAAGGCTTTGCATGATCACACA  AACTAGTTAGGTTGTGGTGGTGGTGGCCGCGGCAAATACCTTGTTCTTCT  AGCTGGGGGCAGTCCGATGAATAATTCAGTCTCCACATCAGAAGTTGGAC  TACTGTCTTCATTGCTGGGTGAATTTTCTGGCTGCTCTTTCTCTTTCTCC  TTCTCTTTTGATCCTAGCCGCCATGGTTGTACACCACACTTTGCAGAGAG  CCTCGCATTTTCAGCTATAAGGTTCTTCTCCTTTTCTTTCAGCATCTCAA  TTTGTTCCTTGAAAACTTGAGACTTTCTTGCTCGAATGCAGTTTACGCTC  CTCTCCAGCTGTTGTTCTATCTGTTGTAGTTCTTCAATAGAGCATGATCC  TAGGCCTTCTCCTAATAGTCTCCGTTTTGCAACTTCGAGAAGCTCTATCT  TCTTCATCATGTTAGCTGCTTCATTCTTCATTTGCTGCATGCTTTGTTCT  GTTGGCTTGTCAATGGTTCGAGTATCTTTTGTATGCGTCAGATATCGTTG  TATTGTCTCCTGCATGCTGGAATTTGCAAATTCATAGAGCTTGCCTCTTG  GAGAGAAGATGATGAGAGCAACCTCGGCATCACAAAGAACTGAGAGCT  CAAATGCCTTTTTCAGCAGCCCATTTCGCCGCTTAGAGAAGGTGACTTGTCT  GCTTGTAGCATTCTCTATCCTTCTCATTTGGGTTTTCCCTCTCACCATTT  TTCTACACAAATTTATTAAAATAAGAAAAAGAACCCAGATAGAGAAAGAA  GAGACCAAAAATGGGTAGAAACTAAGATCAAATAATCTGAAGAGTTTTGA  ACAAAGATTGTTGCAGAGAAAAGCAATTACTAGGTTGTTGTCTGATTGAT  TGAC |
| Unigene15717 | 1083 | 0 | 0% | CTTAGAATATTTTCCCCAAGTTCTCTGTCTCCCTCTCTCTCTGTCTCTCT  CTCTCTTTCTGCATTTGTGAGGTGGGGTGGTGGTGGGGGTCTGAGTTTGT  GATCATCATCATGGGGAGAGGTCGGGTGCAGCTGAAGAGGATAGAGAACA  AGATCAACAGACAGGTGACTTTCTCGAAGAGAAGGTCTGGCTTGTTGAAG  AAAGCTAATGAGATCTCTGTGCTTTGTGATGCTGAGGTTGCTTTGATTGT  CTTCTCTAACAAAGGAAAGCTCTTTGAATACGCCACAGATTCTTGCATGG  AAAGTATCCTTGAAAGGTATGAGAGATATTCTTATAATGATAGGCAGCTT  ATTGCCAATGGGGTAGAACCACAGAATGGAAGCTGGACTCTGGAACATGC  AAAGCTGAAAGCAAGGGTGGAGGTTTTGCAAAGAAATCAAAAGCATTACA  TGGGTGAAGATCTTGACTCCCTAAGTCTCAAAGAACTTCAGAGTTTGGAG  CAGCAGCTTGATTCTGCTCTTAAACACATTAGGACAAGAAAGAACCAACT  CATGTTTGAATCCATTTCGGACCTTCAGAAGAAGGACAAATTATTGCAAG  AGCAAAATAACTTGCTTGCTAAAAAGGTGAAGGAAAAGGAGAAGACAAT  GACTCAACAACAACAATGGGAGCAGCAAAATCCGGGCCCGAACTCGTCC  ACTATTCTTTTAAGGCAGCCACCTCAGTCCTTAAACAATAGTGCAACTTACC  AAATGGCAAGGACCAGCGGAGGCGATGATGATGAGACTCCACAAAATCGA  GCTAATACAATCTTGCCCCCATGGATGCTTCGCAACCTTAACGAATAAGA  GAGCACAAACTCATCTTCTTAGTATGTATGCAACATTTTATTCTAAAGTA  GACTAATATATATATGGATGACTCGTATTCTAGTGATGTAAAGAGGTTGC  TGCAAAATGTTGATATTTAGTATAACATATAATAATCATATAAGGGTTTG  TATCACTATGTTCACAGCATTGCAAATTTCGTACCTTTGTATGTATTGTA  TACCCCATATGACCACAACACTAAAATGCAGAG |
| Unigene14549 | 330 | 0 | 0% | AAAGATGCGACACTACGTCCACTGCTATGCATTGCACTGCCTGGACGAGG  AAGCATCCAATGCATTGAGGAAAGCTTTCAAGGAGAGAGGAGAGAACGTA  GGGGCGTGGAGACAGGCGTGTTACAAGCCTCTTGTTGCCATCGCTGCCCG  TCAGGGTTGGGACATTGATACCATTTTCAATGCACATCCTCGACTCGCCA  TCTGGTATGTTCCTACTAAGCTTCGCCAACTCTGCCATGCCGAGCGTAAC  ACAGCCACAGCTTCTAGCTCTGCTTCCGGTGGTGGTGATCAACTGCCCTT  CTGAAGCTTGAGTCTCTGATATCGAATTTG |
| Unigene6027 | 525 | 0 | 0% | AAACAAAACAACGCTTAACTTCTCTAGTATACTCTCTCTTTCTGATCTCT  CTCACATGGAAGCAAAATTGTTTGAGCCTCTTGTGGTTGGAAAAGTAGTT  GGAGATGTTGTTGATTATTTCAATCCATCTGTGAAAATGACAATCACCTA  TAACTCCATGAAGCAAGTATATAATGGCCATGAGCTTTTTCCCTCCGCTG  TGACTCAGAAACCCAAGGTTGAGGTTCATGGAGGTGATTTGAGATCTTTT  TTCACTCTGGTTATGACTGACCCAGATGTTCCTGGTCCAAGTGACCCATA  CTTGAGGGAGCATCTACACTGGATGGTGACAGATATCCCAGGCACAACAG  ATGTCACGTTTGGAAAGGAGGTTGTGAGCTATGAAATTCCAAGGCCAAAC  ATAGGGATTCACAGGTTTGTGTTTCTTCTCTTCAAGCAGAAACGAAGGCA  GACAGTTACCAGCTTACCAGCTTCAAGAGATTGCTTTAACACTAGAAACT  TCGCTGAAGAAAATGAGCTTGGTCT |
| Unigene6475 | 1241 | 0 | 0% | CTATTTTAGACTTCCCAGGTCTTAGAGGTATATAACCTATTCCTAGATCT  AGTTAGGTAATGCCTTGAAGAGTACTATAAATACGGCACTTCTGATCTCA  TGCCATACAAGGAACTCTTACAGTTGTGAGTGGTTCCATAGATCCTGAGT  AGTTCTATTTCTATAGTTCTATCTTCTTAGGATTCCTCTCTCTCTCTATC  TCTCTCATACACACACACAGCTCTCTTGTAATGGACTCAAGAATGCAAGA  CCCTCTTGTTGTTGGGAGAGTCATTGGAGATGTTCTTGAAGCTTTCACTC  CCAGCATAAAAATGTCTGTAACTTTCAACAACAAACAAGTCTGCAATGGA  CATGAGTTCTTACCTTCTGCAGTTACCAACAAACCTAGGGTTTTGATTCA  AGGAGCTGATATGAGATCCTTCTTCACTCTGGTCATGACAGACCCTGATG  TTCCCGGCCCAAGTGATCCTTATTTAAGGGAACACCTGCACTGGATAGTG  TCAGACATTCCAGGGACAACAGATGCCACATTTGGAAAAGAAGTGGTGGG  CTACGAGATTCCAAAGCCAAATATAGGGATCCACAGGTTCGTGTTTGTTC  TTTTCAAGCAGAAATGTAGGCAGACAATTAGCGCACCTCCTTCCAGGGAC  CAATTCAGCACCAAAGCTTTCGCTGCCGAGAACGACCTTGGCCTTCCTGT  CTCTGCTGTCTACTTCAATGCACAACGAGAAACTGCTGCAAGAAGACGCT  GAAAAAACCAGATTATCATCAGTTCTCAAGAACCCCATCTATTACATGGA  TCAACCCCTCCTCACTACCTATCTTCAATAAAATAATAACAAAAAAAAAA  AAAAAAACTAGGTATTGTCCGTTAAGTATTTCTGTTGTTGTCCAGAATGA  CCCACTTTTATATATGTATATAACCTAAATAAGTTGCAGCGAGCTAGAAA  GGGAGCCAGCCAGTCCGTCTGCGTGCGTCCTGCACTGTCATTTTTACGCT  AGTGAAGTTGTGATAAATGAAGTAGTGATGATGGGTCTTTCAAGTTTCAA  GGTCCAGATTGGAATCATTAGGGGACACCTGGCACATGTTGGTTGGGCAG  GCCCTCTAAAGTTTCTCTATTATTTCAGTTCTAGGGTTTTTGTCCTATGT  GTAATATGGTGTTAAGTATGATTATTAAAAAAAATGTGGGCATTTGAAGA  TCAGGGTCAAGCTTTGTTATCATTTGATTTCTTTTTATCAC |
| CL2719.Contig1 | 1271 | 0 | 0% | GCTTTTTTCAATACGTTCTCACAAGACGCACACTTATAAATATCCAGAAT  TATGATTCCATATTTCCATGCATGAACATAAAATAACATCAAGCACTTCT  AGGTGAGTAGGATGTTACATTACAAACAATAGCAGTAAATTTGAAGAGAA  AAGAAGGTCCGTGTCGTTGGATCTGTGCAAGTTACACAAATTCCTAGCTG  TTAACACGGCCTGGCTAGGTTAGATTTTGATTTTCATTACTAGATACCTG  TCAACGTTCCAAGAAAAGAGGCCATAGCTCTACCTTTTGTGGATAAATAT  AACACCTCCAACCTTCGCTTTTAACCCCCATAGGGTAAGCCTAACTTGAG  AGATGTATCTGAGCTTTCATAGTCATGTGGAGGACCAGTGGAATTGCAAG  GATTGGTTACTGACTCTGATGACTGGCCTTCCTCATAAACCACATTTTCC  AAATCACCAACATGTCTCCGGCCGCTAGTGATCTCCACCACTTGCTGTCT  TAAACGTTCATTATCTTCCATCAATTGAATCCCCTTTGCCTGAAGGTGGC  TGATCTCTTTCATAATCTTTTGACCCTTTTTTTCTATCACCCTACTTAAT  CCAACTTCAAGAGACTTTTCTAGCACCTGCAGTTCTTCAATATTCAATGC  TTGAAGCTCTTCTCCCCTCAATTGCCTCAGTCGGTGACTCTTGTCAGCAA  TTTCCTTACTCAACCTGGTGTAGCTGCTGTTCTCTACTAACTGCAACTCA  AGAGAGGGTTGATCCAGCTTCTCAAGGTTCTTGGAGTGCAAATTGTGCCT  TTCTAATATTTCCTTCATGCTGGAGCTGGAATATTCAAAGAGCTTGCCGG  TAGACGAGAAGATGATGAGAGCAACATCGGCGTCGCAAAGAACAGAAAG  CTCTTCAGCTTTCTTGAAAAGCCCTCTTCGTCTCTTTGAGAAAGTAACTTG  TCTGGCCGTTACGTTGTCGATCTTCTTGATCTGAATCTTCTCTCTCGCCA  TTTCTCTCTCTATTTCTCCTTCCTTGAAACGCTGTTTTTGTCTTCCAGTT  TTGGGGGGACTTTGGACTGGGTCGCGTATTTTTATGTTTTTTTAATGTTT  TTGTTTCTGATTTGTGAGAGCTGGGGAGCTGCGTTGCGTTGTCTGTGGAA  AAGCCTTTTCTTTTTTTAATGTTTTTGTGTTTGGTATGTGAGATTGGGAG  TTGTGTTTGTGTCTTGCCCGTGGAAAAGCCGCCAATGGATGGATGTGTGT  GAAAAAGAGGGGAGTTTTTTA |
| CL2719.Contig2 | 1367 | 0 | 0% | GCTTTTTTCAATACGTTCTCACAAGACGCACACTTATAAATATCCAGAAT  TATGATTCCATATTTCCATGCATGAACATAAAATAACATCAAGCACTTCT  AGGTGAGTAGGATGTTACATTACAAACAATAGCAGTAAATTTGAAGAGAA  AAGAAGGTCCGTGTCGTTGGATCTGTGCAAGTTACACAAATTCCTAGCTG  TTAACACGGCCTGGCTAGGTTAGATTTTGATTTTCATTACTAGATACCTG  TCAACGTTCCAAGAAAAGAGGCCATAGCTCTACCTTTTGTGGATAAATAT  AACACCTCCAACCTTCGCTTTTAACCCCCATAGGGTAAGCCTAACTTGAG  AGATGTATCTGAGCTTTCATAGTCATGTGGAGGACCAGTGGAATTGCAAG  GATTGGTTACTGACTCTGATGACTGGCCTTCCTCATAAACCACATTTTCC  AAATCACCAACATGTCTCCGGCCGCTAGTGATCTCCACCACTTGCTGTCT  TAAACGTTCATTATCTTCCATCAATTGAATCCCCTTTGCCTGAAGGTGGC  TGATCTCTTTCATAATCTTTTGACCCTTTTTTTCTATCACCCTACTTAAT  CCAACTTCAAGAGACTTTTCTAGCACCTGCAGTTCTTCAATATTCAATGC  TTGAAGCTCTTCTCCCCTCAATTGCCTCAGTCGGTGACTCTTGTCAGCAA  TTTCCTTACTCAACCTGGTGTAGCTGCTGTTCTCTACTAACTGCAACTCA  AGAGAGGGTTGATCCAGCTTCTCAAGGTTCTTGGAGTGCAAATTGTGCCT  TTCTAATATTTCCTTCATGCTGGAGCTGGAATATTCAAAGAGCTTGCCGG  TAGACGAGAAGATGATGAGAGCAACATCGGCGTCGCAAAGAACAGAAAG  CTCTTCAGCTTTCTTGAAAAGCCCTCTTCGTCTCTTTGAGAAAGTAACTTG  TCTGGCCGTTACGTTGTCGATCTTCTTGATCTGAATCTTCTCTCTCGCCA  TTTCTCTCTCTATTTCTCCTTCCTTGAAACGCTGTTTTTGTCTTCCACCA  AAAACACAAGCACAAAACAAGAAATGTTTCTATATATAGGGACTGGTCAG  GCCAAAAAGTGGCAGCATTGTTGCCAAATTTAGAGGACAATCAGTTTTGG  GGGGACTTTGGACTGGGTCGCGTATTTTTATGTTTTTTTAATGTTTTTGT  TTCTGATTTGTGAGAGCTGGGGAGCTGCGTTGCGTTGTCTGTGGAAAAGC  CTTTTCTTTTTTTAATGTTTTTGTGTTTGGTATGTGAGATTGGGAGTTGT  GTTTGTGTCTTGCCCGTGGAAAAGCCGCCAATGGATGGATGTGTGTGAAA  AAGAGGGGAGTTTTTTA |
| CL2719.Contig3 | 1532 | 0 | 0% | GCTTTTTTCAATACGTTCTCACAAGACGCACACTTATAAATATCCAGAAT  TATGATTCCATATTTCCATGCATGAACATAAAATAACATCAAGCACTTCT  AGGTGAGTAGGATGTTACATTACAAACAATAGCAGTAAATTTGAAGAGAA  AAGAAGGTCCGTGTCGTTGGATCTGTGCAAGTTACACAAATTCCTAGCTG  TTAACACGGCCTGGCTAGGTTAGATTTTGATTTTCATTACTAGATACCTG  TCAACGTTCCAAGAAAAGAGGCCATAGCTCTACCTTTTGTGGATAAATAT  AACACCTCCAACCTTCGCTTTTAACCCCCATAGGGTAAGCCTAACTTGAG  AGATGTATCTGAGCTTTCATAGTCATGTGGAGGACCAGTGGAATTGCAAG  GATTGGTTACTGACTCTGATGACTGGCCTTCCTCATAAACCACATTTTCC  AAATCACCAACATGTCTCCGGCCGCTAGTGATCTCCACCACTTGCTGTCT  TAAACGTTCATTATCTTCCATCAATTGAATCCCCTTTGCCTGAAGGTGGC  TGATCTCTTTCATAATCTTTTGACCCTTTTTTTCTATCACCCTACTTAAT  CCAACTTCAAGAGACTTTTCTAGCACCTGCAGTTCTTCAATATTCAATGC  TTGAAGCTCTTCTCCCCTCAATTGCCTCAGTCGGTGACTCTTGTCAGCAA  TTTCCTTACTCAACCTGGTGTAGCTGCTGTTCTCTACTAACTGCAACTCA  AGAGAGGGTTGATCCAGCTTCTCAAGGTTCTTGGAGTGCAAATTGTGCCT  TTCTAATATTTCCTTCATGCTGGAGCTGGAATATTCAAAGAGCTTGCCGG  TAGACGAGAAGATGATGAGAGCAACATCGGCGTCGCAAAGAACAGAAA  GCTCTTCAGCTTTCTTGAAAAGCCCTCTTCGTCTCTTTGAGAAAGTAACTTG  TCTGGCCGTTACGTTGTCGATCTTCTTGATCTGAATCTTCTCTCTCGCCA  TTTCTCTCTCTATTTCTCCTTCCTTGAAACGCTGTTTTTGTCTTCCACCA  AAAACACAAGCACAAAACAAGAAATGTTTCTATATATAGGGACTGGTCAG  GCCAAAAAGTGGCAGCATTGTTGCCAAATTTAGAGGACAATCTGTGAGAC  AAACCCTAATTAAGTAAGAGAGAGACTCGTAATTTTTTCAGATCTTGAAG  AAGAATAAAAACTGAAAGCCCCACCAATCGCAGGCCACGCAGAGAAG  GGGAAAATACAGAAATTGGCTGAGGCAAGATATTAATTGCATTCCATTTTGCA  CCTTACCAGTTTTGGGGGGACTTTGGACTGGGTCGCGTATTTTTATGTTT  TTTTAATGTTTTTGTTTCTGATTTGTGAGAGCTGGGGAGCTGCGTTGCGT  TGTCTGTGGAAAAGCCTTTTCTTTTTTTAATGTTTTTGTGTTTGGTATGT  GAGATTGGGAGTTGTGTTTGTGTCTTGCCCGTGGAAAAGCCGCCAATGGA  TGGATGTGTGTGAAAAAGAGGGGAGTTTTTTA |
| CL645.Contig1 | 970 | 0 | 0% | GATTTTAAACAGAGATCACAGGTGTCCATCAAAGGTCGGGTTAACCAATC  TAGTAAAGAGAATCATTCACTTCTTTATTTGGCTATAGACAGAATGTTCT  TCTTCTCTCTCATTCCAGAACTAGGTTTCTCTCTTTCTAAAAGCCATTGG  TAAATCTGGTATCCGGTTTACTGTCAGGTGTCACCGGCTCCCATTTAAAG  AAAAAACAAGGGTGGGCTCGATTTTGTGGGTAAAAATGACGAGACAGAA  AATTGAGATCAAGAAGATCGACAACACCACGGCCAGGCAGGTTACATTCT  CAAAAAGGAGAAGAGGGCTTTTCAAGAAGGCACAGGAGCTCTCAACTCT  CTGTGATGCTGAAATAGCTCTCATGGTCTTCTCTGCCACAGGAAAGCTCTTT  GAATACTCCAGCTCAAGTATGAAACAGGTGATTGAAAAATACAAGACGAA  TATGCAGAATCTTCACAAATTCGATCAACCATCTCTGGAATTACAGCTTG  AATGCAATACCTACGCCATGTTGAGCGGAGAAATAGCCGAAAAGACCCGT  GAGCTAAGGCAGATGAGGGGAGAAGAACTCCAAGGGTTGAGCTTAGAGGA  GCTAAAGCAACTAGAAAAATCACTTGAAGGAGGGTTAAGATGTGTGGCGG  AAATGAAGGATGAAAGAATGATGAAAGAAATTAGTGATCGCAAGACAAGG  GAGCAACAACTGATGGATGAAAATCTGAGACTGAGACAAAGAACAGGGA  ATTTAGATTCACATGTACAAGGCCAGTCGTCAGAGTCAGTCACCAATATCA  GCAGCTTAGGAGATGTTCCTCAAGACAATAACAGCTCTGATACTTCTTTA  AAGTTGGGGTTAGCATTTCCTAGCTAACATTGGAGATGGTGATCATTACC  AACAATAGACGGCTTTGGACTAACTTATTACTCTCTCTTCATATACATAT  ATATACATAAAATAACCAAG |
| CL645.Contig2 | 1259 | 0 | 0% | GATTTTAAACAGAGATCACAGGTGTCCATCAAAGGTCGGGTTAACCAATC  TAGTAAAGAGAATCATTCACTTCTTTATTTGGCTATAGACAGAATGTTCT  TCTTCTCTCTCATTCCAGAACTAGGTTTCTCTCTTTCTAAAAGCCATTGG  TAAATCTGGTATCCGGTTTACTGTCAGGTGTCACCGGCTCCCATTTAAAG  AAAAAACAAGGGTGGGCTCGATTTTGTGGGTAAAAATGACGAGACAGAA  AATTGAGATCAAGAAGATCGACAACACCACGGCCAGGCAGGTTACATTCTC  AAAAAGGAGAAGAGGGCTTTTCAAGAAGGCACAGGAGCTCTCAACTCTC  TGTGATGCTGAAATAGCTCTCATGGTCTTCTCTGCCACAGGAAAGCTCTTT  GAATACTCCAGCTCAAGTATGAAACAGGTGATTGAAAAATACAAGACGAAT  ATGCAGAATCTTCACAAATTCGATCAACCATCTCTGGAATTACAGCTTG  AATGCAATACCTACGCCATGTTGAGCGGAGAAATAGCCGAAAAGACCCGT  GAGCTAAGGCAGATGAGGGGAGAAGAACTCCAAGGGTTGAGCTTAGAGGA  GCTAAAGCAACTAGAAAAATCACTTGAAGGAGGGTTAAGATGTGTGGCAG  AAACGAAGGTTGAGAGAATTATGAAAGAGATTAGTGATCTCAAGACAAGG  ACAGGGAATTTAGATTCACATGTACAAGGCCAGTCGTCAGAGTCAGTCACC  AATATCAGCAGCTTAGGAGATGTTCCTCAAGACAATAACAGCTCTGATA  CTTCTTTAAAGTTGGGGTTAGCATTTCCTGGCTAACATTGGAGCTGGAGA  TCATCACCAACAATAAACGGCTTTGGACTAACTTATTACTCTCTCTTCAT  ATACATATATATACATAAAATAACCAAGTGCGTCTCATAGTTTGTACCTA  AATTCATGTAGGTTCAATTGATTGTTGCTGGTTTTATCAAGAGATTATTT  CTGAGCATTACAATTAACAACTTGGAATGCTTGGAGAGATCAAGTAATAA  TTTTCAAGTGTGGGACCCATTGCAGTTCAATTTGATGTAGAAGCAGAAAC  GATGAATGTATGGAAGTCTGAAATTTCACACTTGAGAAATATCATTTGAT  CGAGTGTATGTAAGTGTTTGCTTATTTATTTCAATGAAGTAAGTTGAAAA  TGTGTGTATAACCAATGGAGACCATATAATATGAGAACGATAAGATATTA  ATTTTCAGC |
| Unigene17296 | 897 | 0 | 0% | ACCCAATCCCTATATCTACATCGCTAATCCTTCCTTTGAAACTTTGTTTA  CTAATTAATGAGCTTGGATGGATGGACAGGCTTCTTCTTCATTTCACTTA  CCAAACTTATAGTTTTTTTCTTCTTTTTGCCATCTAGGGGTGGGCTCGAT  TTTGTGGGTAAAATGACGAGACAGAAAATTGAGATCAAGAAGATCGACAA  CACCACGGCCAGGCAGGTTACATTCTCAAAAAGGAGAAGAGGGCTTTTCA  AGAAGGCACAGGAGCTCTCAACTCTCTGTGATGCTGAAATAGCTCTCATG  GTCTTCTCTGCCACAGGAAAGCTCTTTGAATACTCCAGCTCAAGTATGAA  ACAGGTGATTGAAAAATACAAGACGAATATGCAGAATCTTCACAAATTCG  ATCAACCATCTCTGGAATTACAGCTTGAATGCAATACCTACGCCATGTTG  AGCGGAGAAATAGCCGAAAAGACCCGTGAGCTAAGGCAGATGAGGGGAG  AAGAACTCCAAGGGTTGAGCTTAGAGGAGCTAAAGCAACTAGAAAAATC  ACTTGAAGGAGGGTTAAGATGTTTGGCCGAAACGAAGGGTGAAAGAATTA  TGAAAGAAATTAGTGATCTCAAGACAAGGGAACAACAACTGATGGATGAA  AATCTGAGACTGATACAAATAACAGAGAATTTAGATTCACATGTACAAGGTC  AGTCGTCAGAGTCAGTCACCATTATGGGCAGCTTTGGGGATGTTCCTCAA  GACAGTAACAGCTCTGATACTTCTTTAAAGTTGGGGTTAGCATTTCCTGG  CTAACATTGGAGCTGGAGATCATCACCAACAATAAACGGCTTTGGACTAA  CTTATTACTCTCTCTTCATATACATATATATACATAAAATAACCAAG |
| Unigene26608 | 418 | 0 | 0% | AGTGTGGAATACGCATTCTTTGCAGTTGCTGAGCTCATGGGTCTTTTTGG  CAATTTCTCTGCTCAACATAGCATAGGTATTGCATTCAAGCTGTAATTCC  AGAGATGGTTGATCGAATCCGTGAAGATTCTGCATTTTCATCTTGTATTT  TTCAATCACCTGTTTCATACTTGAGCTGGAGTATTCAAAGAGCTTTCCTG  TGGCAGAGAAGACCATGAGAGCTATTTCAGCATCACAGAGAGTTGAGAGC  TCCTGTGCCTTCTTGAAAAGCCCTCTTCTCCTTTTTGAGAATGTAACCTG  CCTGGCCGTGGTGTTGTCGATCTTCTTGATCTCAATTTTCTGTCTTGTCA  TTATTTTACCCAAAAAATCGAGCCCACCCTTGTTTTTTCTTTAAATGGGA  GCTGGTGACACCAGACAA |
